# The development of *Drink Less*: an alcohol reduction smartphone app for excessive drinkers

**DOI:** 10.1093/tbm/iby043

**Published:** 2018-05-04

**Authors:** Claire Garnett, David Crane, Robert West, Jamie Brown, Susan Michie

**Affiliations:** 1 Research Department of Behavioural Science and Health, UCL, London, UK; 2 Research Department of Clinical, Educational and Health Psychology, UCL, London, UK

**Keywords:** Development, Digital interventions, Smartphone application, Alcohol consumption, Behavior change

## Abstract

Excessive alcohol consumption poses a serious problem for public health. Digital behavior change interventions have the potential to help users reduce their drinking. In accordance with Open Science principles, this paper describes the development of a smartphone app to help individuals who drink excessively to reduce their alcohol consumption. Following the UK Medical Research Council’s guidance and the Multiphase Optimization Strategy, development consisted of two phases: (i) selection of intervention components and (ii) design and development work to implement the chosen components into modules to be evaluated further for inclusion in the app. Phase 1 involved a scoping literature review, expert consensus study and content analysis of existing alcohol apps. Findings were integrated within a broad model of behavior change (Capability, Opportunity, Motivation-Behavior). Phase 2 involved a highly iterative process and used the “Person-Based” approach to promote engagement. From Phase 1, five intervention components were selected: (i) Normative Feedback, (ii) Cognitive Bias Re-training, (iii) Self-monitoring and Feedback, (iv) Action Planning, and (v) Identity Change. Phase 2 indicated that each of these components presented different challenges for implementation as app modules; all required multiple iterations and design changes to arrive at versions that would be suitable for inclusion in a subsequent evaluation study. The development of the Drink Less app involved a thorough process of component identification with a scoping literature review, expert consensus, and review of other apps. Translation of the components into app modules required a highly iterative process involving user testing and design modification.

## Teaser

Apps that provide feedback on drinking alcohol appear to be reaching those who would benefit from support.

**Implications**

**Practice**: The development process for the *Drink Less* app could provide a practical model for developing evidence- and theory-based digital interventions for health-related behavior change following the principles of Open Science.

**Policy**: Policy makers should promote the adoption of open and transparent methods for the development of digital healthcare interventions to help assess how far they are likely to meet their objectives ahead of testing in randomized trials or other appropriate evaluation methods.

**Research**: The reporting in full of the approach used to develop digital healthcare interventions such as *Drink Less* provides a firm foundation for interpreting the results of evaluation studies that follow.

## INTRODUCTION

Excessive alcohol consumption poses a serious problem for public health [1, 2]. About 3.3 million deaths are attributable to alcohol consumption worldwide each year [1] and alcohol consumption is the third leading cause of morbidity and premature death in high-income countries [3]. Over 5 per cent of the global burden of disease and injury is estimated to be attributable to alcohol [4]. Excessive alcohol consumption (widely indicated by a score of 8 or above on the Alcohol Use Disorders Identification Test [AUDIT] [5]) is estimated to cost high- and middle-income economies 2.5 per cent of gross domestic product due to costs associated with health and social care, the police and criminal justice system, and lost productivity [6].

Digital behavior change interventions (predominantly web-based) have the potential to reduce excessive alcohol consumption [7]. They can reach large numbers of people for a low incremental cost of provision, offer convenience and privacy for users, and may reduce the stigma associated with help-seeking in person [8]. Smartphone ownership is increasingly prevalent; for example, 77 per cent of U.S. [9] and 71 per cent of UK adults [10] own a smartphone. The tendency for smartphones to be carried much of the time [11] and used repeatedly [12] may mean that apps have the capacity to deliver support in a timely manner in the situations in which people want support [13] for however long support is required.

Many alcohol reduction apps are available [14, 15]. However, the majority are developed with no reference to scientific evidence or theory [14], some provide inaccurate information on the user’s blood alcohol concentration [15], and apps treating alcohol use disorders tend to be of low quality [16]. There are development papers for mobile and web-based interventions for alcohol reduction [17–20], and for digital interventions targeting other health-related behaviors such as smoking cessation [21, 22]. However, to the authors’ knowledge, there is no published report of the systematic development of an alcohol reduction app.

Papers that clearly report intervention development and content are central to efficient scientific progress [23] and to avoiding trial wastage [24]. The time and resources invested in conducting intervention evaluations may be wasted unless the intervention content is adequately reported [24]. Improving the description of interventions can contribute to reducing avoidable waste in health research and to the efficient synthesis of evidence [25]. In addition, clear and systematic reporting of intervention development can inform policy-makers’ decisions as to which interventions to adopt. For example, the smoking cessation website StopAdvisor was developed systematically [21] and, following a successful evaluation [26], is now being adapted and implemented by Public Health England for roll-out across the UK.

*Drink Less* is an app for the general population of adults seeking digital support to reduce excessive alcohol consumption. The development and evaluation strategy for the app followed UK Medical Research Council guidance on complex interventions [27] and the Multiphase Optimization Strategy [28]. Both approaches recommend the use of theory and evidence in the selection of intervention components and propose that the development of complex interventions is undertaken in a number of iterative phases with feedback loops [27, 28]. The Capability, Opportunity, Motivation-Behavior model was selected to inform the development of *Drink Less* on the basis that its breadth promotes a high-level assessment of the wide variety of possible individual influences on behavior, their interactions, and levers for behavior change [29, 30]. The Capability, Opportunity, Motivation-Behavior model posits that behavior results from interactions between *“capability*,” *“opportunity,”* and *“motivation”* [29, 30]. These three high-level components can be elaborated into the Theoretical Domains Framework [31]. The 14 domains of the Theoretical Domains Framework were the key influences described by behavior change theories [31, 32] and each component (Capability, Opportunity, Motivation) can be mapped onto one or more of the Theoretical Domains Framework domains [31]. Together the Capability, Opportunity, Motivation-Behavior model and Theoretical Domains Framework provide a more detailed approach to understanding influences on behavior change [31] and an overarching theoretical structure for intervention development.

An intervention component is “any aspect of an intervention that is of interest and can be separated out for study” (p. 221 [33]). Components have to be distinct from other components, individually meaningful (i.e., based on a theoretical construct or domain), independent of time-sequencing, and implementable in any combination. Components may include individual or combinations of behavior change techniques (BCTs) [34]—an observable, replicable, and constituent part of an intervention designed to change a specified behavior [35].

Intervention components can be translated into related app modules (the text, graphics, and functionality used to deliver the intervention component) using a person-based approach for intervention development [36]. Traditional user testing tends to focus on the hedonic or utilitarian qualities of a technology [37, 38], and the person-based approach seeks to understand the appropriateness of the BCTs used and the challenges faced or anticipated in adhering to them. In this way, acceptable and feasible BCTs can be identified and improved, with impractical or intrusive BCTs replaced.

This paper reports the systematic development of *Drink Less* to an appropriate stage for evaluation in a factorial randomized control trial.

## METHODS

**Fig 1:**
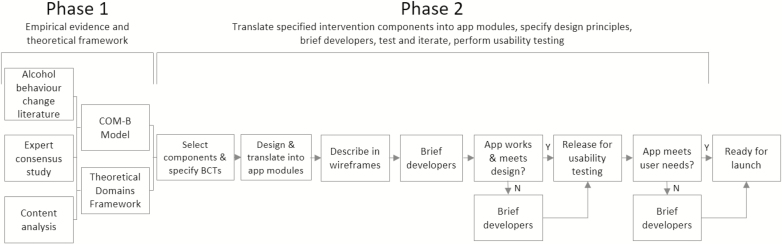
Overview of the app development process.

The development of the app consisted of the following: (i) initial selection of intervention components and (ii) design and translation of the components into modules within a coherent and appealing app. The necessary research for development was conducted at University College London, and related studies requiring ethical approval were reviewed by the UCL Ethics Committee under the “optimization and implementation of interventions to change health-related behaviors” project (CEHP/2013/508). Phase 1 of development began in January 2014 and Phase 2 began in September 2014 with the final version launched in May 2016. Figure 1 shows an overview of the app development process.

## PHASE 1: SELECTION OF INTERVENTION COMPONENTS

### Empirical evidence

The selection of intervention components was informed by an examination of the BCTs used in alcohol interventions [39] and by conducting two additional studies.

#### Expert consensus study

A formal consensus building exercise with seven international alcohol and behavior change experts was conducted to identify the intervention components and engagement strategies believed to be the “best bets” for reducing consumption in an app-based intervention. Experts were asked to rely on their knowledge of the alcohol literature, and/or experience of designing or delivering behavior change interventions [40]. The overall exercise consisted of a Delphi study with three rounds: (i) generating suggestions, (ii) rating, and (iii) ranking. Twelve intervention components and 17 engagement strategies achieved consensus among the experts as likely to be effective [40]. Self-monitoring, goal setting, action planning, and feedback in relation to goals were the intervention components judged to have the greatest potential. The strategies most likely to engage users were ease of use, design, tailoring of design and information, and unique smartphone features. This study has been published in full elsewhere [40].

#### App content analysis—most frequently used intervention components

A content analysis of popular alcohol-related apps was conducted to identify their BCTs and explore whether included BCTs were associated with app popularity or user ratings [14]. Of the 14 per cent of apps that had an alcohol reduction focus, the BCTs most frequently used were facilitate self-recording, provide information on consequences of excessive alcohol use and drinking cessation, and provide feedback on performance. This study has been published in full elsewhere [14].

### Theoretical framework

The Capability, Opportunity, Motivation-Behavior model and Theoretical Domains Framework provided an overarching structure within which to consider other theories of behavior change and were used to conduct a behavioral analysis to identify potential facilitators of and barriers to behavior change [41]. The behavioral analysis of excessive alcohol consumption involved a scoping literature review of behavior change theories to identify factors associated with excessive alcohol consumption that could be targeted by intervention components in an app. This analysis indicated that the following components were appropriate to include in an alcohol reduction app: provision of information, normative feedback, cognitive bias modification, self-monitoring, action planning, identity change, evaluation of benefits and costs of drinking, and goal setting.

The empirical evidence and theoretical framework provided multiple sources of information to directly inform the selection of intervention content for, and the design principles of, the Drink Less app (followed during the translation of intervention components into app modules). These sources of evidence provided the basis for the prioritization of intervention components for inclusion in the app. If the same intervention component arose multiple times, then there was an increased confidence in that particular component. The final decision for inclusion drew on researcher judgement. Multiple potential intervention components were identified and it was decided that the app should be used as a “toolbox” with users able to choose the components of the app that suited them best.

## PHASE 2: DESIGN AND TRANSLATION INTO AN APP

The second phase of development required the translation of the selected intervention components into modules within a single app that was coherent and appealing to use.

### Translating specified intervention components into app modules

First, the BCTs targeting the theoretical construct or domain (in terms of the Capability, Opportunity, Motivation-Behavior model and Theoretical Domains Framework) for each intervention component were selected by the research team. Using BCTs to describe the potentially active ingredients of an app enables interventions to be designed in a systematic, replicable, and comparable manner [34]. The BCT Taxonomy Version 1 [34] was used to specify the intervention components of *Drink Less*.

Second, the research team used wireframes in the form of PowerPoint slides to describe how each intervention component should be translated into an app module (in terms of the text, graphics, and functionality) to the app developers (Portable Pixels—http://portablepixels.com/). The intervention components were translated into app modules in close collaboration with expert and experienced app developers. Regular discussions between members of the research team and the developers were conducted to ensure that the description provided of the intervention components was translated in ways that were feasible for users (judged by the app developers) and met the initial specification (judged by the research team).

### Design principles

A number of design principles were followed in the development of the app. Users value a visually appealing and professionally designed app [40, 42, 43]. Digital interventions need to be easy to use and their navigation needs to be intuitive and consistent throughout the app [43]. In-app notifications can encourage users to perform actions, and gamification—the application of game-design elements and principles in nongame contexts—can increase intervention use [40, 42, 43]. Other design principles recommend that the credibility of the information provided should be illustrated [42, 43], the language should be free of scientific jargon, and the amount of text should be minimized wherever possible [43].

### Testing and iterating

An “agile” methodology for development was used as it delivers working software at regular intervals and allows the testing of individual modules before the app has been built in its entirety [44]. Testing was an extensive and iterative process that assessed whether all the elements functioned optimally from a user’s perspective. Modules went through numerous iterations between the initial description of intervention components and the version released on the app store. Screens that required user input were tested with dummy data and the app was thoroughly examined for programming bugs before being released.

Informal testing was performed by members of the research team, their friends and family, and other staff and students at UCL. Testing included the app’s text, design and functionality, the registration and randomization process (for the subsequent evaluation), and the fidelity of data storage. The app build started in September 2014 and a first version was released for testing in May 2015. Formal testing was undertaken in a usability study of user views toward the app.

### Usability testing

The usability study explored user views toward the app in order to determine whether the BCTs were acceptable and feasible to users and how they might be improved. The person-based approach to intervention development was adopted, as this emphasizes the importance of understanding users’ views toward the acceptability and feasibility of intervention components [36,45]. The usability study consisted of two parts: a think aloud study to understand users’ first impressions and semistructured interviews to investigate user’s impressions of prolonged use in naturalistic settings [46]. Participants were recruited by researchers at University College London with purposeful sampling in order to ensure the views of disadvantaged groups were gathered. The mean interview length was 59 min, and participants gave informed consent and were compensated £20 for their time. Interviews were audio recorded, transcribed verbatim, and analyzed with thematic analysis. Issues identified by multiple participants or common to both parts of the study were given priority when making changes to the app [46]. For example, many users in both studies were confused about how to navigate the app after completing the registration process. To remedy this, a guide was added that encouraged users to set a goal, enter drinks, and explore the app. Users also expressed confusion about how to use different modules within the app, so an information button explaining how to use each screen was added. This study is described in full elsewhere [46] and the full list of changes made in response to the findings is included in Supplementary File 2 (available online).

## RESULTS

**Fig 2:**
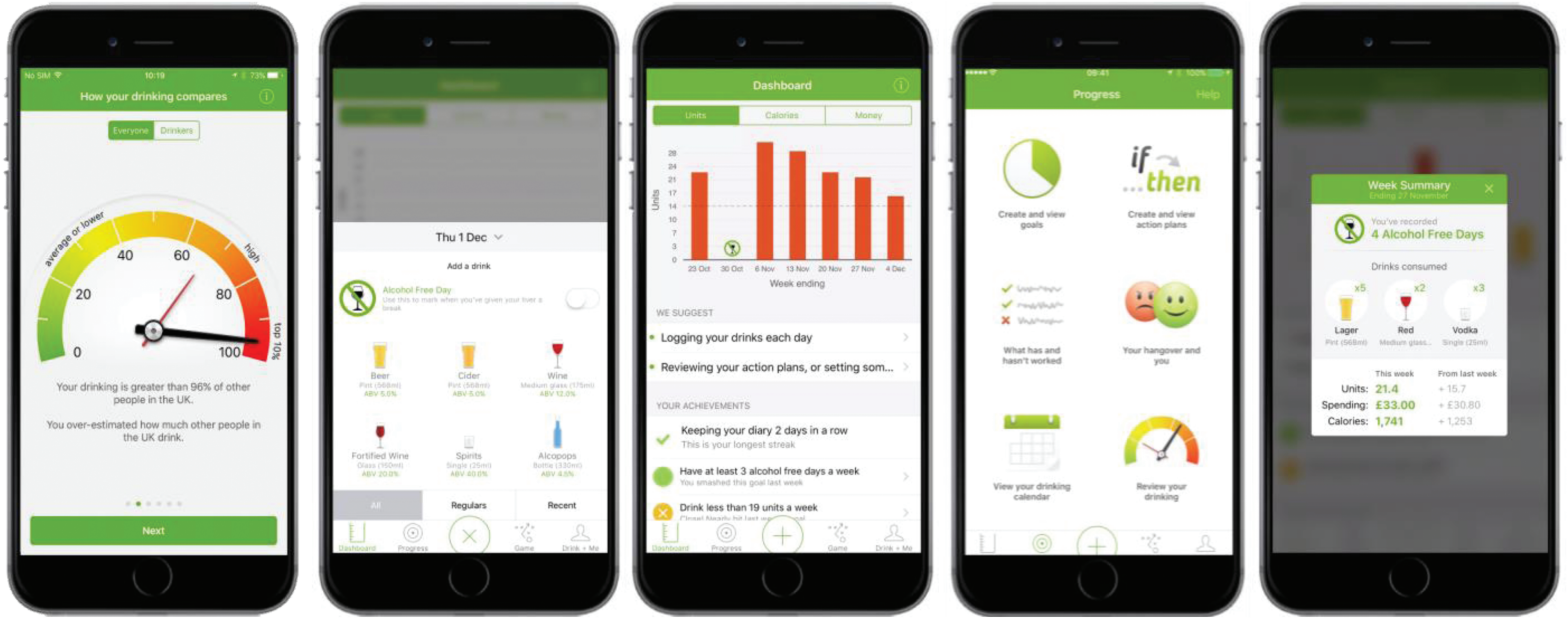
Example screenshots from the Drink Less app.

The development resulted in an alcohol reduction smartphone app that was centered around a goal setting intervention module and had five experimental modules for evaluation in a factorial randomized control trial: (i) Normative Feedback, (ii) Cognitive Bias Re-training, (iii) Self-monitoring and Feedback, (iv) Action Planning, and (v) Identity Change. In addition to the intervention modules, all users had to complete the Registration section and were given access to a Help section of the app. Example screenshots from the app are shown in Fig. 2.

## INTERVENTION MODULES

The two phases for each intervention module are presented below: (i) rationale for selection and (ii) design and translation. Supplementary Table 1 lists the theoretical constructs and domains that were targeted for each module, the BCTs used to deliver the intervention content (and the BCTs used in the “minimal” version for the experimental modules being evaluated) and the intervention module strategy/objective. Supplementary File 2 (available online) contains a full description of the intervention content with app screenshots.

### Goal setting

The overall strategy of the goal setting module was to allow users to set different weekly goals and provide information on setting appropriately specific and difficult goals.

#### Phase 1: Rationale for selection

Goal setting has substantial evidence for its effectiveness across many different behaviors and contexts [47–49]. Experts in alcohol and behavior change identified goal setting as a best bet for an intervention component in an app [40]. Goal setting has been used in a number of existing digital alcohol interventions [50] and popular alcohol reduction apps [14] suggesting its feasibility for an app-based intervention. The evidence for goal setting as an effective BCT was considered sufficiently robust to include it as a feature that all users received (and was not tested experimentally) as there was a pragmatic need to structure the app around a feature that could promote engagement and facilitate the use of other intervention modules.

#### Phase 2: Design and translation

The BCT used was “goal setting (behavior).” Users were prompted to set a weekly “drinking reduction” goal (units, spending, alcohol free days, or calories) after completing registration.

The main screen of the goal setting module asked the question “I want to drink less because…?” in order to help participants establish an overarching reason for reducing their alcohol consumption. Their responses were prominently displayed on the Dashboard. Brief text explained some of the principles of good goal setting and there were links to “Set and view goals” and “How to set good goals.”

“Set and view goals” allowed users to set a new goal and contained a list of all active goals and any previously set goals. Users could choose between four types of goal with default options that provided recommendations for potentially suitable goals: units (14 units [51]), calories (1100—approximate calorie equivalent of 14 units of average strength beer or wine), alcohol free days (3 [51, 52]), and spending (no default because the price of alcoholic drinks varies considerably throughout the UK). If a unit goal was chosen, participants could click a link to see how many units were in typical drinks. “How to set good goals” provided more information on setting goals.

Goals were automatically set to recur every Monday but participants could deselect this option if they wished. On tapping the “Save” button a confirmation message appeared to let participants know they had successfully set a goal.

### Normative feedback

Normative feedback is personalized feedback on how an individual’s behavior compares with the behavior of other people. The module informed users of the norm for alcohol consumption and alerted them to any discrepancy between the norm and their current levels of alcohol consumption.

#### Phase 1: Rationale for selection

Providing normative feedback was identified by alcohol and behavior change experts as a BCT likely to be effective at reducing excessive alcohol consumption in an app [40]. Normative misperceptions, the underestimating of one’s own alcohol use compared with others, exist in the general population [53] and greater normative misperceptions are associated with increased alcohol consumption [53, 54]. Correcting these normative misperceptions can reduce subsequent alcohol use [55]. Social Norms Theory predicts that people behave in such a way that attempts to conform to the perceived norm [56], which can result in people behaving in a manner that is inconsistent with their own beliefs and values [57].

#### Phase 2: Design and translation

Users’ normative misperceptions were assessed after completion of the Registration module. Users were compared against the following: the general population, a subgroup of their gender and age, and only drinkers for both comparison groups, based on a representative sample of the general population in England [58]. Full details of the questions assessing users’ normative misperceptions and the comparison dataset are in Supplementary File 2 (available online). Normative feedback was provided in the form of the user’s percentile in a distribution of population alcohol consumption in England and what that meant for their alcohol-related risk relative to others. Visual representations of the users’ reported drinking levels and their normative misperceptions were used to minimize the text and make the screen more aesthetically pleasing. Two separate visual representations of these data were chosen (a “gauge” and a “people infographic”) to increase the dose of the normative feedback provided.

### Cognitive bias retraining

Cognitive bias retraining is typically a computerized task that aims to retrain automatic biases, such as approach and attentional biases, away from alcohol-related cues or stimuli. The module aimed to change approach biases to alcohol stimuli through an approach-avoidance style game in which users must avoid alcohol-related pictures and approach non-alcohol-related pictures.

#### Phase 1: Rationale for selection

Automatic biases in information processing of alcohol-related cues or stimuli, such as approach and attentional biases, predict alcohol use [59]. Dual process theories of addiction suggest that excessive alcohol consumption occurs, in part, due to automatic processes when the impulses to drink overcome the inhibitory response not to [60,61]. Inhibition training is a type of cognitive bias retraining and was identified by experts as a BCT likely to be effective at reducing excessive alcohol consumption in an app [40]. Approach-avoidance training is another type of cognitive bias retraining that has been found to be effective at altering approach biases to alcohol-related stimuli and been shown to have a good efficacy in reducing subsequent alcohol consumption [62–64].

#### Phase 2: Design and translation

The game—named “Yes please, No thanks”—used approach-avoidance training in an attempt to retrain biases to alcohol cues from an “approach” to an “avoid” bias [63]. A total of 40 images were used (20 alcohol-related and 20 non-alcohol-related), selected from the validated Amsterdam Beverage Picture Set [65]. All the alcohol images were in the format associated with “avoid” (“No thanks”), and nonalcohol images were in the format associated with “approach” (“Yes please”) [66].

Users approached or avoided the images using their finger to swipe the image (up for “avoid” and down for “approach”). When a user responded correctly, the screen flashed green and a “correct” sound was played, and if an incorrect response occurred, the screen flashed red and a sound indicating an error was played. Each game lasted 1 minute. Previous scores were illustrated using a bar graph to create a sense of competition (a principle of gamification [67]) and promote further engagement with the intervention module.

### Self-monitoring and feedback

Self-monitoring is the act of noticing and recording goal-related behavior [68]. Feedback allows the current position in relation to the goal and rate of progress toward it to be determined [69]. The module enabled users to record their alcohol consumption and provided feedback on consumption, the consequences of consumption (mood, productivity, and sleep), and progress against goals.

#### Phase 1: Rationale for selection

Self-monitoring and Feedback are both recommended by NICE clinical guidance as effective techniques as part of interventions for alcohol reduction (as well as other health-related behaviors such as diet and smoking) [70]. Self-monitoring has been found an effective BCT in alcohol interventions [39]. Feedback on behavior and outcomes of behavior is a key component of face-to-face alcohol interventions [71], has been commonly included in digital interventions [7], and has been found to augment the effect of self-monitoring [72,73]. Self-monitoring and feedback were ranked highly by alcohol and behavior change experts as BCTs likely to be effective in an alcohol reduction app [40].

#### Phase 2: Design and translation

The main aim when building the module was to make the process of recording drinks as easy as possible. Large numbers of users stop using health apps if they find data entry too burdensome [74, 75] and interventions which greatly increased the frequency of self-monitoring have been found to produce small-to-medium-sized improvements in goal attainment [76].

Drinks were recorded by tapping a large link placed in the center of the bottom menu bar. Users could choose from six categories of drink and, once selected, could adjust the default entries for ABV (alcohol by volume), size, quantity, and price. An alcohol free day could also be recorded by tapping the “Alcohol Free Day” button, after which a pleasing sound and animation was played and a large green tick and “Keep up the good work!” was displayed. Users were reminded to complete a log of their drinking at 11:05 am each morning, primarily through an on-screen alert.

The reminder that asked participants to log their drinking also asked them to report on their current mood, level of productivity, degree of clarity, and quality of the previous night’s sleep. Graphs displayed differences between scores after nights of heavy drinking compared with nights of no or light drinking for each measure, with the aim that a positive difference between scores may result in users increasing their motivation to drink less alcohol.

Feedback on consumption and the consequences of consumption was provided on the dashboard. The number of alcohol units consumed per week since the app was downloaded was displayed in a graph. The calories and money consumed from alcohol during the current week and since the app was downloaded were displayed numerically. Brief feedback about performance against goal(s) in the week to date was displayed on the dashboard, and these linked to more detailed information about the progress against goals in the most recently completed week and since the app was downloaded.

In an attempt to prevent goal disengagement, positive reinforcement was provided when goals were met and motivational feedback when goals were missed. Positive reinforcement and motivational feedback were delivered in text form, for example, “Well done, you hit your goal. Keep going.” If users missed their goal by 20 per cent or more the app suggested the user might want to adjust their goal. These suggestions were only delivered after a goal had been exceeded or missed twice in a row in order to account for periods of unusual drinking behavior.

The drinking calendar displayed dates in months, beneath which were colored bars to indicate a no drinking day, light drinking day (greater than 0 units but less than 6 units), heavy drinking day (more than six units), or no record entered. Tapping a day when no drink was recorded displayed text that adhered to the Timeline Follow-back procedure [77] and prompted participants to look at their diary, text messages, or emails to jog their memory of their drinking for that day.

### Action planning

Action planning, in the form of implementation intentions, is a prespecified behavior scheduled to take place when a situation expected to present challenges or opportunities for goal attainment is encountered [78]. The module allowed users to create implementation intentions for dealing with difficult drinking situations, explained why action planning could be helpful and gave examples of alcohol-related implementation intentions.

#### Phase 1: Rationale for selection

A meta-analysis of 94 trials found that implementation intentions had a medium-to-large positive effect on goal attainment across a wide variety of behaviors [78], including alcohol reduction [79–81]. Action planning was ranked highly by alcohol and behavior change experts as a BCT likely to be effective at reducing excessive alcohol consumption in an app [40].

#### Phase 2: Design and translation

The aims when building the Action Planning module were as follows: to (i) make setting action plans as easy as possible and (ii) help participants understand why they should set an action plan in the first place.

The main screen of the Action Planning module briefly explained the benefits of setting an action plan and provided an example of one. Users could create action plans and were provided with numerous examples. Users could review and edit the action plans they had set and were given details about the benefits of setting action plans and evidence to support their effectiveness.

### Identity change

Identity change is the principle of adopting an identity that is incongruent with an undesired behavior—in this case excessive alcohol consumption. The module’s aim was to help users separate alcohol consumption from their sense of identity.

#### Phase 1: Rationale for selection

Excessive drinking is central to many peoples’ sense of self, particularly students [82]. The relationship between identity and behavior change has not been investigated in the field of alcohol research, though there is evidence from the smoking cessation literature that identity change may be an effective intervention technique [83–85]. The PRIME theory of motivation proposes that identity is a source of motives, self-regulation, and stability of behavior [86]. Identity was also identified in a consensus approach as a theoretical domain to explain behavior change [32].

#### Phase 2: Design and translation

This module (named “Drink + Me”) aimed to help users foster a change in their identity so that they did not see being a “drinker” as a key part of their identity. The main menu screen explained the general purpose of the module and listed its three strategies: (i) “Flipsides of drinking,” (ii) “Memos,” and (iii) “I am….”

“Flipsides of drinking” provided pairs of alcohol-related outcome expectancies: each pair consisted of a positive expectancy (or benefit) and a negative “flipside” (or cost) that are important in influencing drinking behavior [87]. For example, “*Drinking helps me think better*” was paired with “*My decision making is impaired and I spend far more money than I intended*.” This section aimed to highlight both the pros and cons of excessive drinking and reframe positive effects with their corresponding potential negative. Ten pairs of examples were provided, collated from different studies [88] and scales [89], and users were encouraged to enter their own flipsides to make the section more personal and salient.

“Memos” allowed users to record video messages to watch at a later date and to set reminders to either watch these memos or record additional ones. The app suggested users record memos at different times, such as whilst sober, during drinking or after drinking, with different messages to themselves. Users could set a reminder to record or watch these memos at the most salient times.

“I am…” aimed to get users to identify the values of importance to their identity or sense of self in line with Self-affirmation Theory [90] and then consider whether their behavior after excessive drinking was inconsistent with those values. Users were asked to list their personal “values of importance” [91] or select some from a list of examples based on values most commonly used in different studies [92] and considered of greatest relevance to the Drink Less app. Users were then prompted to consider which of these values they struggled to reconcile when drinking too much. The section ended with examples of common “values of importance” to people, and possible ways in which someone’s behavior could be inconsistent with those values. On subsequent uses of the “I am…” section, users were given the choice of reviewing their previous entry or completing the section again.

## REGISTRATION AND HELP SECTIONS

All users had to complete the Registration section on opening the app for the first time, which consisted of five screens. Users were given details about the study and those who consented to participate then completed the AUDIT and a sociodemographic assessment that measured as follows: gender, age, ethnicity, educational level, employment status, smoking status, and country. Users were also asked for their reason for using the app (“interested in drinking less” or “just browsing”) and to provide their email address (an inclusion criterion for the subsequent trial).

The “Help” section contained information about UK drinking guidelines, the harms of drinking, good goal setting, and advice for users who think that they might have a serious problem with their drinking. Users could change the time they received the reminder to complete their drinking diary or turn this reminder off or back on. An “About the app” section included information on the team behind the app, their contact details, references for information used in the app, the participant information sheet, details of the privacy policy, a link to opt-out of the trial, and a link to rate the app.

## DISCUSSION

### Summary of main findings

*Drink Less* is a standalone app-based intervention aimed at individuals in the UK who drink excessively who are seeking digital support for alcohol reduction. It appears to be the first alcohol reduction app that has been systematically developed based on evidence and theory and using a person-based approach. The initial development resulted in a smartphone app centered around alcohol reduction goal setting with five independent intervention modules: (i) Normative Feedback, (ii) Cognitive Bias Retraining, (iii) Self-monitoring and Feedback, (iv) Action Planning, and (v) Identity Change. Early prototypes were refined to make the app easy, rewarding, and beneficial to use. The modular app was developed with a rigorous evaluation strategy in place to identify the optimum combination of intervention components to reduce excessive alcohol consumption, which underpins intervention optimization according to the Multiphase Optimization Strategy.

### Comparison with prior work

Although most apps for alcohol reduction targeted at the general population have not been formally evaluated [93], three apps for specific alcohol problems or populations have been examined in randomized control trials [94]. The “Promillekoll” and “PartyPlanner” apps targeted risky alcohol use in Swedish university students, though neither had a statistically significant positive effect on reducing alcohol use [95]. “A-CHESS” (Addiction Comprehensive Health Enhancement Support System) supported individuals in the USA with recovery from alcohol use disorders and was found to result in significantly fewer risky drinking days than in participants who only received treatment as usual [25]. However, the target population of the Drink Less app (general population of individuals who drink excessively in the UK) is substantially different from the target population for A-CHESS.

### Strengths and limitations

*Drink Less* appears to be the first alcohol reduction app aimed at the general population of individuals who drink excessively to have reported a systematic and transparent approach to its development. The intervention components for *Drink Less* were selected on the best available empirical and theoretical evidence, which included an expert consensus study. However, the final decision for inclusion drew on researcher judgement where there was no direct evidence for the effectiveness of specific intervention components in an alcohol reduction app. This process was still preferable to most alcohol reduction apps, which do not report the use of any evidence or theory during the intervention development [14].

There are multiple ways to conduct the translation of intervention components to app modules and if a different team of researchers and app developers conducted the same process, it is possible that they would have resulted in a different final app. However, the translation process was conducted in close collaboration with experienced app developers and based on design principles from previous studies. Therefore, it is likely that the intervention components have been implemented in *Drink Less* in one of the best possible ways to create a user friendly app.

The development of *Drink Less* is in accordance with the Open Science principles of making materials, data, results, and publications freely available [23]. As well as the reporting of the intervention development and modules, which may provide a helpful template to other researchers developing health-related behavior change apps, the source code for the app will be shared on request with other researchers who wish to develop similar apps. This is important for efficient scientific progress as well as reducing development costs for other researchers.

There is no clear existing evidence base to work from as app interventions for reducing excessive drinking are a recent field of research. Therefore, it is important to use triangulation—different approaches to address the same underlying question—with evidence from multiple sources when developing a new intervention [96]. The triangulation of findings is a strength of this development process and whilst each method has its own advantages and disadvantages, increased confidence can be given to intervention components that arise from multiple sources.

The app went through a number of iterations before being released, a process which accords with Medical Research Council guidance on complex behavior change interventions [27] and the Multiphase Optimization Strategy [97]. Changes made to the app resulted from testing by researchers and a formal usability study [46]. It was deemed important to take time to develop a user friendly and engaging app as behavior change interventions need to engage users in order to be effective [98]. However, this involves a longer development process and could increase the likelihood of the app becoming obsolete, as digital technology already advances faster than the speed at which interventions are typically developed and evaluated [99]. To resolve this conflict, digital intervention developers are encouraged to iterate rapidly, releasing, testing, and improving new versions until one suitable for experimentation is found. Due to time constraints, usability testing was only conducted with the initial version of the app, not the final one.

### Future research

*Drink Less* was developed with intervention components designed in independent modules with two versions—“enhanced” (hypothesized active ingredients for reducing alcohol consumption) and “minimal” (control)—to facilitate optimization according to the Multiphase Optimization Strategy. The independent and interactive effects of the intervention modules at reducing excessive alcohol consumption have been investigated in an initial factorial randomized control trial [100]. The factorial evaluation is a crucial first step for optimizing the intervention—by informing decisions whether to then include or remove individual intervention components—before evaluating the app as a single multicomponent package in a full randomized control trial [27, 28, 97]. The factorial randomized control trial of *Drink Less* could inform and provide useful information for the development of future digital behavior change interventions, as well as future versions of the app.

The app was launched in May 2016 with an active dissemination strategy that involved promotion through relevant organizations (e.g., Public Health England, Cancer Research, UK) and listing in the iTunes Store according to best practices for app store optimization (e.g., careful selection of keywords, a well-written description and illustrative screenshots). The app now consistently appears in the top three of results for “alcohol” searches in the UK App Store and there have been approximately 17,000 unique downloads in the UK since its launch.

*Drink Less* is currently only available for UK users and on iPhone operating systems. The decision to make the app UK-specific was based on the differences in the standard definition of units between countries, which would require additional country-specific coding. A single delivery platform was chosen as we considered it important to focus resources on developing a native app that would provide a better user experience. If effective, *Drink Less* can be taken as a proof of concept, developed on Android and released worldwide, to reach a larger proportion of users. There is no evidence currently for whether the app will generalize to people outside of the UK, though there are a number of parts to the app (e.g., units, country-specific guidelines, and country-specific alcohol consumption comparisons) that would need to be adapted before the app could be used elsewhere. However, as *Drink Less* has been developed in accordance with the Open Science principles, and as its code is available to researchers on request, there is the potential for other researchers to build further on the app by extending the reach to other geographic regions and, potentially, specific target populations.

## CONCLUSIONS

*Drink Less* is the first alcohol reduction app to our knowledge that has been systematically developed based on evidence and theory, using a person-based approach. *Drink Less* is centered around a goal setting intervention module and has five experimental modules: Normative Feedback, Cognitive Bias Retraining, Self-monitoring and Feedback, Action Planning, and Identity Change. *Drink Less* has been developed with a rigorous evaluation strategy in place, to identify the optimum combination of intervention components to help individuals who drink excessively to reduce their consumption of alcohol. A well-designed and effective intervention to reduce excessive alcohol consumption would have important implications for public health. The development of *Drink Less* is in line with the Open Science principles, to support the efficient optimization of future interventions and reduce trial wastage.

## Compliance with Ethical Standards

**Conflicts of Interest:** J.B. has received unrestricted research funding from Pfizer related to smoking cessation. R.W. has received research funding and undertaken consultancy for companies that manufacture smoking cessation medications. R.W. and S.M. are advisers to the National Centre for Smoking Cessation and Training. S.M. is Director of the UCL Centre for Behaviour Change.

**Primary Data:** This manuscript is original, has not been published, and is not currently submitted elsewhere. No primary data is reported in this article that describes the development process of an intervention; the authors have full control of the reported intervention and agree to allow the journal to review all source code if requested.

**Ethical Approval:** This article does not contain any studies with human participants or animals performed by any of the authors.

**Informed Consent:** This article has no human participants and therefore informed consent and IRB approval was not required.

**Authors’ Contributions:** All authors have seen and approved this paper. We have read and followed the Instructions for Authors.

## Supplementary Material

Supplementary Table 1

Supplementary File 2

## References

[1] World Health Organisation. Global Status Report on Alcohol and Health. Luxembourg: WHO; 2014.

[2] Balakrishnan R, Allender S, Scarborough P, Webster P, Rayner M The burden of alcohol-related ill health in the United Kingdom. J Public Health (Oxf). 2009;31(3):366–373. 19493915 10.1093/pubmed/fdp051

[3] Ezzati M, Lopez AD, Rodgers A, Vander Hoorn S, Murray CJ; Comparative Risk Assessment Collaborating Group Selected major risk factors and global and regional burden of disease. Lancet. 2002;360(9343):1347–1360. 12423980 10.1016/S0140-6736(02)11403-6

[4] Lim SS, Vos T, Flaxman AD, et al A comparative risk assessment of burden of disease and injury attributable to 67 risk factors and risk factor clusters in 21 regions, 1990-2010: a systematic analysis for the Global Burden of Disease Study 2010. Lancet. 2012;380(9859):2224–2260. 23245609 10.1016/S0140-6736(12)61766-8 PMC4156511

[5] Babor T, Higgins J, Saunders J, Monteiro M. AUDIT: The Alcohol use Disorders Identification Test Guidelines for use in Primary Care, 2nd ed. Geneva, Switzerland: World Health Organisation; 2001. 1–40 p.

[6] Rehm J, Mathers C, Popova S, Thavorncharoensap M, Teerawattananon Y, Patra J Global burden of disease and injury and economic cost attributable to alcohol use and alcohol-use disorders. Lancet. 2009;373(9682):2223–2233. 19560604 10.1016/S0140-6736(09)60746-7

[7] Kaner EF, Beyer FR, Brown J, et al Personalised digital interventions for reducing hazardous and harmful alcohol consumption in community-dwelling populations (Protocol). Cochrane Database Syst Rev. 2015;(1):1–14. 10.1002/14651858.CD011479.pub2 PMC6483779 28944453

[8] West R, Michie S. A Guide to Development and Evaluation of Digital Interventions in Healthcare. London: Silverback Publishing; 2016.

[9] Pew Research Center: Internet & Technology. Mobile fact sheet [Internet] 2017 Available at http://www.pewinternet.org/fact-sheet/mobile/. Accessibility verified July 20, 2017.

[10] Ofcom. The Communications Market Report. Ofcom: London; 2016.

[11] Google. Our Mobile Planet: United Kingdom. Google Services: London; 2013;1–39.

[12] Tecmark. Smartphone Usage Statistics 2014—UK Survey of Smartphone Users [Internet]. 2014. Available at https://www.tecmark.co.uk/smartphone-usage-data-uk-2014/. Accessibility verified June 4, 2017.

[13] Naughton F, Hopewell S, Lathia N, et al A context-sensing mobile phone app (Q sense) for smoking cessation: a mixed-methods study. JMIR Mhealth Uhealth. 2016;4(3):e106. 27637405 10.2196/mhealth.5787 PMC5045522

[14] Crane D, Garnett C, Brown J, West R, Michie S Behavior change techniques in popular alcohol reduction apps: content analysis. J Med Internet Res. 2015;17(5):e118. 25977135 10.2196/jmir.4060 PMC4468601

[15] Weaver ER, Horyniak DR, Jenkinson R, Dietze P, Lim MS “Let’s get Wasted!” and other apps: characteristics, acceptability, and use of alcohol-related smartphone applications. JMIR Mhealth Uhealth. 2013;1(1):e9. 25100681 10.2196/mhealth.2709 PMC4114432

[16] Penzenstadler L, Chatton A, Van Singer M, Khazaal Y Quality of smartphone apps related to alcohol use disorder. Eur Addict Res. 2016;22(6):329–338. 27598779 10.1159/000449097

[17] Witkiewitz K, Desai SA, Bowen S, Leigh BC, Kirouac M, Larimer ME Development and evaluation of a mobile intervention for heavy drinking and smoking among college students. Psychol Addict Behav. 2014;28(3):639–650. 25000269 10.1037/a0034747 PMC6143292

[18] Brendryen H, Johansen A, Nesvåg S, Kok G, Duckert F Constructing a theory- and evidence-based treatment rationale for complex ehealth interventions: development of an online alcohol intervention using an intervention mapping approach. JMIR Res Protoc. 2013;2(1):e6. 23612478 10.2196/resprot.2371 PMC3629462

[19] Linke S, McCambridge J, Khadjesari Z, Wallace P, Murray E Development of a psychologically enhanced interactive online intervention for hazardous drinking. Alcohol. 2008;43(6):669–674. 10.1093/alcalc/agn066 PMC2570848 18693217

[20] Dulin PL, Gonzalez VM, King DK, Giroux D, Bacon S Development of a smartphone-based, self-administered intervention system for alcohol use disorders. Alcohol Treat Q. 2013;31(3):321–36. 10.1080/07347324.2013.800425 PMC3857707 24347811

[21] Michie S, Brown J, Geraghty AW, et al Development of stopadvisor: a theory-based interactive internet-based smoking cessation intervention. Transl Behav Med. 2012;2(3):263–275. 24073123 10.1007/s13142-012-0135-6 PMC3717907

[22] Tombor I, Shahab L, Brown J, Crane D, Michie S, West R Development of smokefree baby: a smoking cessation smartphone app for pregnant smokers. Transl Behav Med. 2016;6(4):533–545. 27699682 10.1007/s13142-016-0438-0 PMC5110502

[23] Munafò MR Opening up addiction science. Addiction. 2016;111(3):387–388. 26455433 10.1111/add.13147

[24] Glasziou P, Altman DG, Bossuyt P, et al Reducing waste from incomplete or unusable reports of biomedical research. Lancet. 2014;383(9913):267–276. 24411647 10.1016/S0140-6736(13)62228-X

[25] Hoffmann TC, Oxman AD, Ioannidis JP, et al Enhancing the usability of systematic reviews by improving the consideration and description of interventions. BMJ. 2017 July 20;358:j2998. 28729459 10.1136/bmj.j2998

[26] Brown J, Michie S, Geraghty AW, et al Internet-based intervention for smoking cessation (StopAdvisor) in people with low and high socioeconomic status: a randomised controlled trial. Lancet Respir Med. 2014;2(12):997–1006. 25262458 10.1016/S2213-2600(14)70195-X

[27] Craig P, Dieppe P, Macintyre S, Michie S, Nazareth I, Petticrew M; Medical Research Council Guidance Developing and evaluating complex interventions: the new medical research council guidance. BMJ. 2008 September 29;337:a1655. 18824488 10.1136/bmj.a1655 PMC2769032

[28] Collins LM, Murphy SA, Nair VN, Strecher VJ A strategy for optimizing and evaluating behavioral interventions. Ann Behav Med. 2005;30(1):65–73. 16097907 10.1207/s15324796abm3001_8

[29] Michie S, van Stralen MM, West R The behaviour change wheel: a new method for characterising and designing behaviour change interventions. Implement Sci. 2011;6 (1):42. 21513547 10.1186/1748-5908-6-42 PMC3096582

[30] Michie S, Atkins L, West R. The Behaviour Change Wheel - A Guide To Designing Interventions. 1st ed. London: Silverback Publishing; 2014.

[31] Cane J, O’Connor D, Michie S Validation of the theoretical domains framework for use in behaviour change and implementation research. Implement Sci. 2012;7 (1):37. 22530986 10.1186/1748-5908-7-37 PMC3483008

[32] Michie S, Johnston M, Abraham C, Lawton R, Parker D, Walker A; “Psychological Theory” Group Making psychological theory useful for implementing evidence based practice: a consensus approach. Qual Saf Health Care. 2005;14(1):26–33. 15692000 10.1136/qshc.2004.011155 PMC1743963

[33] Collins LM, Baker TB, Mermelstein RJ, et al The multiphase optimization strategy for engineering effective tobacco use interventions. Ann Behav Med. 2011;41(2):208–226. 21132416 10.1007/s12160-010-9253-x PMC3053423

[34] Michie S, Richardson M, Johnston M, et al The behavior change technique taxonomy (v1) of 93 hierarchically clustered techniques: building an international consensus for the reporting of behavior change interventions. Ann Behav Med. 2013;46(1):81–95. 23512568 10.1007/s12160-013-9486-6

[35] Michie S, Johnston M, Carey R Behaviour change techniques. In: Gellman M, ed. Encyclopedia of Behavioral Medicine. New York: Springer; 2016: 1580–1581.

[36] Yardley L, Morrison L, Bradbury K, Muller I The person-based approach to intervention development: application to digital health-related behavior change interventions. J Med Internet Res. 2015;17(1):e30. 25639757 10.2196/jmir.4055 PMC4327440

[37] O’Brien HL The influence of hedonic and utilitarian motivations on user engagement: the case of online shopping experiences. Interact Comput. 2010;22(5):344–352.

[38] Bargas-Avila JA, Hornbæk K Old wine in new bottles or novel challenges: a critical analysis of empirical studies of user experience. Paper presented at: SIGCHI Conference on Human Factors in Computing Systems (CHI’11) 2011: 2689–2698.

[39] Michie S, Whittington C, Hamoudi Z, Zarnani F, Tober G, West R Identification of behaviour change techniques to reduce excessive alcohol consumption. Addiction. 2012;107(8):1431–1440. 22340523 10.1111/j.1360-0443.2012.03845.x

[40] Garnett C, Crane D, West R, Brown J, Michie S Identification of behavior change techniques and engagement strategies to design a smartphone app to reduce alcohol consumption using a formal consensus method. JMIR Mhealth Uhealth. 2015;3(2):e73. 26123578 10.2196/mhealth.3895 PMC4526967

[41] Michie S, West R, Campbell R, Brown J, Gainforth H. ABC of Behaviour Change Theories. Great Britain: Silverback Publishing; 2014.

[42] Perski O, Blandford A, West R, Michie S Conceptualising engagement with digital behaviour change interventions: a systematic review using principles from critical interpretive synthesis. Transl Behav Med. 2016;7(2):254–267. 10.1007/s13142-016-0453-1 PMC5526809 27966189

[43] Ubhi HK, Michie S, Kotz D, van Schayck OCP, Selladurai A, West R Characterising smoking cessation smartphone applications in terms of behaviour change techniques, engagement and ease-of-use features. Transl Behav Med. 2015;6(3):410–417. 10.1007/s13142-015-0352-x PMC4987605 27528530

[44] Fowler M, Highsmith J, Beck K, et al The agile manifesto. Software Development. 2001;9(8):28–35.

[45] Yardley L, Ainsworth B, Arden-Close E, Muller I The person-based approach to enhancing the acceptability and feasibility of interventions. Pilot Feasibility Stud. 2015;1 (1):37. 27965815 10.1186/s40814-015-0033-z PMC5153673

[46] Crane D, Garnett C, Brown J, West R, Michie S Factors influencing usability of a smartphone app to reduce excessive alcohol consumption: think aloud and interview studies. Front Public Health. 2017;5(39):1–19. 28421175 10.3389/fpubh.2017.00039 PMC5376568

[47] Schunk DH. Self-Regulation through Goal Setting. ERIC Clearinghouse on Counseling and Student Service. Greensboro: University of North Carolina at Greensboro; 2001.

[48] Locke EA, Latham GP. A Theory of Goal Setting & Task Performance. New Jersey: Prentice-Hall, Inc; 1990.

[49] Moskowitz GB, Grant H. The Psychology of Goals. New York: Guilford Press; 2009.

[50] Kaner EF, Beyer FR, Garnett C, et al Personalised digital interventions for reducing hazardous and harmful alcohol consumption in community-dwelling populations. Cochrane Database Syst Rev. 2017;9:CD011479. 28944453 10.1002/14651858.CD011479.pub2 PMC6483779

[51] Department of Health. UK Chief Medical Officers’ Alcohol Guidelines Review. Summary of the Proposed New Guidelines. London: Department of Health and Social Care; 2016.

[52] Royal College of Physicians. RCP Comments on the Frequency of Alcohol Consumption [Internet]. 2011. https://www.rcplondon.ac.uk/news/rcp-comments-frequency-alcohol-consumption. Accessibility verified February 10, 2018.

[53] Garnett C, Crane D, West R, Michie S, Brown J, Winstock A Normative misperceptions about alcohol use in the general population of drinkers: a cross-sectional survey. Addict Behav. 2015 March 1;42:203–206. 25482365 10.1016/j.addbeh.2014.11.010 PMC4294420

[54] Cunningham JA, Neighbors C, Wild TC, Humphreys K Normative misperceptions about alcohol use in a general population sample of problem drinkers from a large metropolitan city. Alcohol Alcohol. 2012;47(1):63–66. 22028458 10.1093/alcalc/agr125 PMC3243438

[55] Neighbors C, Larimer ME, Lewis MA Targeting misperceptions of descriptive drinking norms: efficacy of a computer-delivered personalized normative feedback intervention. J Consult Clin Psychol. 2004;72(3):434–447. 15279527 10.1037/0022-006X.72.3.434

[56] Elster J Social norms and economic theory. In: Crothers L, Lockhart C, eds. Culture and Politics. New York: Palgrave Macmillan; 2000.

[57] Miller DT, McFarland C When social comparison goes awry: the case of pluralistic ignorance. In: Suls J, Wills TA, eds. Social Comparison: Contemporary Theory and Research. Hillsdale, New Jersey: Lawrence Erlbaum Associates Inc; 1991; 287–313.

[58] Beard E, Brown J, West R, et al Protocol for a national monthly survey of alcohol use in England with 6-month follow-up: ‘the Alcohol Toolkit Study’. BMC Public Health. 2015;15 (1):230. 25884652 10.1186/s12889-015-1542-7 PMC4363185

[59] Rooke SE, Hine DW, Thorsteinsson EB Implicit cognition and substance use: a meta-analysis. Addict Behav. 2008;33(10):1314–1328. 18640788 10.1016/j.addbeh.2008.06.009

[60] Bechara A Decision making, impulse control and loss of willpower to resist drugs: a neurocognitive perspective. Nat Neurosci. 2005;8(11):1458–1463. 16251988 10.1038/nn1584

[61] Strack F, Deutsch R Reflective and impulsive determinants of social behavior. Pers Soc Psychol Rev. 2004;8(3):220–247. 15454347 10.1207/s15327957pspr0803_1

[62] Eberl C, Wiers RW, Pawelczack S, Rinck M, Becker ES, Lindenmeyer J Approach bias modification in alcohol dependence: do clinical effects replicate and for whom does it work best?Dev Cogn Neurosci. 2013 April 1;4:38–51. 23218805 10.1016/j.dcn.2012.11.002 PMC6987692

[63] Wiers RW, Rinck M, Kordts R, Houben K, Strack F Retraining automatic action-tendencies to approach alcohol in hazardous drinkers. Addiction. 2010;105(2):279–287. 20078486 10.1111/j.1360-0443.2009.02775.x

[64] Wiers RW, Eberl C, Rinck M, Becker ES, Lindenmeyer J Retraining automatic action tendencies changes alcoholic patients’ approach bias for alcohol and improves treatment outcome. Psychol Sci. 2011;22(4):490–497. 21389338 10.1177/0956797611400615

[65] Pronk T, van Deursen DS, Beraha EM, Larsen H, Wiers RW Validation of the amsterdam beverage picture set: a controlled picture set for cognitive bias measurement and modification paradigms. Alcohol Clin Exp Res. 2015;39(10):2047–2055. 26431117 10.1111/acer.12853 PMC5054858

[66] van Deursen DS, Salemink E, Smit F, Kramer J, Wiers RW Web-based cognitive bias modification for problem drinkers: protocol of a randomised controlled trial with a 2x2x2 factorial design. BMC Public Health. 2013;13 (1):674. 23870532 10.1186/1471-2458-13-674 PMC3723484

[67] Lister C, West JH, Cannon B, Sax T, Brodegard D Just a fad? Gamification in health and fitness apps. JMIR Serious Games. 2014;2(2):e9. 25654660 10.2196/games.3413 PMC4307823

[68] Kanfer FH Self-monitoring: methodological limitations and clinical applications. J Consult Clin Psychol. 1970;35(2):148–52.

[69] Carver CS, Scheier MF Control theory: a useful conceptual framework for personality-social, clinical, and health psychology. Psychol Bull. 1982;92(1):111–135. 7134324

[70] National Institute for Health and Care Excellence. Behaviour Change: Individual Approaches. London: NICE; 2014.

[71] Gaume J, McCambridge J, Bertholet N, Daeppen JB Mechanisms of action of brief alcohol interventions remain largely unknown—a narrative review. Front Psychiatry. 2014 August 26;5:108. 25206342 10.3389/fpsyt.2014.00108 PMC4143721

[72] Jansen JP Self-monitoring of glucose in type 2 diabetes mellitus: a Bayesian meta-analysis of direct and indirect comparisons. Curr Med Res Opin. 2006;22(4):671–681. 16684428 10.1185/030079906X96308

[73] Burke LE, Conroy MB, Sereika SM, et al The effect of electronic self-monitoring on weight loss and dietary intake: a randomized behavioral weight loss trial. Obesity (Silver Spring). 2011;19(2):338–344. 20847736 10.1038/oby.2010.208 PMC3268702

[74] Krebs P, Duncan DT Health app use among US mobile phone owners: a national survey. JMIR Mhealth Uhealth. 2015;3(4):e101. 26537656 10.2196/mhealth.4924 PMC4704953

[75] Milward J, Khadjesari Z, Fincham-Campbell S, Deluca P, Watson R, Drummond C User preferences for content, features, and style for an app to reduce harmful drinking in young adults: analysis of user feedback in app stores and focus group interviews. JMIR Mhealth Uhealth. 2016;4(2):e47. 27220371 10.2196/mhealth.5242 PMC4897297

[76] Harkin B, Webb TL, Chang BP, et al Does monitoring goal progress promote goal attainment? A meta-analysis of the experimental evidence. Psychol Bull. 2016;142(2):198–229. 26479070 10.1037/bul0000025

[77] Sobell LC, Sobell MB Timeline follow-back. In: Litten R, Allen J, eds. Measuring Alcohol Consumption. Totowa, NJ: Humana Press; 1992: 41–72.

[78] Gollwitzer PM, Sheeran P Implementation intentions and goal achievement: a meta‐analysis of effects and processes. Pers Soc Psychol Bull. 2006; 38:69–119.

[79] Hagger MS, Lonsdale A, Koka A, et al An intervention to reduce alcohol consumption in undergraduate students using implementation intentions and mental simulations: a cross-national study. Int J Behav Med. 2012;19(1):82–96. 21562782 10.1007/s12529-011-9163-8

[80] Palfai T Automatic processes in self-regulation: implications for alcohol interventions. Cogn Behav Pract. 2004;11(2):190–201.

[81] Armitage CJ Effectiveness of experimenter-provided and self-generated implementation intentions to reduce alcohol consumption in a sample of the general population: a randomized exploratory trial. Health Psychol. 2009;28(5):545–553. 19751080 10.1037/a0015984

[82] Piacentini MG, Banister EN Getting hammered? ...students coping with alcohol. J Consum Behav. 2006;5 (2):145–56.

[83] West R, Walia A, Hyder N, Shahab L, Michie S Behavior change techniques used by the English stop smoking services and their associations with short-term quit outcomes. Nicotine Tob Res. 2010;12(7):742–747. 20478957 10.1093/ntr/ntq074

[84] Tombor I, Shahab L, Brown J, West R Positive smoker identity as a barrier to quitting smoking: findings from a national survey of smokers in England. Drug Alcohol Depend. 2013;133(2):740–745. 24075070 10.1016/j.drugalcdep.2013.09.001

[85] Tombor I, Shahab L, Herbec A, Neale J, Michie S, West R Smoker identity and its potential role in young adults’ smoking behavior: a meta-ethnography. Health Psychol. 2015;34(10):992–1003. 25622078 10.1037/hea0000191 PMC4577249

[86] West R, Brown J. Theory of Addiction. 2nd ed. Chichester, West Sussex: John Wiley & Sons; 2013. 280 p.

[87] Adams SL, McNeil DW Negative alcohol expectancies reconsidered. Psychol Addict Behav. 1991;5(1):9–14.

[88] Leigh BC, Stacy AW Alcohol expectancies and drinking in different age groups. Addiction. 2004;99(2):215–227. 14756714 10.1111/j.1360-0443.2003.00641.x

[89] Leigh BC, Stacy AW Alcohol outcome expectancies: scale construction and predictive utility in higher order confirmatory models. Psychol Assess. 1993;5(2):216–29.

[90] Steele CM The psychology of self-affirmation: sustaining the integrity of the self. In: Berkowitz L, ed. Advances in Experimental Social Psychology. New York: Academic Press; 1988: 261–302.

[91] Epton T, Harris PR Self-affirmation promotes health behavior change. Health Psychol. 2008;27(6):746–752. 19025270 10.1037/0278-6133.27.6.746

[92] MCQueen A, Klein WMP Experimental manipulations of self-affirmation: a systematic review. Self Identity. 2006;5(4):289–354.

[93] Cohn AM, Hunter-Reel D, Hagman BT, Mitchell J Promoting behavior change from alcohol use through mobile technology: the future of ecological momentary assessment. Alcohol Clin Exp Res. 2011;35(12):2209–2215. 21689119 10.1111/j.1530-0277.2011.01571.x PMC3221771

[94] Meredith SE, Alessi SM, Petry NM Smartphone applications to reduce alcohol consumption and help patients with alcohol use disorder: a state-of-the-art review. Adv Health Care Technol. 2015;1:47–54. 27478863 10.2147/AHCT.S65791 PMC4963021

[95] Gajecki M, Berman AH, Sinadinovic K, Rosendahl I, Andersson C Mobile phone brief intervention applications for risky alcohol use among university students: a randomized controlled study. Addict Sci Clin Pract. 2014;9 (1):11. 24985342 10.1186/1940-0640-9-11 PMC4091647

[96] Munafò MR, Davey Smith G Robust research needs many lines of evidence. Nature. 2018;553(7689):399–401. 32094809 10.1038/d41586-018-01023-3

[97] Collins LM, Murphy SA, Strecher V The multiphase optimization strategy (MOST) and the sequential multiple assignment randomized trial (SMART): new methods for more potent eHealth interventions. Am J Prev Med. 2007;32(5 Suppl):S112–S118. 17466815 10.1016/j.amepre.2007.01.022 PMC2062525

[98] Yardley L, Spring BJ, Riper H, et al Understanding and promoting effective engagement with digital behavior change interventions. Am J Prev Med. 2016;51(5):833–842. 27745683 10.1016/j.amepre.2016.06.015

[99] Schueller SM, Munoz RF, Mohr DC Realizing the potential of behavioral intervention technologies. Curr Dir Psychol Sci. 2013;22(6): 478–83.

[100] Garnett C, Crane D, Michie S, West R, Brown J Evaluating the effectiveness of a smartphone app to reduce excessive alcohol consumption: protocol for a factorial randomised control trial. BMC Public Health. 2016;16 (1):536. 27392430 10.1186/s12889-016-3140-8 PMC4939028

