# Supplementary material for: The development of Drink Less: an alcohol reduction smartphone app for excessive drinkers
Source: Transl Behav Med. 2018 May 4;9(2):296–307. doi: 10.1093/tbm/iby043 (PMC6417151; doi:10.1093/tbm/iby043)
Supplement: Supplementary Table 1 [file iby043_suppl_supplementary_table_1.docx]

# Supplementary Table 1: Details of the *Drink Less* modules

| Module | Objectives/Key features | Potential process of change  (COM-B; TDF) | Rationale for selection | Details of module | | BCTs (‘enhanced’ version) | BCTs (‘minimal’ version) |
| --- | --- | --- | --- | --- | --- | --- | --- |
|  |  |  |  | Enhanced | Minimal |  |  |
| Goal setting | Allow users to set different weekly goals and provide information on setting appropriately specific and difficult goals. | Capability (psychological)  Motivation (reflective) | Wide body of evidence for its effectiveness  Identified by experts  Increases engagement with other modules | Set weekly goals for alcohol units, alcohol-free days, calories from alcohol, spend on alcohol | No minimal version of this module, Goal Setting was given to all users | 1.1 Goal setting (behaviour) | N/A |
| Normative Feedback | Inform users of the social drinking norm and alert them to any discrepancy with how they believe their drinking compares with normal to how it actually compares with normal.  Feedback on how drinking actually compares; feedback on how they think drinking compares | Capability (psychological); knowledge  Motivation (automatic) | Social Norms theory  Identified by experts  Existence of normative misperceptions  Empirical evidence of effectiveness in interventions (face-to-face & digital) | Questions assessing how users think they compare with others.  Infographics illustrating how user’s drinking actually compares with other adults and others of same gender and age. | Text on risks of drinking too much (from PHE website) | 2.7 Feedback on outcomes of behaviour  6.2. Social comparison | 5.1 Information about health consequences  5.6 Information about emotional consequences |
| Cognitive Bias Re-training | Use a form of cognitive bias modification to strengthen cognitive control over the automatic biases to approach alcohol that predict alcohol use that exist amongst the users through an engaging game. | Motivation (automatic); reinforcement | PRIME theory  Dual Process models of addiction  Identified by experts | Game with all alcohol related pictures associated with “avoid” and all soft drink pictures associated with “approach”.  Additional section of text on why and how this sort of game is believed to work. | Game with 50% of alcohol related pictures associated with “avoid” and 50% associated with “approach”. Same for soft drink pictures. | No individual BCTs are directly relatable to this intervention module. In combination, the following are of relevance:  7.8 Associative learning  8.1 Behavioural practice/rehearsal  8.3 Habit formation | None |
| Self-monitoring and Feedback | Facilitate easy and on-going recording of alcohol consumption; provide feedback on consumption, consequences of consumption and progress against goals.  Allow users to monitor their consumption; provides feedback on consumption; feedback on consequences of consumption (mood, productivity, sleep) | Motivation (reflective) | Control Theory  Proven effective in health behaviour change  Identified by experts | Ability to record drinks, graph showing units consumed calories consumed, amount spent on alcohol. Record mood, productivity, clarity, sleep quality, graph illustrating how they differ on mornings after heavy drinking compared to mornings after light/no drinking. Feedback on progress towards goals: cumulatively as the week progresses, on the past week and on all previous weeks. | Ability to record drinks, single graph showing units consumed. No other self-monitoring facilitated or feedback provided | 1.5 Review behaviour goals 1.6 Discrepancy between current behaviour and goal 2.2 Feedback on behaviour 2.3 Self-monitoring of behaviour 2.4 Self-monitoring of outcomes of behaviour 2.7 Feedback on outcomes of behaviour 5.2 Salience of consequences 5.6 Information about emotional consequences 10.3 Non-specific reward 10.4 Social reward 10.9 Self-reward | 2.3 Self-monitoring of behaviour |
| Action Planning | Allow users to create implementation intentions for dealing with difficult drinking situations.  Allow users to create/review action plans; provides examples of action plans; rationale for creating action plans | Motivation (reflective)  Opportunity (social) | Control theory  Effective in health behaviour change  Identified by experts | Create implementation intentions, review implementation intentions already created, gain understanding of why to set implementation intentions. | Gain understanding of why to set implementation intentions only. | 1.4 Action planning  9.1 Credible source | None |
| Identity Change | Help users foster a change in their identity so that users do not see themselves as “drinkers” as a key part of their identity, which should aid their behaviour change attempt. | Motivation (reflective); social/professional role and identity, optimism | PRIME theory  Association between alcohol-identity and alcohol consumption  Limited empirical study of effectiveness in alcohol interventions  Empirical evidence of effectiveness in smoking cessation interventions | Memos – record messages about drinking or when drunk too much to watch in the future  “I am”: identifying and considering those values that are important to you, and whether you do not live up to those values when you have drunk too much.  Flipsides of drinking:  Providing examples of the negative sides of positive alcohol expectancies. | Text on how identity is an important factor in how we behave and advise to think about the undesired consequences of drinking too much. | 5.1 Information about health consequences  5.2 Salience of consequences  5.3 Information about social and environmental consequences  5.5 Anticipated regret  5.6 Information about emotional consequences  9.2 Pros and cons  13.2 Framing/reframing  13.3 Incompatible beliefs  13.4. Valued self-identity  13.5. Identity associated with changed behaviour | None |
