# Supplementary material for: The development of Drink Less: an alcohol reduction smartphone app for excessive drinkers
Source: Transl Behav Med. 2018 May 4;9(2):296–307. doi: 10.1093/tbm/iby043 (PMC6417151; doi:10.1093/tbm/iby043)
Supplement: Supplementary File 2 [file iby043_suppl_supplementary_file_2.docx]

Supplementary File 2 - Description of the intervention

Table of Contents

[Intervention modules 4](#_Toc490489145)

[1.1 Goal setting 4](#_Toc490489146)

[1.1.1 Set and view goals 4](#_Toc490489147)

[1.1.2 Set a new goal 4](#_Toc490489148)

[1.1.3 How to set good goals 5](#_Toc490489149)

[1.1.4 Behaviour change techniques 5](#_Toc490489150)

[1.2 Normative Feedback 5](#_Toc490489151)

[1.2.1 Assessment of normative misperceptions 5](#_Toc490489152)

[1.2.2 Providing normative feedback 6](#_Toc490489153)

[1.2.3 Behaviour change techniques 7](#_Toc490489154)

[1.2.4 Experimental vs control group features 7](#_Toc490489155)

[1.3 Cognitive Bias Re-training 8](#_Toc490489156)

[1.3.1 Play the game 8](#_Toc490489157)

[1.3.2 Instructions 9](#_Toc490489158)

[1.3.3 Previous scores 10](#_Toc490489159)

[1.3.4 How re-training your mind works 10](#_Toc490489160)

[1.3.5 Behaviour change techniques 11](#_Toc490489161)

[1.3.6 Experimental vs control group features 11](#_Toc490489162)

[1.4 Self-monitoring and Feedback 11](#_Toc490489163)

[1.4.1 Recording drinks 11](#_Toc490489164)

[1.4.2 The Mood Diary 13](#_Toc490489165)

[1.4.3 Feedback 14](#_Toc490489166)

[1.4.4 Dashboard 15](#_Toc490489167)

[1.4.5 Goal feedback 16](#_Toc490489168)

[1.4.6 Feedback about current week’s goal(s) 17](#_Toc490489169)

[1.4.7 Your hangover and you (feedback from the Mood Diary) 17](#_Toc490489170)

[1.4.8 Calendar 17](#_Toc490489171)

[1.4.9 Add drinks panel 18](#_Toc490489172)

[1.4.10 What has and hasn’t worked 18](#_Toc490489173)

[1.4.11 Behaviour change techniques 18](#_Toc490489174)

[1.4.12 Experimental vs control group features 19](#_Toc490489175)

[1.5 Action Planning 19](#_Toc490489176)

[1.5.1 Create an action plan 20](#_Toc490489177)

[1.5.2 Your action plans 20](#_Toc490489178)

[1.5.3 Why set an action plan 20](#_Toc490489179)

[1.5.4 Behaviour change techniques 20](#_Toc490489180)

[1.5.5 Experimental vs control group features 21](#_Toc490489181)

[1.6 Identity Change 21](#_Toc490489182)

[1.6.1 Flipsides of Drinking 21](#_Toc490489183)

[1.6.2 Memos 22](#_Toc490489184)

[1.6.3 ‘I am…’ 23](#_Toc490489185)

[1.6.4 Behaviour change techniques 24](#_Toc490489186)

[1.6.5 Experimental vs control group features 24](#_Toc490489187)

[Registration and Help sections 25](#_Toc490489188)

[1.7 Registration 25](#_Toc490489189)

[1.8 Help section 25](#_Toc490489190)

[1.8.1 Information about alcohol 26](#_Toc490489191)

[1.8.2 App settings 26](#_Toc490489192)

[1.8.3 Information about the app 26](#_Toc490489193)

[1.8.4 Behaviour change techniques 27](#_Toc490489194)

[The build process 27](#_Toc490489195)

[1.9 Choosing a developer and platform 27](#_Toc490489196)

[1.10 Testing and iterating 28](#_Toc490489197)

[1.11 How decisions about what to include were made 29](#_Toc490489198)

[Changes made in response to the usability study 30](#_Toc490489199)

[1.12 Determining which changes to make 30](#_Toc490489200)

[1.13 Normative Feedback module 31](#_Toc490489201)

[1.14 Cognitive Bias Re-training module 32](#_Toc490489202)

[1.15 Self-monitoring and Feedback module 32](#_Toc490489203)

[1.16 Action Planning module 34](#_Toc490489204)

[1.17 Identity Change module 34](#_Toc490489205)

[1.18 Goal setting module 35](#_Toc490489206)

[1.19 Other elements of the app 35](#_Toc490489207)

[1.20 Follow-up 36](#_Toc490489208)

[Screenshots 38](#_Toc490489209)

[1.21 Intervention modules 38](#_Toc490489210)

[1.21.1 Goal setting 38](#_Toc490489211)

[1.21.2 Normative Feedback 40](#_Toc490489212)

[1.21.3 Cognitive Bias Re-training 45](#_Toc490489213)

[1.21.4 Self-monitoring and Feedback 49](#_Toc490489214)

[1.21.5 Action Plans 61](#_Toc490489215)

[1.21.6 Identity Change (Drink + Me) 65](#_Toc490489216)

[1.22 Other features 71](#_Toc490489217)

[1.22.1 Progress screen 71](#_Toc490489218)

[1.22.2 Registration 72](#_Toc490489219)

[1.23 Apple App Store listing 74](#_Toc490489220)

[1.24 Feedback given on AUDIT score 75](#_Toc490489221)

[1.25 User-selectable options for alcoholic drinks 76](#_Toc490489222)

[1.26 Goal feedback 77](#_Toc490489223)

[1.27 References 79](#_Toc490489224)

# Intervention modules

## Goal setting

The aims when building the goal setting module were to offer participants a selection of goals, help them determine what a suitable difficult, specific and proximal goal might be and make the process of setting goals as easy as possible.

On launching the goal setting module (accessed via ‘Create and view goals’ on the ‘Progress’ screen of the app, Figure 1.22.1), participants were presented with two links to other screens (‘Set and view goals’; and ‘How to set good goals’) and the question “I want to drink less because…” (Figure 1.21.1, a). This question was intended to help participants establish an overarching reason for reducing their alcohol consumption; responses were prominently displayed on the Dashboard, at the top of the ‘Your active goals’ area (Figure 1.21.4.4).

### Set and view goals

‘Set and view goals’ contained a list of all active goals and any previously set goals (goals that had either been deleted or whose end date had passed). Text briefly explained some of the principles of good goal setting and a prominent button at the bottom of the screen allowed participants to set a new goal (Figure 1.21.1, b). An information button provided more help on using this screen (Figure 1.21.1, c).

### Set a new goal

Participants could choose between four types of goal: units, spending, calories and alcohol free days (Figure 1.21.1, d). Whilst a participant could set a goal to any level they wanted, default options provided guidance about potentially suitable goals. The default unit goal was 14 (which reflects UK government guidelines for weekly alcohol consumption for both men and women [1]), the default calorie goal was 1,100 (which equates to the approximate calorie equivalent of 14 units of average strength beer or wine) and the default number of alcohol free days was 3 (following recommendations by the Royal College of Physicians [2] and UK Government guidelines [1]). No default was set for spending because the price of alcoholic drinks varies considerably throughout the UK. Goals were automatically set to recur every Monday but participants could deselect this option if they wished. If a unit goal was chosen participants could click a link to see the number of units in typical drinks (Figure 1.21.1, e). On tapping the ‘Save’ button a pleasing sound was played and confirmation message appeared to let participants know they had successfully set a goal (Figure 1.21.1, f). An information button provided more help on using this screen.

### How to set good goals

‘How to set good goals’ contained brief information about good goal setting, a list of the types of goal that could be set and a suggestion of the next steps participants should take.

### Behaviour change techniques

| BCT | App Location | Section |
| --- | --- | --- |
| 1.1 Goal setting (behaviour) | Set and view goals | 1.1.1 |

## Normative Feedback

Normative Feedback was included as part of the initial registration module in a tunnelled approach. Users could subsequently review their normative feedback and their AUDIT responses and results via ‘Review your drinking’ in the Progress screen (Figure 1.21.2.4, a, b & c).

### Assessment of normative misperceptions

Users’ normative misperceptions were assessed with two questions: ‘How do you think your drinking compares with others in the UK?’ and ‘How do you think your drinking compares with other [women/men] aged [16-24/25-34/35-44/45-54/55+]?’ (Figure 1.21.1, a). Users provided their answer using a dial mechanism by tapping on the screen or dragging the red needle on the gauge. Users could select any position on the gauge (which ranged from 0-100) though responses were grouped into nine categories: very low; low; low-average; average (middle 20%); high-average; high; very-high; top 10%. Beneath the gauge the relevant response option appeared.

### Providing normative feedback

A screen of text attempting to prepare the user for the normative feedback with information about the source of the data was shown before providing the normative feedback (Figure 1.21.1, b). This was done in response to the usability study, which found that the majority of users found their normative feedback surprising, reacted defensively, and questioned the validity of the information provided [3]. After this screen, users were provided with normative feedback and feedback on how this differed from their normative misperception. Normative feedback was then provided in the form of the user’s percentile in a distribution of population drinking levels in England (ranking within a population is more effective than comparing users with an average [4]) and what that meant for their alcohol-related risk relative to others. This normative feedback was delivered in two visual representations: the same dial mechanism (a ‘gauge’) and a ‘people infographic’ (Figures 1.21.2.2, a & b). Two separate visual representations of this data were chosen to increase the dose of the normative feedback provided.

Users were compared against: i) the general population in the UK (Figure 1.21.2.2, a & b) and ii) a sub-group of their gender and age group, to increase the salience of the information, (Figure 1.21.2.3, a & b) and iii) only drinkers in the general population and iv) only drinkers in the sub-group of their gender and age group. The inclusion of a comparison against drinkers-only arose from the usability study, which found that some users disregarded normative feedback as they found that comparison unreliable [3]. The data used as the comparison were based on alcohol use of a representative sample of the general population in England derived from the Alcohol Toolkit Study (ATS) between March 2014 and October 2015 [5].

Visual representations of the users’ reported drinking levels and their normative misperceptions were used to minimise the text and make the screen more aesthetically pleasing. Below the visual representations there were short sentences describing the visual representation.
*UK gauge: Your drinking is greater than x% of other [people/drinkers] in the UK. You [over-/under-/correctly] estimated how much other people in the UK drink* (Figure 1.21.2.2, a)*.
UK people infographic: This means for every 20 [people/drinkers] in the UK you’re at a greater alcohol-related risk than x of them* (Figure 1.21.2.2, b)*.
Age/gender gauge: Your drinking is greater than x% of other [women/men] aged [16-24/25-34/35-44/45-54/55+] [ _ / who drink]. You [over-/under-/correctly] estimated how much other [women/men] [ _ / who drink] aged [16-24/25-34/35-44/45-54/55+] consume* (Figure 1.21.2.3, a). *Age/gender people infographic: This means for every 20 [women/men] aged [16-24/25-34/35-44/45-54/55+] [ _ / who drink] you’re at a greater alcohol-related risk than x of them* (Figure 1.21.2.3, b)*.*

A screen of text was shown after the normative feedback that aimed to increase the users’ self-efficacy by reassuring them that it was common to find the results surprising and that the *Drink Less* app could help reduce their drinking (Figure 1.21.1, c).

### Behaviour change techniques

| BCT | App Location | Section |
| --- | --- | --- |
| 2.7 Feedback on outcomes of behaviour | Normative Feedback | 1.2.2 |
| 6.2 Social comparison | Normative Feedback | 1.2.2 |

### Experimental vs control group features

Participants assigned to the experimental group and given the enhanced version of the module were provided with all of the content described above: questions assessing their normative misperceptions and normative feedback shown using a gauge and people infographic.

Participants assigned to the control group and given the minimal version of the module were still asked the questions assessing their normative misperceptions but did not receive any normative feedback. Instead, brief advice in plain text was provided, as this was the usual control in similar interventions (Figure 1.21.2.4.1). This text came from the ‘Brief Advice Tool’ (2010) on the Public Health England website and was as follows:
*Drinking too much can put you at an increased risk of a number of things including…
Memory loss
Relationship problems
Depression
Impotence
Injury
High blood pressure
Liver disease
Cancer
Weight gain*

Any user who had an AUDIT score below average received a separate, non-experimental module and were provided with social norms information emphasising that light drinking is normal. This was done to avoid the possible ‘boomerang effect’ [6] of below average drinkers increasing their drinking to reach the social norm. Social norms information have been found to have a better preventative effect on alcohol consumption amongst light and non-drinking students than personalised normative feedback [7].

## Cognitive Bias Re-training

The cognitive bias re-training module was a game called “Yes Please, No Thanks” and used approach avoidance training (AAT) in an attempt to re-train biases to alcohol cues from an ‘approach’ to an ‘avoid’ bias [8]. The main screen had links to “Play the game”, “Instructions”, “Previous scores” and “How re-training your mind works” (Figure 1.21.3.1, a). The main screen also had a sentence to emphasise the aim of the game: “This game aims to help you re-train your mind so you get more used to saying “No thanks” to alcohol when you choose to.”

### Play the game

A total of 40 images were used in the game (20 alcohol-related and 20 non-alcohol-related), selected from the validated Amsterdam Beverage Picture Set [9]. All the alcohol images were in the format associated with ‘avoid’ (“No thanks”) and non-alcohol images in the format associated with ‘approach’ (“Yes please”) [10]. There was a sentence at the top of the screen recapping the instructions for the game (Figure 1.21.3.3, a).

Each game lasted one minute, which was considered the appropriate length of time during informal user testing. There was a countdown from 60 to 0 on the screen indicating how much time was left for each game (Figure 1.21.3.3, a). Users approached or avoided the images using their finger to swipe the screen and move the image (swiping up the screen for ‘avoid’ and down the screen for ‘approach’). A zooming effect occurred as the image was moved either up or down, whereby the image increased or decreased in size to generate a stronger sense of approach or avoidance [8,10] (Figure 1.21.3.3, a). Visual and auditory feedback was provided after each response; when a user responded correctly, the screen flashed green and a ‘correct’ sound was played, if an incorrect response occurred, the screen flashed red and a sound indicating an error was played. After the game finished, users were told their score and reminded to ‘play again’ to see if they could improve on their score (Figure 1.21.3.3, b & c).

### Instructions

Instructions about whether users should ‘approach’ or ‘avoid’ images were given based on the image format (landscape vs. portrait) (Figure 1.21.3.3, a) [11] and users were randomly counterbalanced when they first played the game [10]. Graphics were included to illustrate how the game worked, as participants in the usability study did not find the instructions clear [3] (Figure 1.21.3.2 b & c). The text for the instruction screens were as follows:
*i) You will see pictures in either landscape (short and wide) or portrait (long and think). The key thing to remember is that it’s the shape of the box, not the image itself, which matters.
When you see a {landscape} picture use your finger to pull it towards you (i.e. down). This is like saying “Yes please”. When you see a {portrait} picture use your finger to push it away from you (i.e. up). This is like saying “No, thanks”.
You will have 60 seconds to see how many pictures you can sort. Quick is good but remember, the quicker you go, the more errors you might make. You get a point for every one you get right but lose two if you get it wrong.
ii) Say “No thanks” to [landscape] pictures by pushing them away from you.
iii) Say “Yes please” to [portrait] pictures by pulling them towards you.
iv) Play now!*

### Previous scores

Previous scores were illustrated using a bar graph to create a sense of competition (a principle of gamification [12]) and encourage users to keep playing in an attempt to improve their score, and enhance engagement with the intervention module (Figure 1.21.3.3, c). Each correct response scored ‘+1’ and each incorrect response scored ‘-2’ to incentivise attempting to respond correctly rather than randomly.

### How re-training your mind works

A section on “how re-training your mind works” was included as participants in the usability study wanted to know why and how the game might help them reduce their consumption of alcohol, and liked the idea of “re-training your mind” when it was explained to them [3]. This screen consisted of plain, non-scientific text that proposed how the game might be effective (Figure 1.21.3.1, b) and was as follows:

Research has shown that we sometimes drink but don’t know why this is. This is like an unconscious impulse to say “yes” to the idea of drinking. One way to help you stick to a decision to drink less is to reduce the power of that impulse, and a number of studies have shown this to work.
Because some of the pictures involve alcohol, some studies have found that it could retrain your mind unconsciously to reject alcohol – at least a little bit!
This game has been designed to help you do just that, and be a bit of fun too. If you’d like to read more about the theories and evidence showing how this works, please visit Help > References.

A similar sentence was placed on the Game main screen to emphasise its purpose (Figure 1.21.3.1, a).

### Behaviour change techniques

No individual BCTs from the BCT Taxonomy version 1 are directly relatable to this intervention module.

### Experimental vs control group features

Participants assigned to the experimental group and given the enhanced version of the module were provided with all of the content described above.

Participants assigned to the control group and given the minimal version of the module had access to: Play the game, instructions, and previous scores (Figure 1.21.3.4, a). The main menu screen did not make any reference to how the game might help the user reduce their alcohol consumption. The text above the options read: “*Read the instructions below or start playing now and pick it up as you go.”*

The game in the minimal version involved different contingencies to those in the intensive version. Half of the ‘avoid’ trials had alcohol images and the other half had non-alcohol images. For the ‘approach’ trials, half had alcohol images and the other half had non-alcohol images. In the same way as in the intensive version, users with the minimal version were counterbalanced in terms of the orientation of the images and the approach/avoid instructions [10].

## Self-monitoring and Feedback

### Recording drinks

Self-monitoring is the most commonly included BCT in alcohol reduction apps available in the UK [13], and is a feature considered highly important to users of a web-based alcohol intervention [14], users of an alcohol use disorder app [15], users of alcohol reduction apps [16] and users of the *Drink Less* app. A well-implemented self-monitoring module is, therefore, important for meeting user needs and may help the app be chosen in preference to others available.

The main aim when building the self-monitoring module was to make the process of recording drinks as easy as possible. Large numbers of users stop using health apps if they find data entry too burdensome [17], users of alcohol reduction apps often criticise the process of recording drinks [16] and interventions which greatly increased the frequency of self-monitoring have been found to produce small-to-medium-sized improvements in goal attainment [18]. Simplifying the process of entering drinks may lead to greater use of this module and a corresponding increase in intervention effectiveness.

The self-monitoring module was made easy to use in the following ways. The link for adding drinks was made larger than other links and placed in the centre of the navigation tab bar, indicating its importance to participants and making it easy to find (Figure 1.21.4.1). Tapping this link allowed participants to choose from six categories of drinks (beer, cider, wine, fortified wine, spirits, or alcopops, Figure 1.21.4.1). Once a drink category had been selected, participants were presented with a screen allowing them to enter more details about the drink, such as its ABV (alcohol by volume), size, quantity and price (Figure 1.21.4.2, b. A full list of the type of drinks and options available for each can be found in section 1.25).

A participant could accept the default entries and click the Save button without entering other information. This reduced the process of adding drinks to the minimum number of steps, whilst allowing participants to add more detail should they wish. Reducing steps required default options to be carefully selected in order to minimise the likelihood of inaccurate recording. The default type of drink (e.g. for beer: lager, for wine: red) reflected the most popular type of that drink in the UK [19]. There does not appear to be data about the most common volume of an alcoholic drink, defaults were therefore chosen on the volume users may be most familiar with (e.g. for beer: pint) or that reflect the median volume available in pubs or restaurants (e.g. for wine: a 175ml glass). The default ABV was calculated from a mean of the ABV from popular drinks of that type, rounded up to the nearest integer (the decision to round up rather than down was made in order to overestimate rather than underestimate the number of units an individual had consumed). No default price was set, as prices for alcoholic drinks vary widely throughout the UK.

Use of the self-monitoring module was made easier with the addition of links for ‘Regulars’ and ‘Recent’ (Figure 1.21.4.1). Regulars contains a list of saved configurations of type, ABV, size and price of a drink. Recent contains all the drinks the participant has previously entered, sorted by date, most recent first. Tapping any of the drinks on either of these screens restores the previous configuration of type, ABV, size and price. These configurations can either be edited or saved.

Participants were reminded to complete a log of their drinking at 11:05am each morning, though the reminder time could be changed. The default was set for about eleven o’clock in order not to disturb late risers and to allow participants time to complete their morning routine. It was set for just past the hour in order not to conflict with other reminders set for that time. Setting reminders to just past the hour is an approach taken by the popular diet and fitness logging app, MyFitnessPal. Reminders took the form of an on-screen alert (Figure 1.21.4.6, a), a ‘Badge App icon’ (Apple’s name for the red dot which appears on an app’s icon on a participant’s home screen, Figure 1.21.4.6, b) and a prompt on the participant’s dashboard of the app (Figure 1.21.4.6, c).

### The Mood Diary

In addition to self-monitoring consumption, participants were also encouraged to monitor the consequences of consumption by way of a Mood Diary that asked them to rate their mood, productivity, clarity and sleep quality on a scale of 0-10 on a daily basis (Figure 1.21.4.7, a). Scores on mornings after nights of heavy drinking were compared with scores on mornings after nights of light or no drinking, and displayed in graphs (section 1.4.7, below).

The potential effectiveness of increasing understanding about the consequences of alcohol consumption is supported by the PRIME Theory of motivation. PRIME Theory posits that behaviour is determined on a moment-to-moment basis by whichever competing impulse or inhibition is strongest at the time, and that enacting a new behaviour requires activating wants and needs strong enough to overcome competing impulses or inhibitions [20,21]. Enacting a new alcohol behaviour in the moment can be challenging because alcohol increases impulsivity, impairs inhibitory control and increases attentional bias for alcohol-related cues [22–25]. It was reasoned that increasing salience about the consequences of consumption on next-day mood, productivity, clarity and sleep quality, may reduce impulsive attitudes towards alcohol consumption and increase the motivation not to drink.

The four consequences of consumption were chosen on the basis of evidence. The residual effects of alcohol can impair productivity, increase absenteeism and affect self-reported feelings of ability to perform [26–29]. Alcohol can impair next-day academic performance [30], pilot performance [31] and concentration [32]. It can affect next-day mood states [33], increase self-reported anxiety, irritability and depression [34,35] and results in a poorer sense of overall well-being [36]. The effects of alcohol on sleep are well-documented. Whilst alcohol is a sedative and can induce the rapid onset of sleep [37], the sleep that ensues is negatively affected. Alcohol can disturb the second half of the night sleep [38], resulting in reduced sleep, increased light sleep and frequent awakenings [30,34,39]. Alcohol can have extensive effects on daytime sleepiness [39] and may result in post-consumptive daytime impairment [37].

At the end of the Mood Diary questions were two additional questions. The first asked participants if they had any other drinks to record and displayed the date of their last recorded entry to prompt recollection of drinking behaviour since that point (Figure 1.21.4.7, b). The second asked participants if they drank more than they wanted to yesterday. If participants answered ‘No’ they were subsequently asked “What helped you achieve your goal?” If they answered ‘Yes’ they were asked “What got in the way?” (Figure 1.21.4.7, b). Responses to these questions were displayed in the ‘What worked and didn’t work’ screen of the app (described in section 1.4.10 below).

### Feedback

There were two main aims for the feedback element of this module: 1) provide feedback about consumption and the consequences of consumption (calorie intake, spend on alcohol, and how mood, productivity, clarity and sleep quality are affected by heavy drinking); and 2) provide feedback about progress toward goal(s), celebrate successes, and avoid using language that might be interpreted as judgemental or discouraging. In addition, audible or visual feedback given after participant actions (for example after recording a drink or setting a goal), was intended to provide a sense of accomplishment and positive reinforcement that may encourage repeated use of the app.

The second aim for the feedback module was intended to prevent goal disengagement. The challenge with interventions that facilitate goal setting is to encourage participants to set goals that are difficult but attainable. Difficult but attainable goals result in high levels of performance [40], goals that appear to be unattainable can result in goal abandonment [41–44]. In order to help users set appropriate goals, the app provided positive reinforcement when goals were met and motivational feedback when goals were missed. If goals were missed by a substantial amount twice in a row, feedback suggested that the goal may presently be too difficult as to be attainable and that the participant might want to change their goal to one that is slightly easier. If goals were exceeded by a substantial amount twice in a row, feedback suggested that the goal may be too easy as to be rewarding and that the participant might want to make their goal more difficult. Feedback in both cases was presented as advisory rather than directional and was only delivered after goals were missed twice in a row in order to account for periods of unusual drinking activity such as celebrations or fasts, and the text shown to participants asked them to consider whether this was an unusual period before changing their goal (Figure 1.21.4.9, a). When tapped, this text took participants to the goal setting screen, where information on good goal setting was again presented. Full details of the goal feedback provided can be found in section 1.26.

A ‘substantial amount’ was selected as > 20% above or below the target goal. I.e. if a participant set a goal of 20 units a week and drank 24 units or above for two weeks in a row they would receive feedback suggesting the goal may be too difficult. If they set the same goal and drank 16 units or less for two weeks in a row they would receive feedback suggesting the goal may be too easy. The 20% figure was chosen as it allows for mildly discrepant goal performances and so may promote continued goal striving, whilst encouraging participants to address the more substantial discrepancies that may result in goal abandonment.

### Dashboard

The dashboard contained a number of feedback elements. The units graph displayed the amount of alcohol consumed per week since the app was downloaded (Figure 1.21.4.3), with a horizontal line to indicate the position on the y-axis of a participant-set unit goal (if a participant had not set a unit goal the line was placed at 14 units to reflect UK government guidelines [1]). Each bar on the graph could be tapped to display a summary of the drinks consumed that week (e.g. 3 x glasses of red wine, 2 x pints of beer, 2 x whiskeys), accompanied by totals of the number of alcohol free days, units consumed, amount spent on and calories consumed from alcohol; and how those figures compared with the previous week (Figure 1.21.4.5, b). The calorie tab displayed the total number of calories consumed from alcohol during the current week, how that figure compared to the previous week and the total number of calories consumed since the app was downloaded (Figure 1.21.4.5, a). The money tab showed the same information as the calorie screen but in terms of spend on alcohol.

### Goal feedback

The dashboard provided a link to feedback that demonstrated how the participant had performed against their goal(s) to date; and brief summary feedback about how they were performing against their goal(s) in the current week (Figure 1.21.4.4, all goals ran from Monday to Sunday). Feedback about how the participant had performed against the goal to date consisted of three screens: Last week, Hit Rate and Success Rate.

#### Last week

The ‘Last week’ screen provided feedback about goal progress for the last complete week. Information displayed was: the goal title (e.g. “Goal: Have at least 3 alcohol free days a week”), the period covered (e.g. “Last ended: 28 November 2016”), the number of units consumed, calories consumed, alcohol free days or spend on alcohol (e.g. “Alcohol free days; 5”) an icon to reflect whether a participant had exceeded (green circle), hit (green tick), nearly hit (orange circle) or missed (red cross) the goal. Below the icon, text feedback that tallied with the participant’s success or otherwise was given (e.g. “Congratulations on a great week of achievement. Feel proud? You should.” Figure 1.21.4.9, a). Section 1.26 contains full details of all goal feedback provided.

#### Hit rate

The ‘Hit Rate’ screen displayed feedback in a bar chart that detailed success against the goal to date, one bar for each week. A horizontal line indicated the participant’s target and text below the chart summarised their success so far (e.g. “You’ve hit 50% of your goal to drink less than 19 units a week”, Figure 1.21.4.9, b).

#### Success rate

The ‘Success Rate’ screen provided feedback about how frequently a goal had been exceeded, hit, nearly hit or missed and displayed the information in pie chart form. Text below the chart informed participants how many times in a row they had hit their goal (Figure 1.21.4.9, c).

### Feedback about current week’s goal(s)

Feedback about performance against the goal in the week to date was displayed on the dashboard in the ‘Your active goals’ area and consisted of the goal title, information about current level of progress against goal(s) and length of time before the goal ends (e.g. “Drink less than 19 units a week; So far 8 units, ends in 3 days”, Figure 1.21.4.4). For participants who had recently downloaded the app and not completed a whole week, the dashboard showed how long before goal feedback would be given (i.e. how long until the next Monday).

### Your hangover and you (feedback from the Mood Diary)

‘Your hangover and you’ contained four graphs which compared a participant’s mood, productivity, clarity and sleep quality on days after heavy drinking with days after light or no drinking (Figure 1.21.4.8, b). Heavy drinking days were defined as more than six units per day for men or women, which reflects UK government guidelines [1]. Scores for this graph were taken from entries a participant had made to the Mood Diary questions (described in section 1.4.2, above).

### Calendar

The calendar displayed dates in month form, beneath which were bars coloured green to provide feedback about a no drinking day, orange for a light drinking day (greater than zero units but less than six units), red for heavy drinking day (more than six units), grey for no record entered. The calendar also showed the total number of alcohol free days since the app had been downloaded (Figure 1.21.4.10, a).

Each day of the calendar could be tapped to see a list of drinks recorded for that day. The daily record showed details for each drink (its name, size, number of units and calories) as well as the total number of units consumed from all drinks (Figure 1.21.4.11, a).

Days where a drinking record had not been entered were marked in grey on the calendar. Tapping these days displayed text that adhered to the Timeline Follow-back procedure and prompted participants to look at their diary, text messages or emails to jog their memory of their drinking for that day [45] (Figure 1.21.4.10, b). If participants tapped the Alcohol Free Day button a pleasing sound and animation played, with a large green tick and “Keep up the good work!” displayed (Figure 1.21.4.10, c).

### Add drinks panel

An alcohol free day could also be recorded on the main Add Drinks screen. When tapped, a pleasing sound and animation played and a tick and “Keep up the good work!” was displayed, similar to the positive feedback given when an alcohol free day was recorded on the calendar (Figure 1.21.4.2, a).

### What has and hasn’t worked

The ‘What has and hasn’t worked’ screen displayed entries to the question in the Mood Diary that asked “Did you drink more than you wanted to yesterday?” (described in section 1.4.2, above). Responses were listed in the respective part of the ‘What has and hasn’t worked’ screen (Figure 1.21.4.8, c). This screen was intended to help participants remind themselves of the behaviour that had promoted or prevented goal attainment.

### Behaviour change techniques

| BCT | App Location | Section |
| --- | --- | --- |
| 1.5 Review behaviour goals | Dashboard/ Goal feedback | 1.4.4/  1.4.5 |
| 1.6 Discrepancy between current behaviour and goal | Dashboard/  Goal feedback/  Calendar | 1.4.4/  1.4.5/  1.4.8 |
| 2.2 Feedback on behaviour | Dashboard/  Goal feedback | 1.4.4/  1.4.5 |
| 2.3 Self-monitoring of behaviour | Recording drinks | 1.4.1 |
| 2.4 Self-monitoring of outcomes of behaviour | Mood Diary | 1.4.2 |
| 2.7 Feedback on outcomes of behaviour | Your hangover and you | 1.4.7 |
| 5.2 Salience of consequences | Dashboard | 1.4.4 |
| 5.6 Information about emotional consequences | Mood Diary | 1.4.2 |
| 10.3 Non-specific reward | Recording drinks confirmation | 1.4.1 |
| 10.4 Social reward | Dashboard/  Calendar | 1.4.4/  1.4.8 |
| 10.9 Self-reward | Goal feedback | 1.4.5 |

### Experimental vs control group features

Participants assigned to the experimental group and given the enhanced version of the module were prompted to complete a log of their alcohol consumption and answer the Mood Diary questions on a daily basis. They were also given all the feedback above.

Participants assigned to the control group and given the minimal version of the module were only asked to complete a log of their alcohol consumption, they were not presented with the Mood Diary questions (Figure 1.21.4.12, c). Participants assigned to the control group were not given any feedback: their dashboard did not display any graphs, there was only very brief information about goal progress and they were given no feedback about goal performance (Figure 1.21.4.12, a). The information button on the Dashboard that displayed more information about the dashboard and Mood Diary contained different text for participants in each group (Figure 1.21.4.12, b).

## Action Planning

The two aims when building the Action Planning module were: 1) make setting action plans as easy as possible, and 2) help participants understand why they should set an action plan in the first place. ‘Action plan’ was the term used within the app, in place of the more accurate, but potentially less well-understood, ‘implementation intentions’.

On launching the Action Planning module (accessed via ‘Create and View action plans’ on the ‘Progress’ screen of the app, Figure 1.21.5.1, a), participants were presented with information that briefly explained the benefits of setting an action plan and provided an example of one. The If/Then elements of an action plan were distinguished graphically to help establish that an action plan needed to include both a cue and a response (Figure 1.21.5.1, b). Also on this screen were three links to different action plan-related content: ‘Create an action plan’; ‘Your action plans’; and ‘Why set an action plan?’ (Figure 1.21.5.1, b). An information button provided help on using the screen.

### Create an action plan

‘Create an action plan’ contained a field for each of the ‘If’ and ‘Then’ components of an action plan, text within each field reminded participants what content should go in each (Figure 1.21.5.2, a). If a participant attempted to save an action plan with one or other of the fields empty, an error message directed them to the part that needed completing (Figure 1.21.5.2, a). An information button provided numerous examples of action plans (Figure 1.21.5.2, b).

### Your action plans

‘Your action plans’ provided a list of all the action plans a participant had set (Figure 1.21.5.3, a). The ‘If’ of each action plan was displayed in list form and could be tapped to display its associated ‘Then’. The display of the ‘Then’ was subtly animated in order to provide a more enjoyable user experience.

### Why set an action plan

‘Why set an action plan’ contained more details about the benefits of setting action plans, examples of action plans and evidence to support their effectiveness (Figure 1.21.5.1, c).

### Behaviour change techniques

| BCT | App Location | Section |
| --- | --- | --- |
| 1.4 Action planning | Create an action plan/ Your action plans | 1.5.1/  1.5.2 |
| 9.1 Credible source | Why set an action plan | 1.5.3 |

### Experimental vs control group features

Participants assigned to the experimental group and given the enhanced version of the module were given access to all three action plan screens, detailed above. Participants assigned to the control group and given the minimal version of the module were only given access to a single screen with basic text information about action plans (Figure 1.21.5.4, accessed via ‘Create and View action plans’ on the ‘Progress’ screen of the app).

## Identity Change

This module aimed to help users foster a change in their identity so that users did not see being a ‘drinker’ as a key part of their identity. This module was named “Drink + Me” as participants in the usability study found the name “Identity” confusing and expected a section based on their user profile. The main menu screen explained the general purpose of the module and listed its three strategies: i) Flipsides of Drinking, ii) Memos, and iii) ‘I am…’ (Figure 1.21.6.1).

### Flipsides of Drinking

Flipsides of Drinking provided pairs of alcohol-related outcome expectancies: each pair consisted of a positive expectancy (or benefit) and a negative ‘flipside’ (or cost), that are important in influencing drinking behaviour [46]. This section aimed to highlight both the pros and cons of excessive drinking and reframe positive effects with their potential negative. The first screen of this section included a brief introduction to the Flipsides of drinking (Figure 1.21.6.2, a). Ten pairs of examples were provided, collated from different studies [47] and scales [48,49] (Figure 1.21.6.2, b), and users were encouraged to enter their own flipsides to make the section more personal and salient (Figure 1.21.6.2, c).

The ten pairs of examples are listed below:

1. Feel more confident / I’m more likely to become argumentative and aggressive, and be involved in a fight
2. Drinking helps me forget problems at work or school / I can become depressed
3. Drinking makes socialising easier / I need my friends to look after me and take me home, spoiling their evening
4. Alcoholic drinks taste good / I can have a whole days’ worth of calories just from drinks and feel too rubbish the following day to go out and exercise
5. Drinking makes me feel more romantic / I might have sex with someone who I wouldn’t want to if sober
6. Drinking helps me think better / My decision making is impaired and I spend far more money than I intended
7. Drinking helps calm me down when I’m angry / Too much means I can become aggressive and argumentative, and get into fights
8. Drinking is a nice way to celebrate special occasions / I might have memory lapses and forget the occasion
9. Drinking gives me more confidence in myself / I say something inappropriate that ends up offending someone
10. Feel great and have fun / I feel awful (groggy and tired) the following day

### Memos

Memos allowed users to record salient video messages to watch at a later date, and to set reminders to either record or watch these memos (Figure 1.21.6.3, a & b). The app suggested users record memos at different times, such as whilst sober, during drinking or after drinking with different messages to themselves (Figure 1.21.6.3, a). For example, the ‘after drinking’ memo could be recorded the morning after a night out as a salient reminder of the negative consequences of excessive drinking and potentially to induce ‘*anticipated regret’* before their next drinking event. Users could set a reminder to record or watch these memos at the most salient times (Figure 1.21.6.3, b). Users could change the name of any memo from its default (“[date] [time]”).

The text in this section was as follows:
This is where you can record messages to yourself to watch in the future. Maybe try saying a tongue twister after you’ve had some drinks or a message that will persuade you not to drink more than you planned to.

Here you can set reminders to record memos. It may be helpful to set reminders to record a memo when you’re out drinking or the morning after a heavy night. You can also remind yourself to watch one you’ve already recorded. Maybe at a time you think you’re likely to start drinking.

### ‘I am…’

‘I am…’ aimed to get users to identify the values of importance to their identity or sense of self and consider whether their behaviour after excessive drinking was inconsistent with those values. This section was based around Self-affirmation Theory, which postulates that focusing on ‘values of importance’ makes an individual less defensive to threatening information [50]. The purpose of this section was briefly explained to users (Figure 1.21.6.4, a) before they were prompted to use their own photo (for personalisation purposes) or use a default image of a smiley face (Figure 1.21.6.4, b). Users were asked to list their personal ‘values of importance’ [51,52] or select some from a list of examples [53] (Figure 1.21.6.4, c). The examples were based on those most commonly used in different studies [54] and considered of greatest relevance to the *Drink Less* app. These examples were: honest; good friend; responsible; health conscious; friendly; and fun.

The user was prompted to consider which of these values they struggled to reconcile when drinking too much and then the users’ photo or default image appeared with these values surrounding it (Figure 1.21.6.5, a & b). The section ended with examples of common ‘values of importance’ to people, and possible ways in which someone’s behaviour could be inconsistent with those values (Figure 1.21.6.5, c). The text for these examples was as follows:
Here are some of our examples of values that don’t go with getting drunk. Obviously they won’t all apply to you though…
Honest -> Exaggerate stories for ‘comic’ effect but take it too far
Good friend -> End up spoiling my friends’ night as they need to take care of me once I’ve had too much to drink
Responsible -> Spend more money than planned and go over your weekly budget
Health conscious -> After drinking too much I often have an unhealthy snack, like a kebab, on my way home
Friendly -> I can get quite argumentative once I have an excessive amount of alcohol
Fun -> End up half asleep, unable to join in with what’s going on

On subsequent uses of the ‘I am…’ section, users were given the choice of reviewing their previous entry or completing the section again.

### Behaviour change techniques

| BCT | App Location | Section |
| --- | --- | --- |
| 5.1 Information about health consequences | Flipsides of Drinking | 1.6.1 |
| 5.2 Salience of consequences | Memos | 1.6.2 |
| 5.3 Information about social and environmental consequences | Flipsides of Drinking | 1.6.1 |
| 5.5 Anticipated regret | Memos | 1.6.2 |
| 5.6 Information about emotional consequences | Flipsides of Drinking | 1.6.1 |
| 9.2 Pros and cons | Flipsides of Drinking | 1.6.1 |
| 13.2 Framing/reframing | Flipsides of Drinking | 1.6.1 |
| 13.3 Incompatible beliefs | ‘I am…’ | 1.6.3 |
| 13.4 Valued self-identity | ‘I am…’ | 1.6.3 |
| 13.5 Identity associated with changed behaviour | ‘Drink + Me’ main page ‘I am…’ | 1.6  1.6.3 |

### Experimental vs control group features

Participants assigned to the experimental group and given the enhanced version of the module were provided with all of the content described above. Participants assigned to the control group and given the minimal version of the module were provided with simple text describing the role of identity in behaviour change and maintenance, though nothing to aid the user in fostering an identity change (Figure 1.21.6.6, a). The text for the minimal version was as follows:

You are here because you’ve decided that you want to drink less. Now, take a moment to imagine yourself as this person who drinks less. What would it mean for you?
Building up a new identity as someone who does not drink excessively is an important part of drinking less. Sometimes the consequences of drinking too much are not what you intended or wanted to happen. It can be helpful to think about these negative consequences of drinking too much when you’re trying to drink less.

# Registration and Help sections

## Registration

On opening the app for the first time users were presented with a welcome screen with text reminding them of its experimental nature (the description on the Apple App Store also contained information that the app was a scientific experiment, Figure 1.23). Users were then shown the ‘participant information’ screen that provided details about the study and gave contact information for the research team (Figure 1.22.2, a). At this point, users could either stop using the app or press the ‘I consent to participate in the study’ checkbox, which subsequently made the ‘Continue’ button ‘tap-able’ (Figure 1.22.2, a).

On tapping ‘Continue’ the Alcohol Use Disorders Identification Test (AUDIT) was presented to all users. The display of questions on the AUDIT was designed to reduce the high rates of attrition associated with asking an excess of questions at an app’s registration point [55]. If an answer indicated subsequent questions were unnecessary those questions were hidden, allowing users to skip straight to the next relevant question. The questions themselves were displayed in a way that meant they could be answered with a single tap, many app forms require two taps for an answer (one to open a list of possible responses, the second to select a response). Text at the top of the screen explained why the questions were being asked and a progress bar at the bottom showed users their position in the registration process. Brief feedback about AUDIT scores followed WHO guidance [56], full details of which can be found in section 1.24.

Baseline demographic questions (age, ethnicity, educational status, country, smoking status, employment status, email address and reason for using the app) were the third and final stage of registration (gender was asked on the AUDIT questionnaire) (Figure 1.22.2, b & c).

## Help section

The help area consisted of information about alcohol, app settings, and information about the app.

### Information about alcohol

‘Information about alcohol’ contained screens explaining the UK’s recommended guidelines for alcohol consumption [1], information about the harms of drinking and the benefits of not drinking as well as information about how to set good goals and how to set action plans. These last two screens were duplicated from their existing position in the Goal Setting and Action Planning modules to make finding them easier for participants who might consider the ‘Help’ area their most natural location. A second link, entitled ‘Can’t stop drinking’ provided links to the NHS Alcohol Addiction Services and Alcoholics Anonymous, for people who may need more extensive support than the app could provide.

### App settings

‘App Settings’ allowed participants to change the time of the reminder that prompted them to complete a log of their drinking. Participants could also turn the reminder off. Text explained that it was best to set this reminder in the morning, when memories are fresh, and at a time that would be optimum for a participant’s morning routine.

### Information about the app

‘Information about the app’ provided links to a number of other screens. ‘Contact’ allowed participants to get in touch with the research team should they have questions or need help; ‘The team’ provided information about the expertise of the research team and ‘References’ contained citations of studies that had informed development of the app. The latter two screens were intended to establish the app as a credible source, a technique associated with the effectiveness of DBCIs [57], and the popularity of apps [13]. ‘The study and you’ contained the same information as on the ‘participant information’ screen in case participants wanted to review that information. ‘Privacy policy’ contained brief information about the study’s data storage procedure and reminded participants they could withdraw from the study at any time. ‘Opt out’ allowed participants to opt-out of the study; this was made a two-step process to prevent unintentional selection. Lastly, ‘Rate this app’ took participants to the Apple App Store where they could leave a review for the app.

All copy in the help area was written concisely and supported by references where relevant. The tone of voice used was friendly and knowledgeable.

### Behaviour change techniques

| BCT | App Location | Section |
| --- | --- | --- |
| 3.1 Social support (unspecified) | Links for additional support/ Tips for drinking less | 1.8.1 |
| 4.1 Instruction on how to perform the behaviour | Tips for drinking less | 1.8.1 |
| 5.1 Information about health consequences | Harms of drinking | 1.8.1 |
| 5.3 Information about social and environmental consequences | Harms of drinking | 1.8.1 |
| 5.6 Information about emotional consequences | Harms of drinking | 1.8.1 |
| 9.1 Credible source | About us | 1.8.3 |
| 10.2 Material reward (behaviour) | Tips for drinking less | 1.8.1 |
| 12.2 Restructuring the social environment | Tips for drinking less | 1.8.1 |
| 12.3 Avoidance/reducing exposure to cues for the behaviour | Tips for drinking less | 1.8.1 |

# The build process

## Choosing a developer and platform

The desired implementation of modules was described in ‘wireframes’, a visual guide that detailed in rough form the content for each screen. Four app development companies were sent these wireframes and a briefing document which summarised the app’s objectives, its key elements, the requirements of a developer, the technical and intellectual property requirements and timescales for completion. Two companies declined to quote, two others returned a proposal and quote. Portable Pixels quoted £22k for two ‘native’ apps, Pocket Apps quoted £28k for an app built in HTML5.

A ‘native’ app runs on a specific computer operating system (i.e. one app would be built for iOS and a separate app for Android), an HTML5 app runs on different operating systems (i.e. the same app would work on both iOS and Android). HTML5 apps run more slowly than native apps [58], and use more device memory [59]; issues which may have an impact on participant engagement [60]. Native apps provide a better user experience [61] and can access the core features of the app more easily [62]. Some features, such as the push notifications required to prompt users to perform certain actions, may not be accessible on HTML5 apps [63].

The advantages of native apps and the preferential quote from Portable Pixels led to their selection for this project. However, given a limited budget and the desire to create iterative versions of the app it was decided to develop on a single platform only, rather than both platforms as had originally been planned. The two main platforms for apps are iOS (iPhone, iPad) and Android. Whilst iOS has fewer users than Android [64] it is considered an easier platform to develop and test on due to the number of different Android devices, browsers and implementations of their operating system [65]. Therefore, iOS was chosen as the development platform.

## Testing and iterating

An ‘agile’ development methodology was adopted [66], this delivered working software at regular intervals and allowed modules of the app to be tested before the app had been built in its entirety. Testing was an extensive and repeated process that involved ensuring not just that all elements worked but that they worked optimally. Paper layouts such as wireframes are an essential guide to development; however, functionality, layout, design and text can only be judged suitable when viewed in the context of the app environment. Consequently, modules went through numerous iterations between their design in the wireframes and the version released to the public.

As mentioned above, attention was focussed on testing the first few screens of the app as these usually result in the greatest amount of attrition [67]. All registration screens went through numerous iterations in order to ensure they were both easy and rewarding to use. The other area most iterated was the self-monitoring module, as this is critical to the feedback module, is a feature users specifically seek out in an alcohol reduction app [14,16] and its data entry elements impose most burden on a user. Therefore, the considerable time and money spent iterating this module toward its most usable form was considered appropriate for experimental and user experience purposes.

Informal testing was undertaken by all members of the research team, their friends and family, and other staff and students at UCL. Testing included the app’s text, design and functionality, the registration and randomisation process, the content different groups were exposed to and the fidelity of data storage. The app build started in September 2014 and a first version was released for testing in May 2015. Formal testing took the form of a usability study of user views toward the app [3].

## How decisions about what to include were made

The app building process requires numerous decisions to be made about the form and function of every element on every screen. These decisions can increase engagement, encourage data entry, create a positive user experience and promote behaviour change. Apps that are easy and rewarding to use are likely to be used more often [3] and are more likely to receive positive reviews and word-of-mouth recommendations, which may lead them to be chosen in preference to the similar apps that exist [68,69].

Decisions regarding implementation of the modules were informed by the published research literature for usability and user experience (e.g. [70–73]), Apple’s iOS Human Interface Guidelines [74], regular consultations with the research team, and recommendations from the software developers. Module implementation was later refined in response to feedback from the usability study [3].

In addition, alcohol reduction apps downloaded for the content analysis study [13] were used and informally reviewed for the approach they had taken to implementing self-monitoring, feedback, action planning and goal setting. The aim was to gather understanding of what the experience was like of using these particular BCTs and the app as a whole. I was the only user. Goals and action plans were set, drinks were recorded and feedback was noted. The questions asked included: was the process of entering data straightforward and intuitive; did it result in a positive or negative change in affect; was entering data something that seemed beneficial to repeat; was the process as a whole something that should be learnt from or avoided? For example, when recording consumption, some apps allowed users to choose their drink from a list. However, there are over 1,500 different bottled ales in the UK [75], with more added on a regular basis and hundreds of other types of beer, wine or spirits available; any list of alcoholic drinks was unwieldy to use, is bound to be quickly out-of-date, and was, as such, impractical. In addition to using the apps, user views toward the implementation of BCTs were informally studied by examining reviews left on the app stores. For example, users expressed frustration with an inability to set exact levels of ABV (alcohol by volume) in the NHS DrinkTracker app; a drink could be 4%, 4.5% or 5%, but not 4.6%, 4.7% or 4.8%. A need for this level of precision was a determining factor for some users, who commented that they would choose an app on that basis alone. This work informed the brief given to the app developers which outlined how we wanted each of the modules to be implemented. Implementation was later refined by internal testing (1.10) and the usability study [3].

# Changes made in response to the usability study

## Determining which changes to make

Changes arising from the usability study were implemented only if they were in accordance with the aims of the study and also if they either improved, or at the very least did not negatively impact on, the user experience (UX) as indicated by the usability data. For example, a change that might make evaluating the effectiveness of one of the modules more difficult was not considered, nor was a change that might result in a poorer UX. Creating a good UX was considered to have comparable importance to answering the research question because it is not possible to reliably evaluate the effectiveness of the modules without participants’ repeated use of the app.

Changes meeting both the above criteria were more numerous than could be implemented with the budget and time available. Decisions about which changes to implement were determined in the following order: changes important to participants in both the think aloud study with first-time users and the semi-structured telephone interview study with experienced users were implemented; changes that were requested or suggested by participants in only one study were implemented if they appeared to be commonly held; issues not commonly held were implemented if they appeared highly important to some participants, or if they advanced the aims of the study and/or if they improved the UX. When contradictory requests for change were encountered, the change that most aligned with our research question, or which provided the most positive impact on UX (without detracting from our ability to answer the research question), was implemented.

## Normative Feedback module

Users wanted to have the ability to interact with the normative feedback and be able to change what group they compared themselves with, in particular a comparison with just drinkers. By including the “only drinkers” comparison, users were considered less likely to immediately discount the information. Drinkers were defined as “anyone who has had a drink in the last year, even if it was just one!”.

Users also expressed a desire to be able to re-take the AUDIT and update their normative feedback. However, there was an issue of bias with follow-up in the trial as it may encourage users to do the follow-up questionnaire and those receiving the minimal version would not have access to this feature. There is the potential to include this feature in the optimised app if the normative feedback module appears effective.

A lot of users found the normative feedback shocking and were on the defensive. A number also believed that others were lying about their drinking as a way of making sense of the data. There was text in a pop-up link labelled ‘more information on this’ which users found reassuring and gave the data credibility. However, a lot of users did not spot this or chose not to read this. Therefore, this pop-up link was removed and two new screens of text were added. The first came before the feedback with the aim of managing the user’s outcome expectancies and the second after the feedback to increase the user’s self-efficacy so they felt in a position to do something about their drinking.

## Cognitive Bias Re-training module

Users wanted to know why the game should work though appeared to like the idea of re-training your mind. A number doubted it worked and/or were unsure of why they were playing the game. Therefore, the purpose of the game was made clearer on the main menu screen and a separate sub-section was included explaining how and why it should work.

Not all users found the instructions completely clear and some requested it to be made clearer with just a quick graphic on how to do it. Some users appeared to ignore the text on the instructions. An additional graphic was included in the instructions to clarify how the game worked with a link to play the game straight from completing the instructions. There was also confusion with the tall/wide description of images so the instructions were changed to portrait/landscape.

## Self-monitoring and Feedback module

#### Recording drinks

Users valued the ability to record an alcohol free day, so this option was included on the main ‘add drinks’ screen (Figure 1.21.4.2, a) in addition to its previous location in the calendar (Figure 1.21.4.10, b). A calendar appears when the date is tapped as this was considered preferable to the back and forward arrows previously used (Figure 1.21.4.1). An information button was added to the record drinks screen to provide more information about each element on the screen (Figure 1.21.4.2, c). The term ‘Favourites’ was changed to ‘Regulars’, as some users thought favourites an inappropriate word for an alcohol reduction app (Figure 1.21.4.1). The alert prompting users to complete their diary was edited to remove any reference to alcohol in order to allay fears of users who were worried about other people knowing they were attempting to reduce their consumption (Figure 1.21.4.6, a). The icon for wine was coloured red instead of white as some users had difficulty locating the wine option on the main add drinks panel (Figure 1.21.4.1).

#### The Mood Diary

Participants commented that the previous approach of comparing no drinking days with drinking days produced inaccurate mood, productivity, clarity and sleep comparisons as they often felt in a positive mood the morning after light drinking (because they had been socialising with friends, for example). Therefore, the Mood Diary was changed to compare no drinking/light drinking days with heavy drinking days (heavy drinking was defined as the consumption of more than six units in a day). The error messages that prompted users to complete required fields was amended to provide information about what questions remained to be answered (e.g. “Please answer if you have any more drinks to record”) (Figure 1.21.4.7, c). An information button was added to provide more information about the purpose of the Mood Diary, how it should be completed and where responses were displayed (Figure 1.21.4.8, a).

#### Feedback

Participants in the usability study frequently expressed disappointment that feedback about progress toward their goals was difficult to find. Signposting of feedback was improved by the creation of a new ‘Your achievements’ section, which listed the title for each goal and provided text feedback related to whether the goal was exceeded, hit, nearly hit or missed in the previous week (feedback and icons as described in section 1.4.3, above). Tapping the title for each goal took users to the Goal Feedback screens described in section 1.4.5, above. The ‘Your achievements’ area also displayed the longest number of continuous days drinking had been recorded (e.g. “Keeping your diary two days in a row”, Figure 1.21.4.3) in an attempt to encourage regular self-monitoring.

Other changes included making each bar of the Units graph on the dashboard display a summary of drinks for that week when tapped (Figure 1.21.4.5, b), and displaying an icon if no units at all were consumed that week (Figure 1.21.4.3). Lastly, when all tasks within the ‘We suggest’ area had been completed, users were rewarded with a congratulatory message “Good work, you’re all done today” (Figure 1.21.4.3).

The dashboard previously contained three graphs for each of ‘Units’, ‘Calories’ and ‘Money’. Users noted that the shape of the units, calories and money graph would always be the same, and suggested that alcohol-related calories and money could be better expressed in the form of numbers. Therefore, these graphs were changed to show Calories from (or money spent on) alcohol this week, the difference between this week and last week and the total calories from (or money spent on) alcohol since the app was downloaded (Figure 1.21.4.5, a).

Changes to the calendar consisted of the addition of an orange bar under a date to indicate a light drinking day (previously the calendar showed only red for heavy drinking day, green for a no drinking day and grey for no record). The total number of alcohol free days recorded so far is now shown at the bottom of the calendar (Figure 1.21.4.10, a) and each daily record displays the total number of units consumed (Figure 1.21.4.11, a).

## Action Planning module

Confusion expressed by participants about how to set action plans was addressed in two ways. First, more examples of alcohol-related action plans were provided, adapted from action plans developed by previous users of the app and made accessible via the information button on the ‘Create an action plan’ screen (Figure 1.21.5.2, b). Secondly, if users tried to save an action plan without both the ‘If’ and ‘Then’ fields being complete, a detailed error message directed users to the area that needed completing. For example if the ‘Then’ area was empty the error message said “Please enter your ‘Then’ text” (the previous version gave users a generic notification to ‘Please complete all fields’ Figure 1.21.5.2, a). To resolve confusion about when an action plan had been successfully set the app displayed a confirmation message and played a sound after a user had clicked the Save button on the ‘Create an action plan’ screen (Figure 1.21.5.2, c).

## Identity Change module

Users were confused by the name “Identity” and expected a profile-type section so the name on the module within the app was changed to “Drink + Me” in the menu bar at bottom and at top of screen. A clearer explanation of the purpose of this module was included on the main menu screen for the module. In addition to this, text was added at the beginning of each section to clarify its purpose. Throughout the module, the use of “I” or “you” was removed to make it more general as some users felt it was targeted at them in a negative manner. A default image of a smiley face in the “I am…” sub-section was added so that a user could still use the section without using their own photo.

## Goal setting module

The major change to the goal setting module was to remove the option for monthly goals (which were considered distal goals and therefore less effective) and change the goal period from a rolling week to a fixed Monday to Sunday (which users said they preferred). Otherwise, only minor changes were made to this module. A confirmation message and sound played after a goal had been successfully set (Figure 1.21.1, f), small graphical changes were made to improve the legibility of text and some of the sections were renamed in order to clarify the content they contained (e.g. ‘Good goal setting’ was renamed ‘How to set good goals’).

## Other elements of the app

Eleven of the 12 participants in the first usability study said that the dashboard, the first screen seen after completing registration, was confusing and visually uninspiring and wanted to know what they should be doing when first using the app. To provide this information, the revised version of the app incorporated a guide with three steps: 1) Set a goal; 2) Log some drinks; 3) Explore (Figure 1.22.2, e). These were considered the elements most important to the initial use of the app and were limited to three to prevent overwhelming users with tasks. Once complete, each step was greyed-out and had a green tick added to it in order to provide a sense of accomplishment (Figure 1.22.2, e).

An Info button was added to each screen of the app in order to help users understand what the purpose of the screen was, what each element on the screen did and how they should be completed or followed. To help users understand the existence of and purpose of this button, a message appeared on screen alerting users to its presence (Figure 1.22.2, f). To avoid over-promoting this feature to the potential annoyance of users, the alert was shown on the 1^st^, 3^rd^, 10^th^ and 20^th^ screen visited, after which it was assumed users were aware of the info button’s existence and the alert was not shown again.

User burden was reduced by limiting the number of daily tasks that users were asked to complete and renaming the area containing these tasks to a more gentle ‘We suggest’ from its previous, more directive, title ‘Things to do today’ (Figure 1.21.4.3). The only daily task users received was a prompt to record their drinking. Every three days an additional prompt suggested that users visit a different module of the app. Prompts were displayed randomly rather than sequentially in order to ensure that all modules were promoted equally. Only users in the experimental condition were prompted to use a module; participants in the control condition were not presented with the prompt.

Navigation on the app was improved by ensuring that the tab bar (which sits at the bottom of the screen and provides links to the Dashboard, Progress, Add Drinks, Game and Identity) was retained on all screens; previously it had not been visible on some screens. Bugs where the navigation was broken or did not work as expected were fixed.

The app was improved graphically by adding icons to the dashboard and progress screen (Figures 1.21.4.4 & 1.21.1). The tab bar icons were made a little bigger and slightly darker to improve legibility. The text used within the app was made friendlier and more informal. The prompt asking users to rate the app was changed so that it was triggered after the app had been opened seven times rather than three (in order to gain reviews from people who had more experience of using the app).

Any bugs identified by participants in either study or were identified by the research team in their use of the app, which prevented the full functioning of the app, were resolved.

## Follow-up

The follow-up procedure was improved in order to reduce the number of users who abandoned the app without completing registration and to increase the number of users who qualified for inclusion in the study.

The original version of the app displayed a welcome screen (required by Apple, containing a logo and the tag line “Get healthy, save money and lose weight, just by drinking a bit less alcohol”), a screen listing key features of the app and the ‘participant information’ screen. To promote the experimental nature of the app from the start, and encourage more participants to complete follow-up measures, the tag line was changed to “Get healthier and help science by using this app”. To make it easier for users to get to the app’s content more quickly, the screen listing key features was removed, leaving just the welcome screen and the ‘participant information’ screen. The prize draw amount, used as an incentive for users to leave their email address, was increased from £100 to £500. If a user left the email field blank, a prompt reminded them about the scientific nature of the study and the prize draw amount and asked if they wanted to reconsider leaving an email address (Figure 1.21.2.2, c). If they continued to leave the email field blank the prompt was not displayed a second time. In addition to being emailed a link to complete the follow-up questionnaire, the same questionnaire was also presented in the app a month after users had downloaded the app on the ‘We suggest’ area of the dashboard (Figure 1.21.4.3).

Registration for the app was simplified in the following ways. A sentence was added to the top of the AUDIT and ‘About You’ questions to explain why these questions were being asked. The year of birth drop-down field started at 1999 in order to reduce scrolling (users born after that date would be ineligible for the study). A question was added to determine if users were serious about reducing their consumption or just browsing the app (users just browsing were ineligible for the study). The alert asking users to accept notifications from the app was moved to the point where registration was complete, rather than when on first downloading the app as it was thought better to ask permission to notify users after they had gained some benefit from using the app (Figure 1.22.2, d).

## Screenshots: Intervention modules

### Goal setting

| 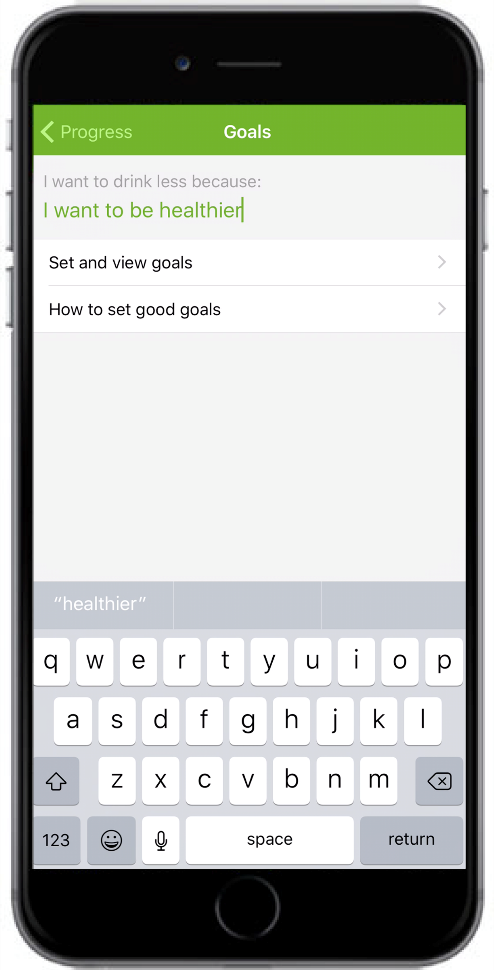a | 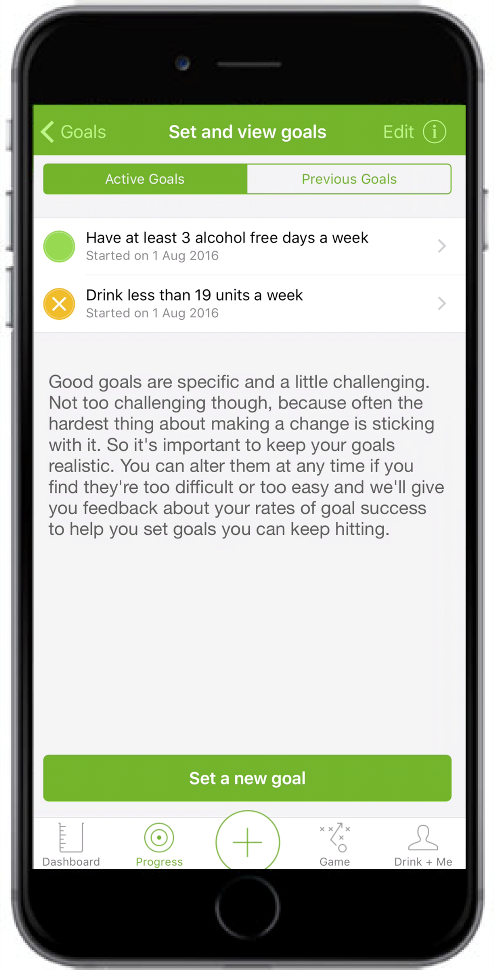b | 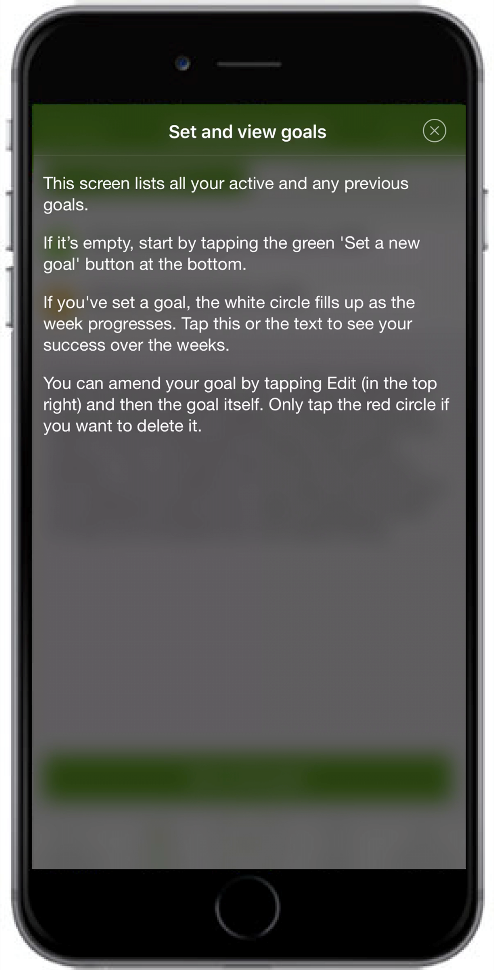c |
| --- | --- | --- |
| Users could set goals, get info on how to set good goals, and set an overarching goal for drinking less (displayed on the dashboard, Figure 1.21.4.4), | ‘Set and view goals’ allowed users to set new goals and see summary feedback about current goals | The Set and view goals info button provided more information about how new goals could be created or existing ones edited |

| 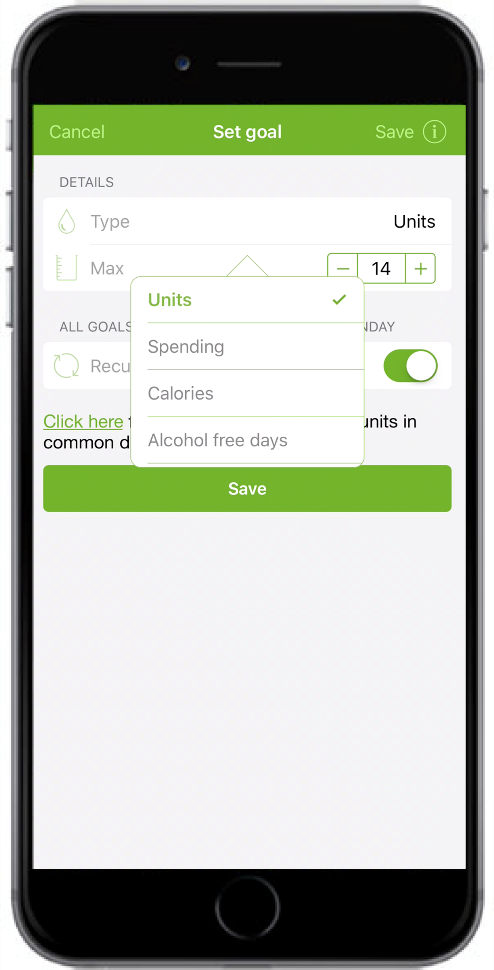d | 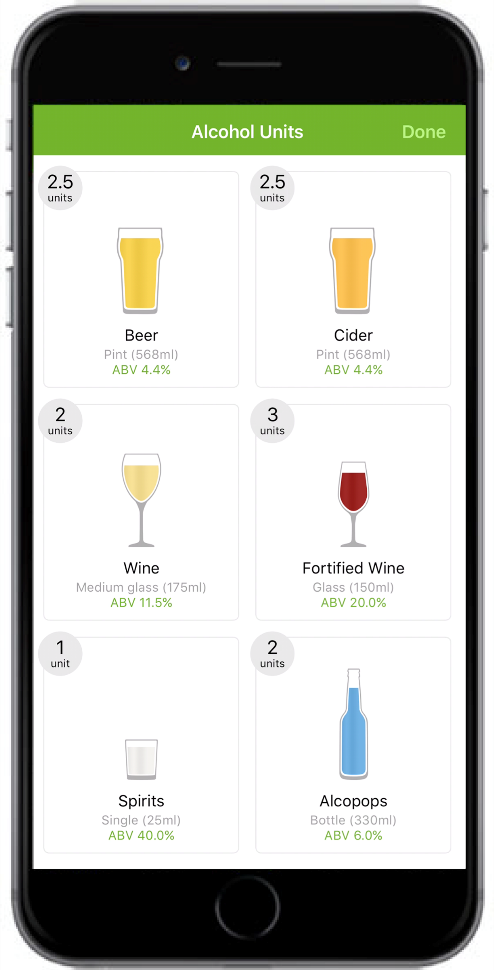e | 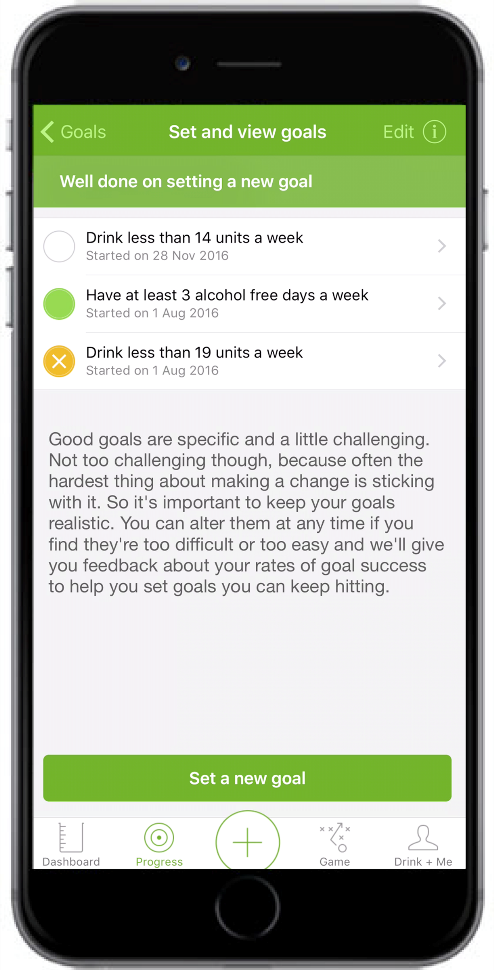f |
| --- | --- | --- |
| Setting a goal involved two steps: 1) choose a type of goal; 2) Set the target. A goal recurred by default, this could be turned off | If a Units goal was selected a link provided users with a guide about the number of units in typical drinks | A congratulatory message appeared (top of screen) and pleasing sound was played when a goal had been successfully set |

### Normative Feedback

#### Assessment of normative misperceptions and providing normative feedback

| 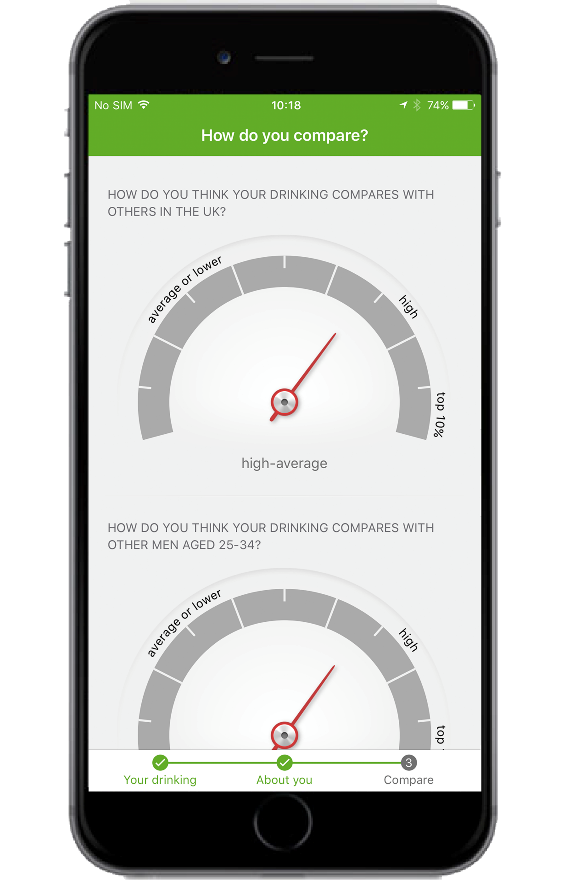a | 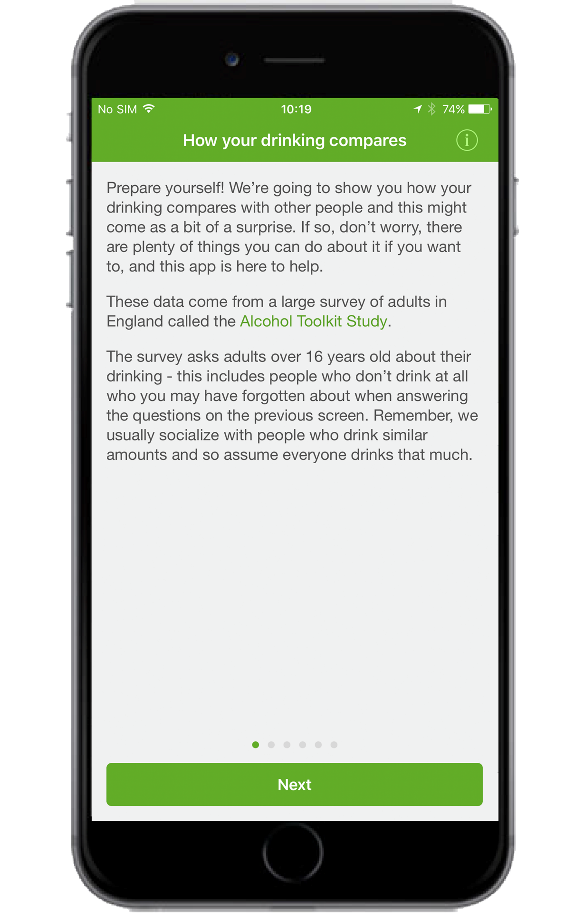b | c 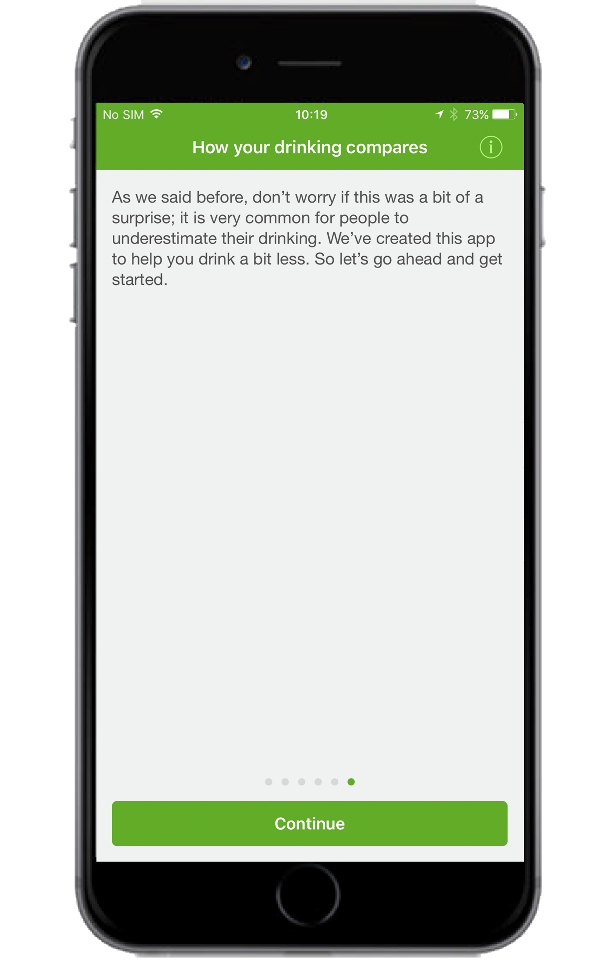 |
| --- | --- | --- |
| The normative misperceptions assessment showed two gauges for users to ‘tap’ or ‘drag’ the red needle to respond to each question. | A screen of text before the normative feedback was presented to manage the user’s outcome expentancies. | A screen of text after the normative feedback was presented to increase the user’s self-efficacy after receiving the normative feedback. |

#### Providing normative feedback (UK sample)

| 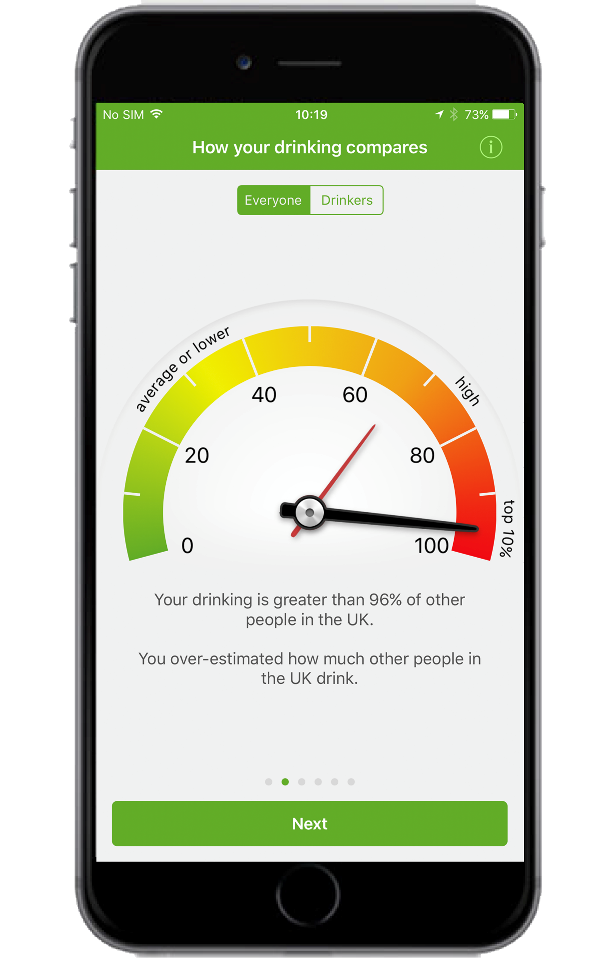a | 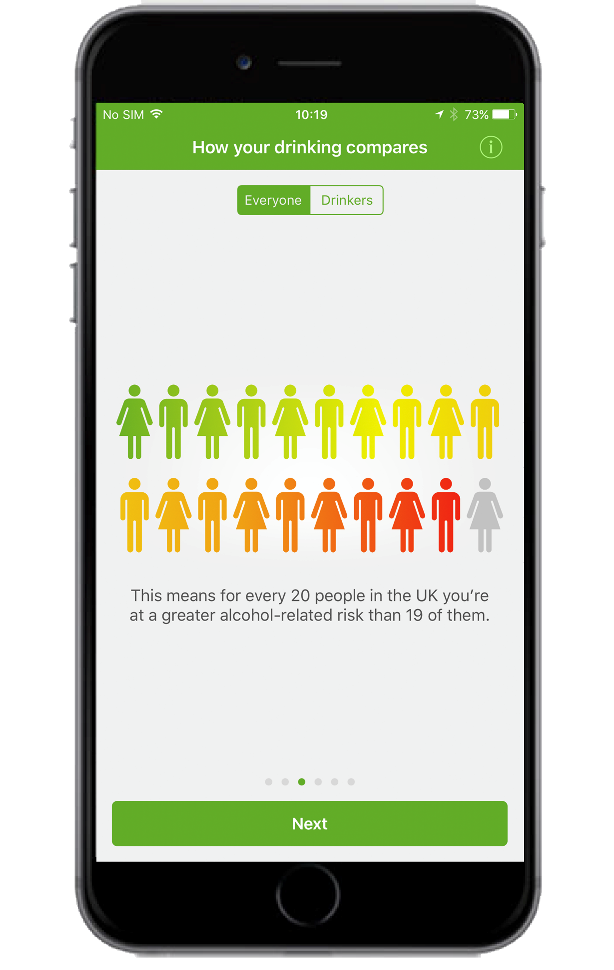b |  |
| --- | --- | --- |
| Normative feedback was provided on how the user’s drinking compared with the rest of the UK population (black needle) and their belief (red needle) | Normative feedback delivered through an infographic to illustrate how their level of risk compared with the rest of the UK population |  |

#### Providing normative feedback (age/gender sample)

| 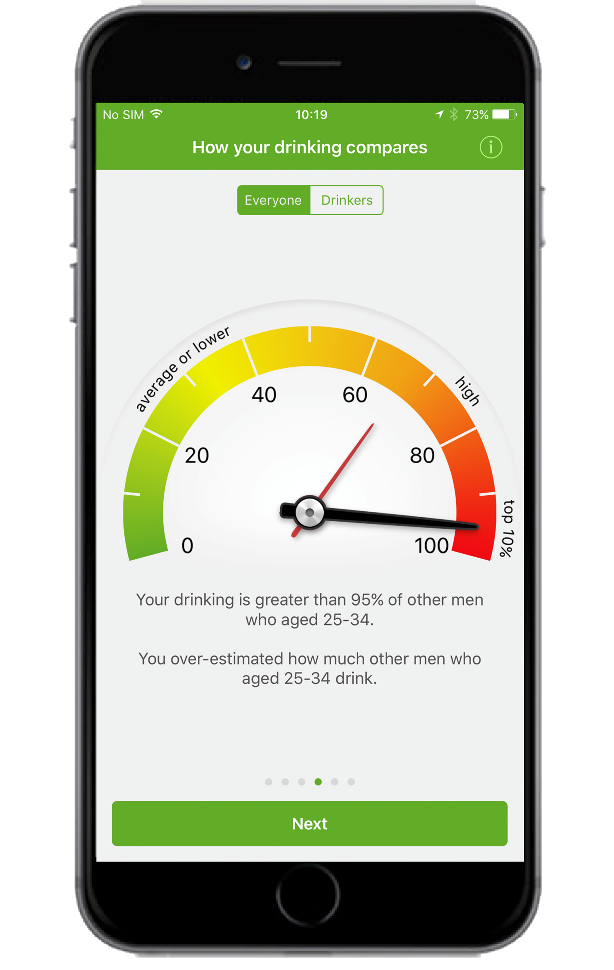a | 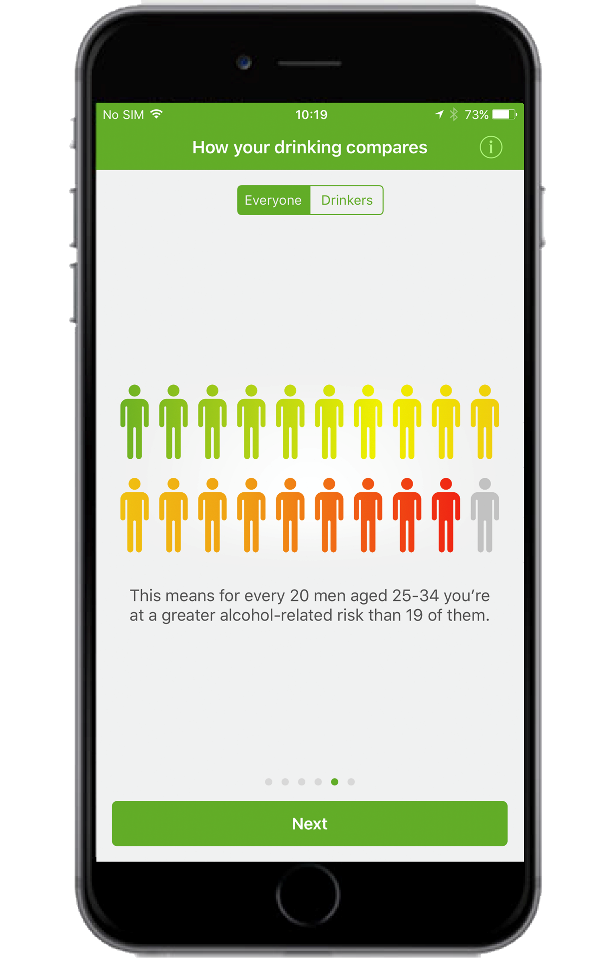b |
| --- | --- |
| Normative feedback was provided on how the user’s drinking compared with the rest of their age/gender sample (black needle) and their belief (red needle) | Normative feedback delivered through an infographic to illustrate how their level of risk compared with the rest of their age/gender sample |

#### Progress screen and ‘Review your drinking’

| 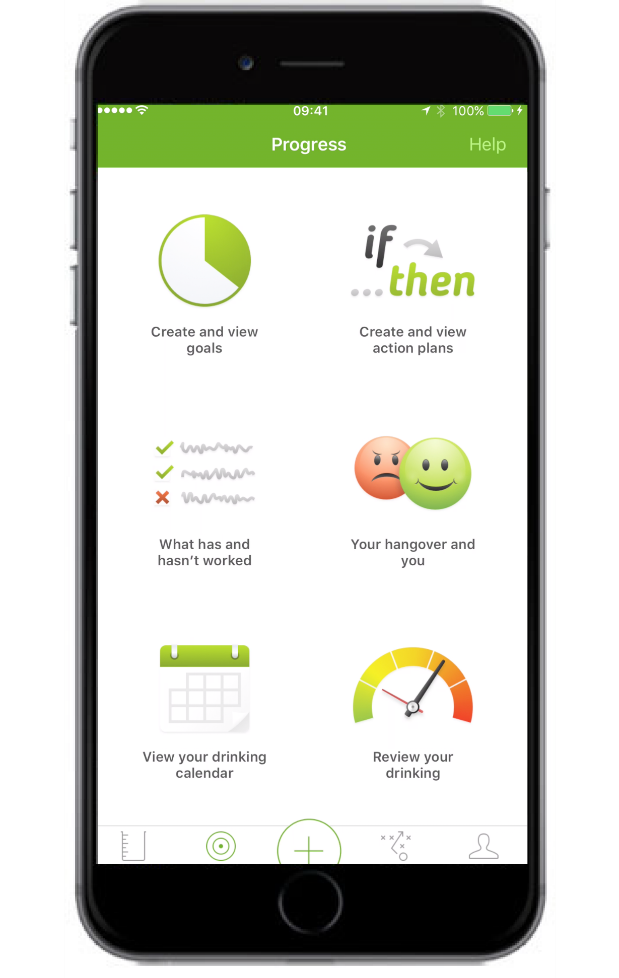a | 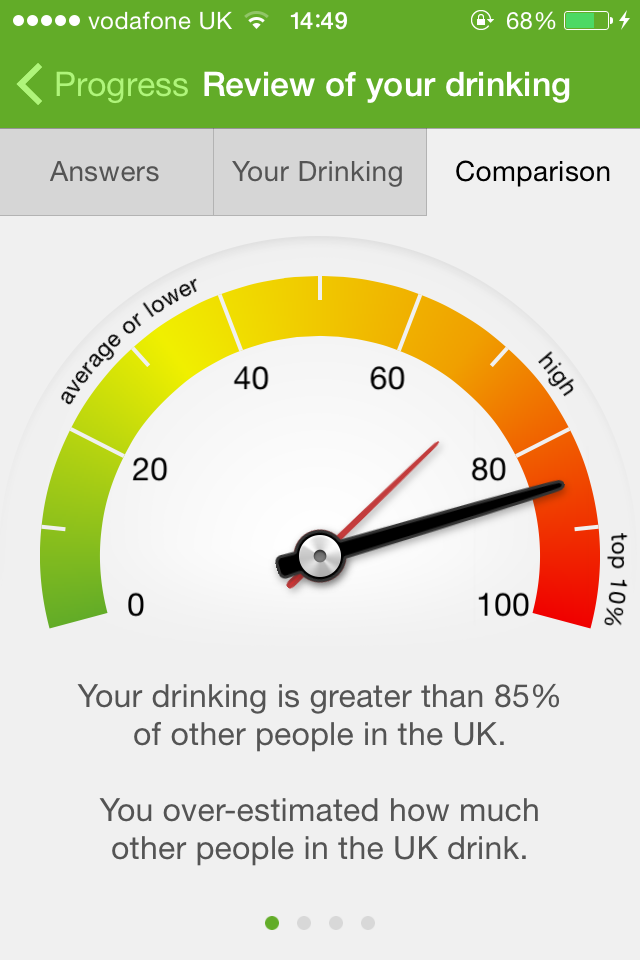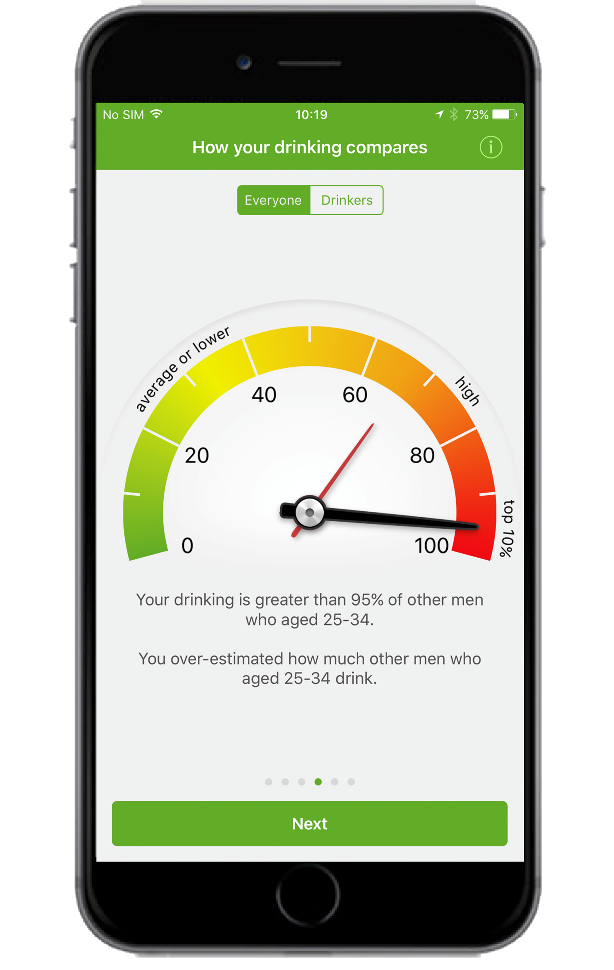b | 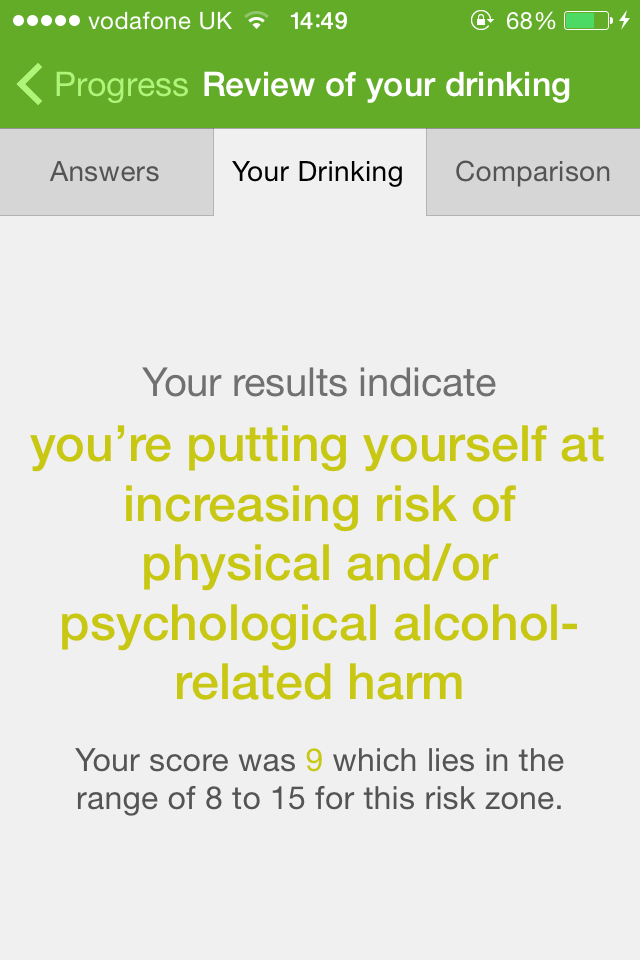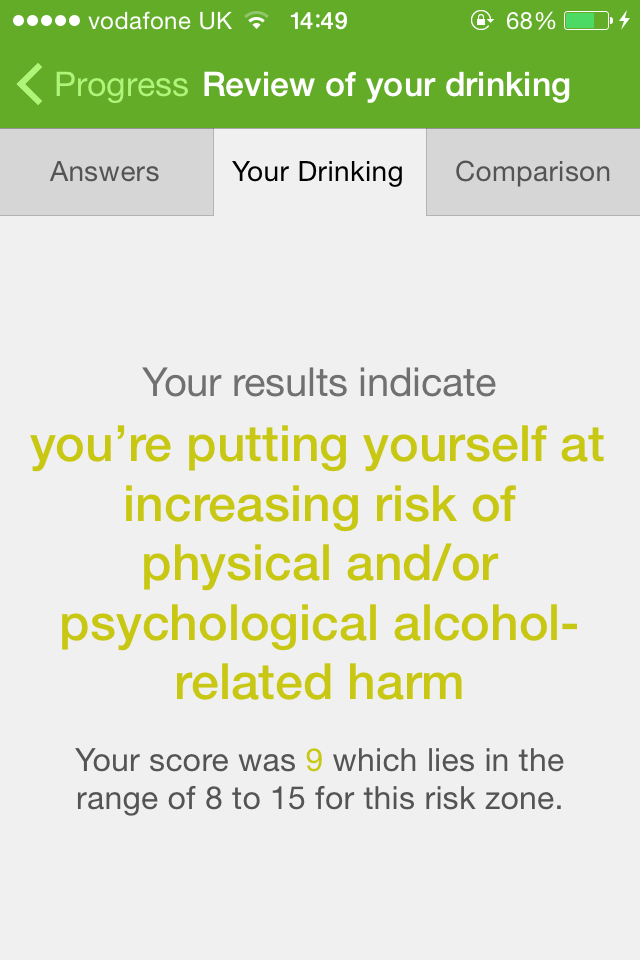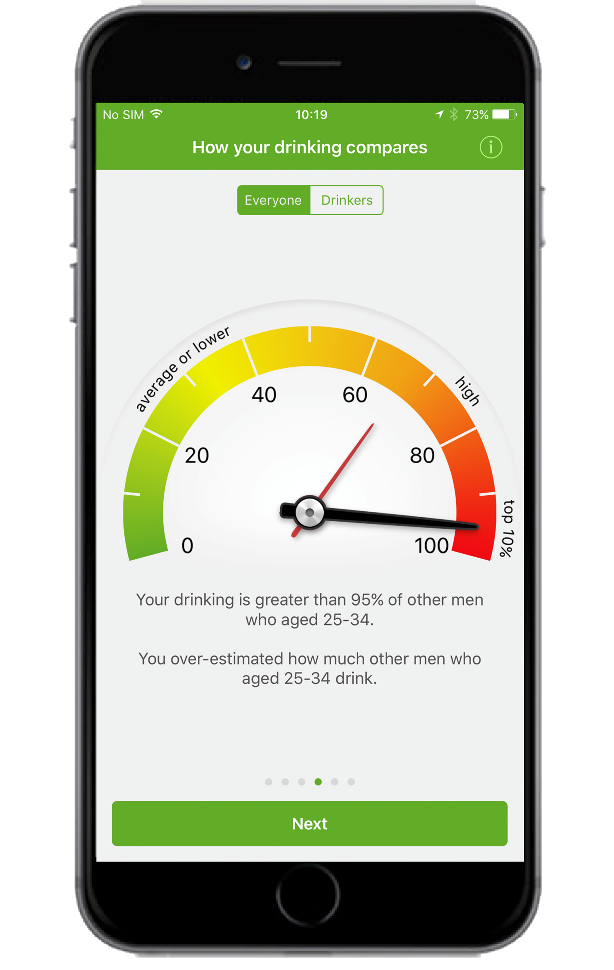c |
| --- | --- | --- |
| ‘Review your drinking’ could be accessed via the Progress screen (bottom right). | The normative feedback could be accessed through the ‘Comparison’ tab. | The users’ AUDIT result could be accessed through the ‘Your Drinking’ tab and their AUDIT responses through the ‘Answers’ tab. |
| Minimal version a 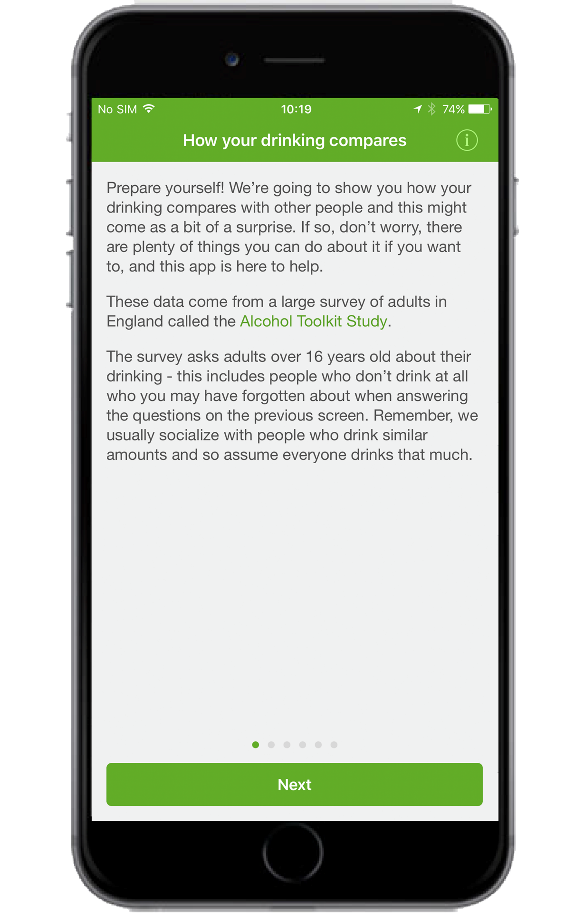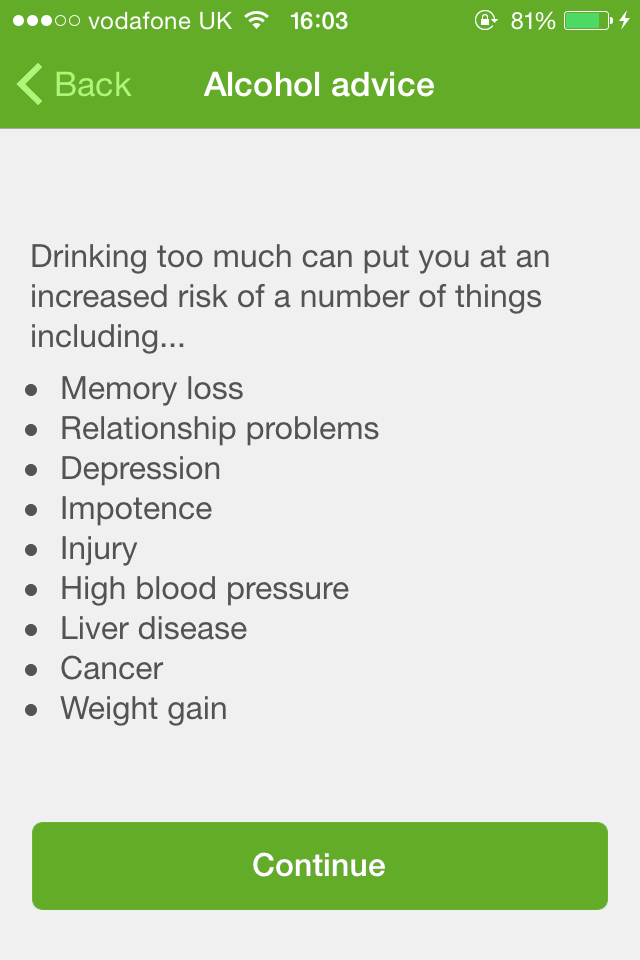 |  |  |
| Control participants were provided with text from the ‘Brief Advice Tool’ from Public Health England |  |  |

### Cognitive Bias Re-training

#### Main screen, ‘How re-training your mind works’ and ‘Previous scores’

| a 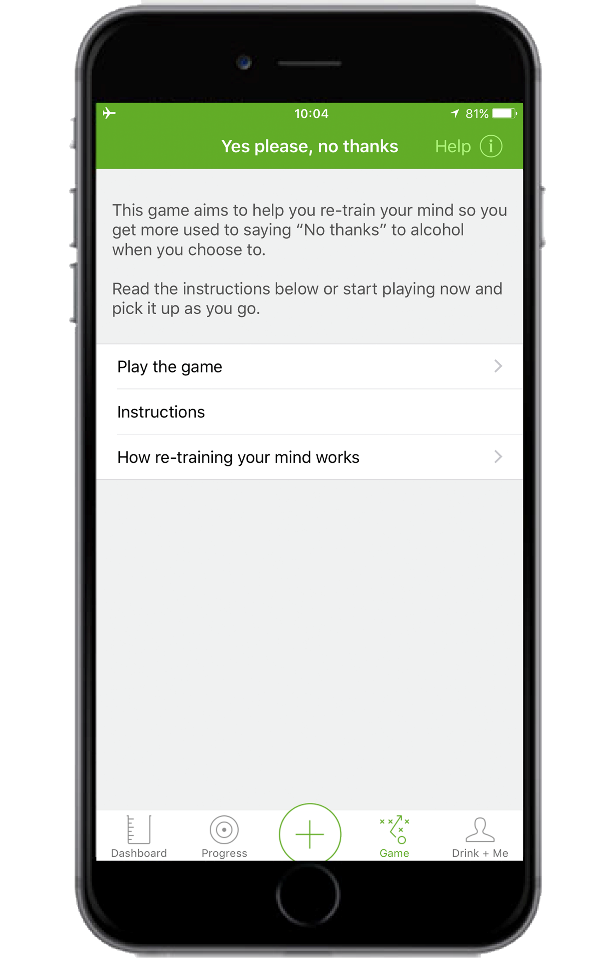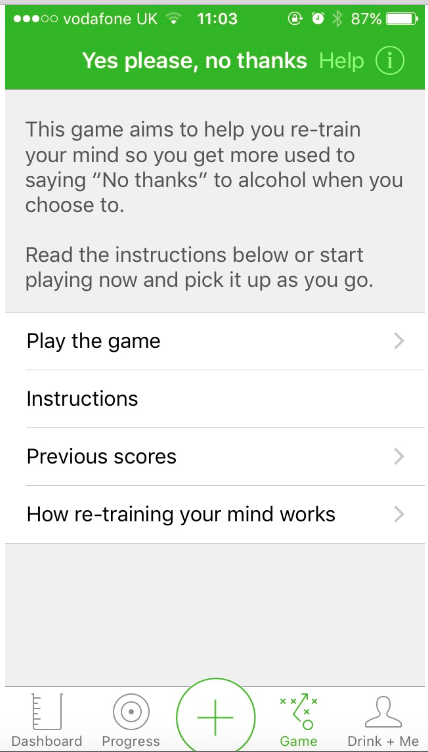 | b 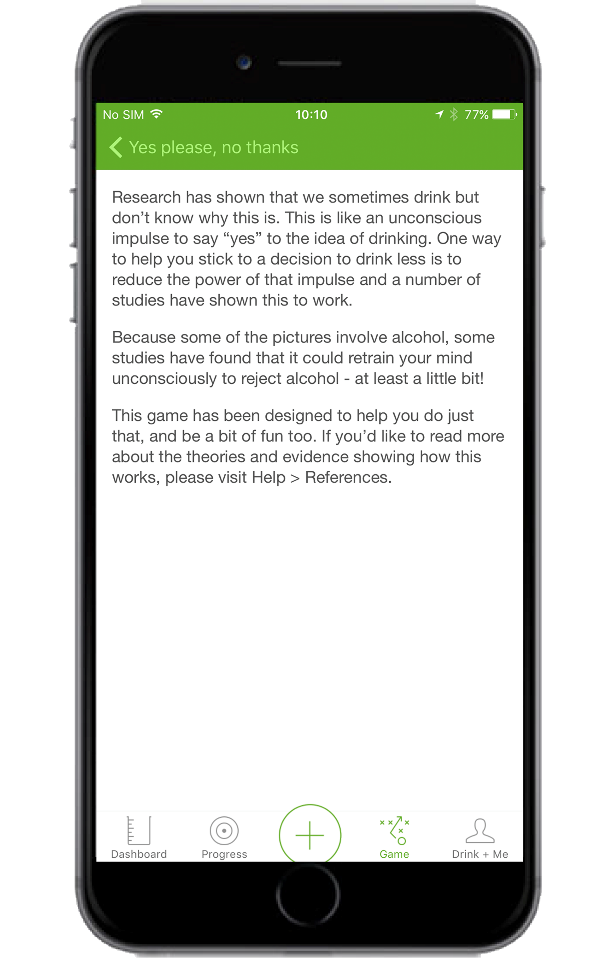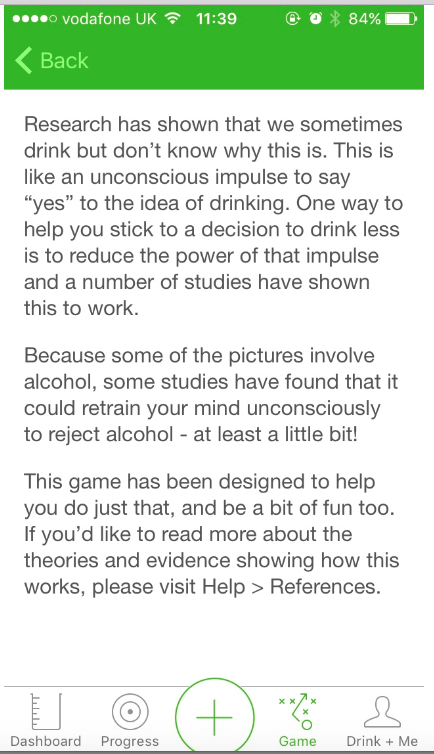 | 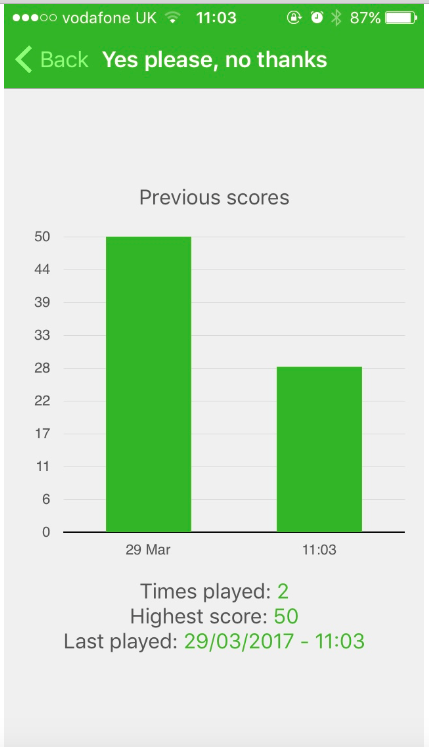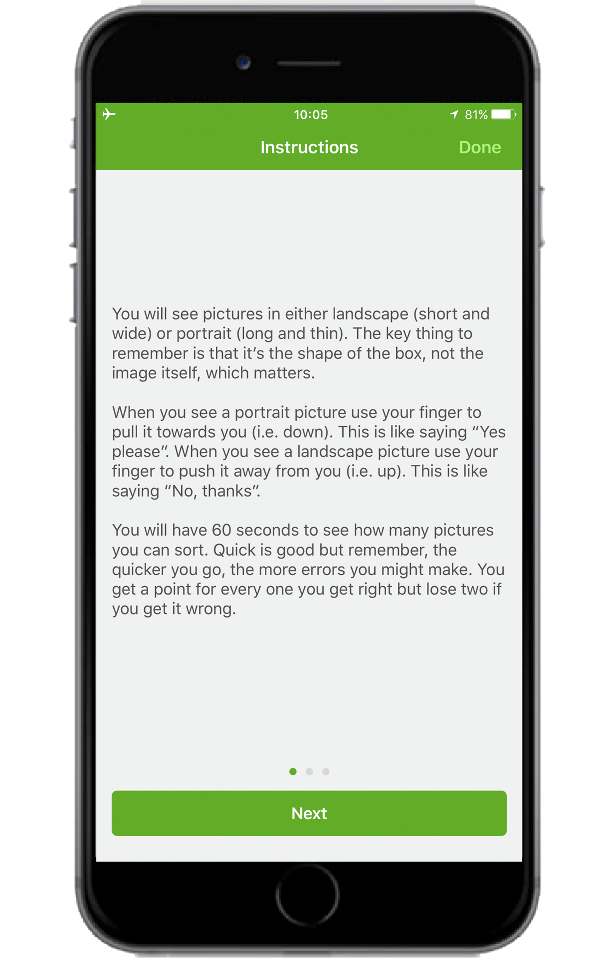c |
| --- | --- | --- |
| The main screen includes links to other sections of the module and a sentence to emphasise the aim of the game. | A screen of text explaining how the game might help the user reduce their consumption of alcohol. | The Previous scores screen showed a bar chart of all previous game scores with the user’s highest score shown below the chart. |

#### Instructions on how to play

| 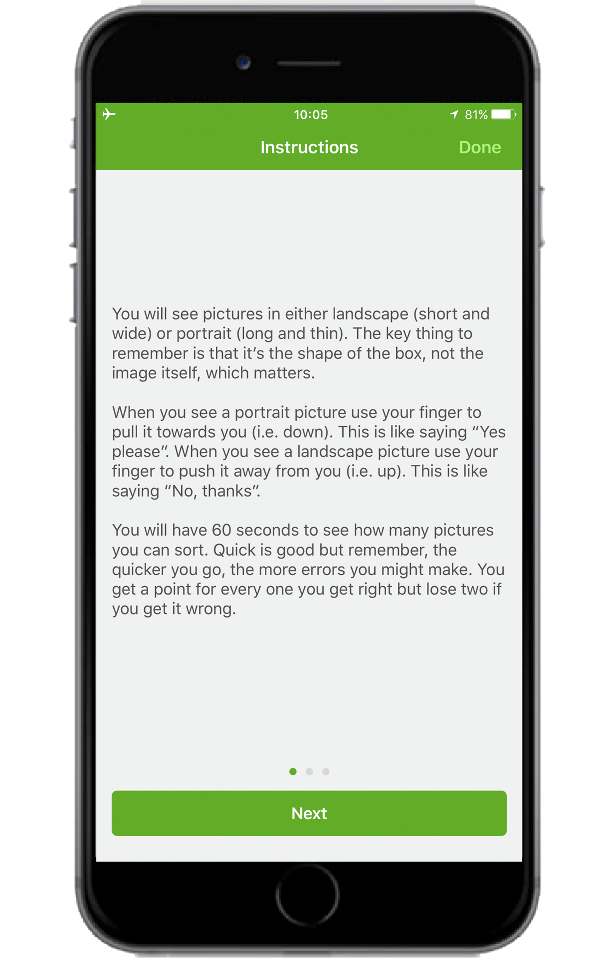a | 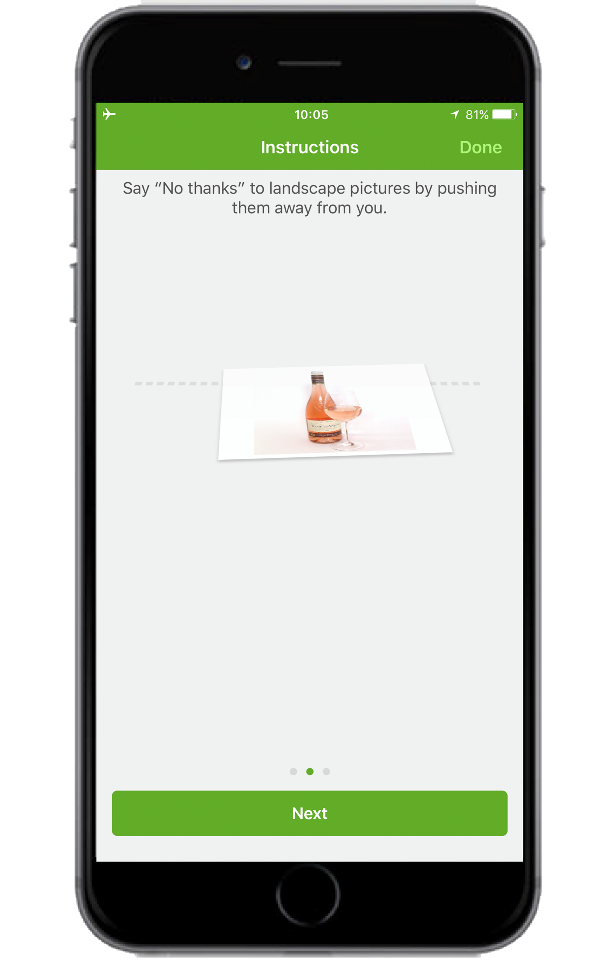b | 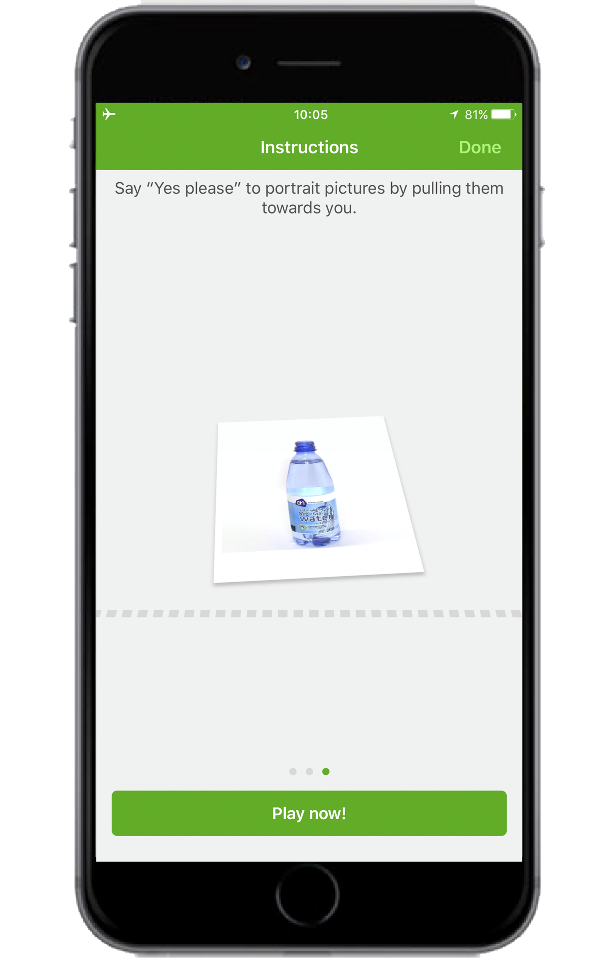c |
| --- | --- | --- |
| The first screen of the instructinos with text about how to play the “Yes please, No thanks” game. | The second screen of the instructions with a graphic showing the “No thanks” response of pushing the image away (up the screen). | The third screen of the instructions with a graphic showing the “Yes please” response of pulling the image closer (down the screen). |

#### The Game and Results

| 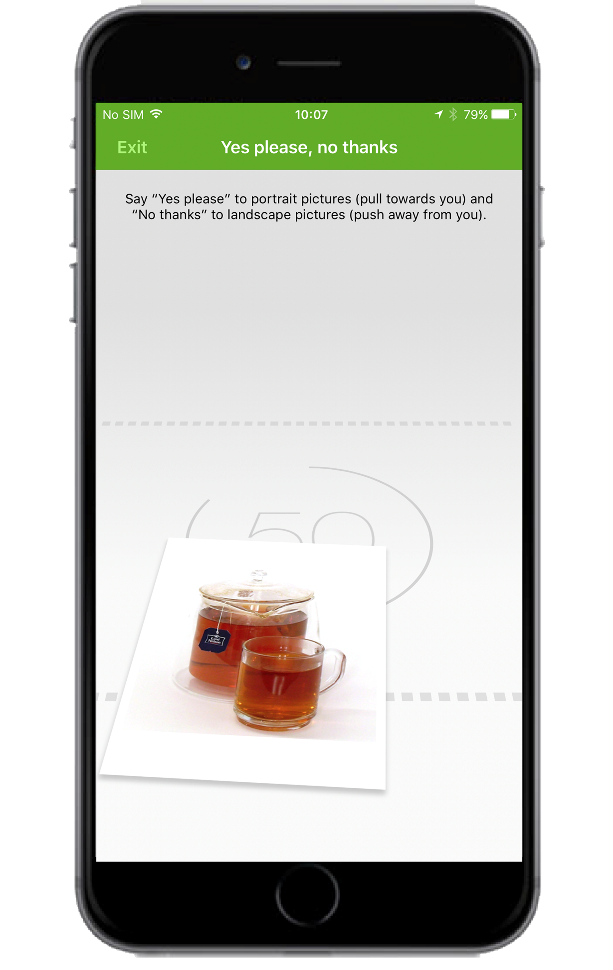a | b 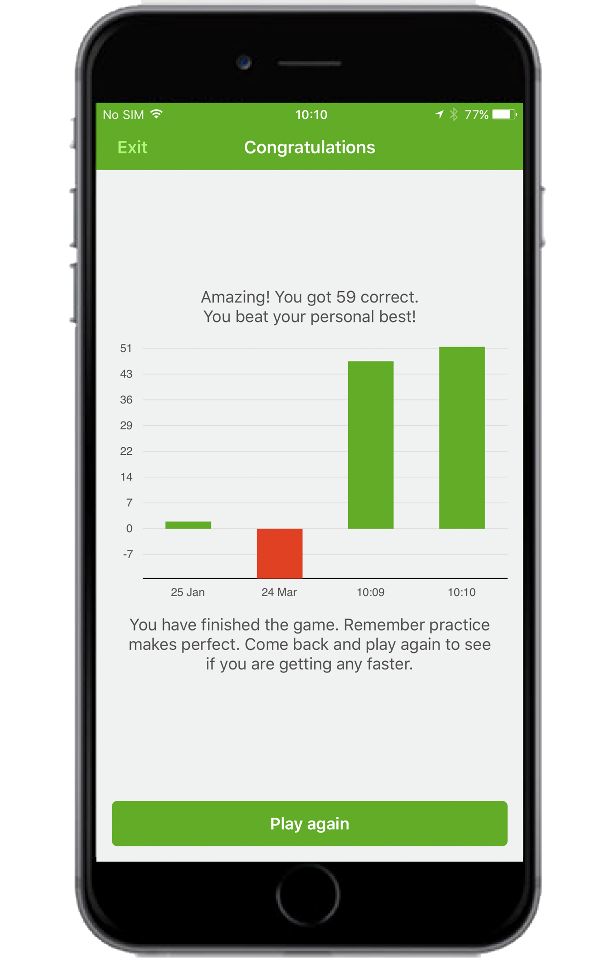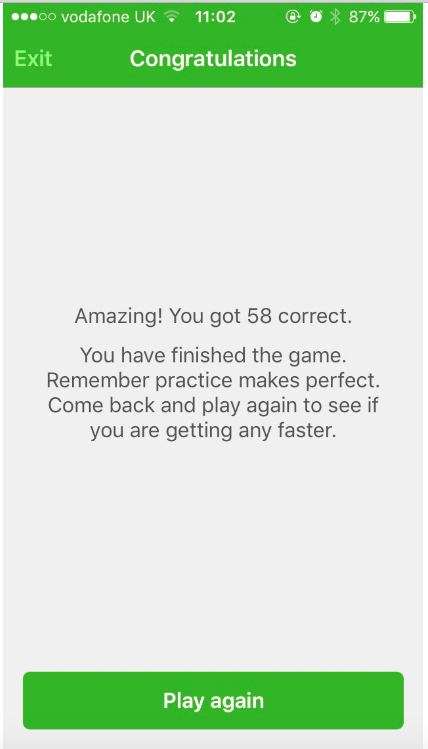 | c 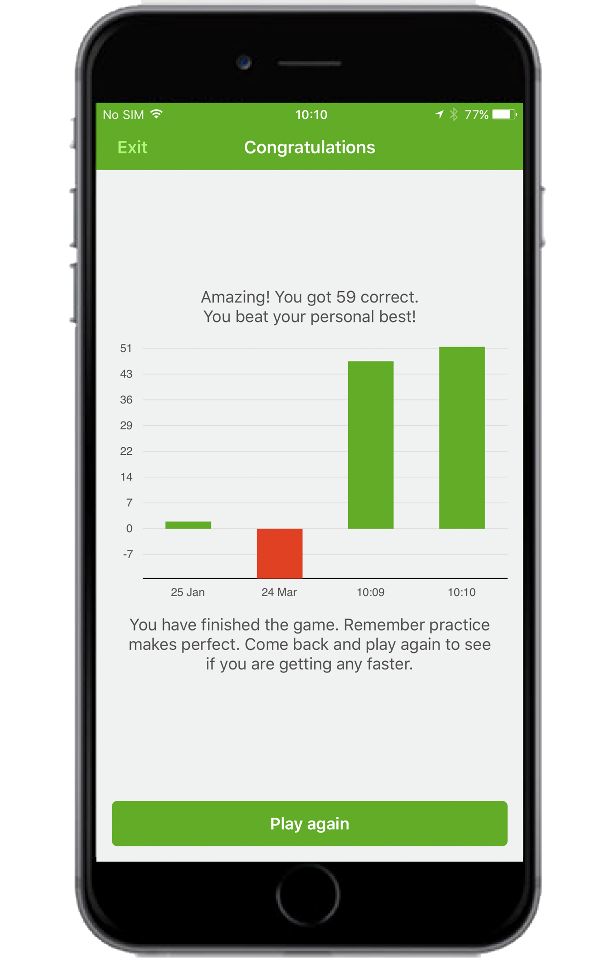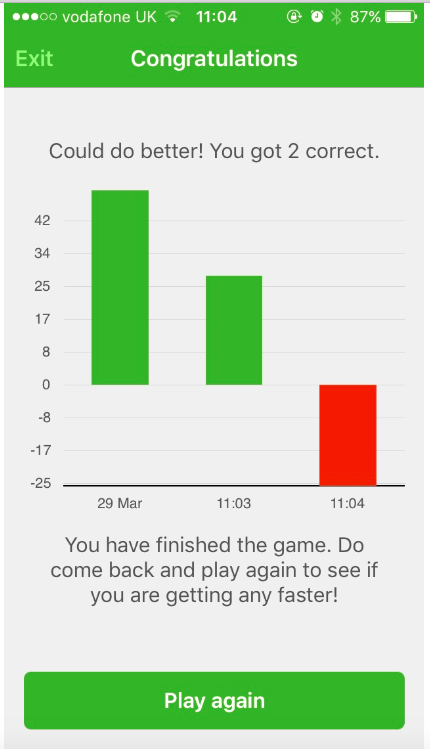 |
| --- | --- | --- |
| The screen shown when playing the game with images ‘zooming’ depending on the direction the user moved the image. | The screen shown after the game was completed for the first time, with the score for that first game. | The screen shown after the game was complete with the score for that game, and graph showing all previous scores. |

#### Minimal version

| 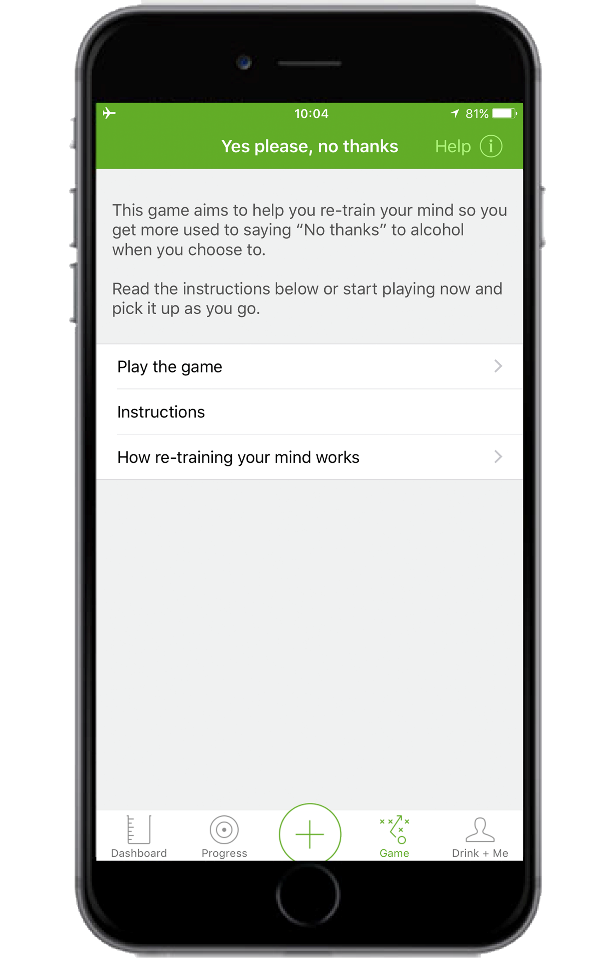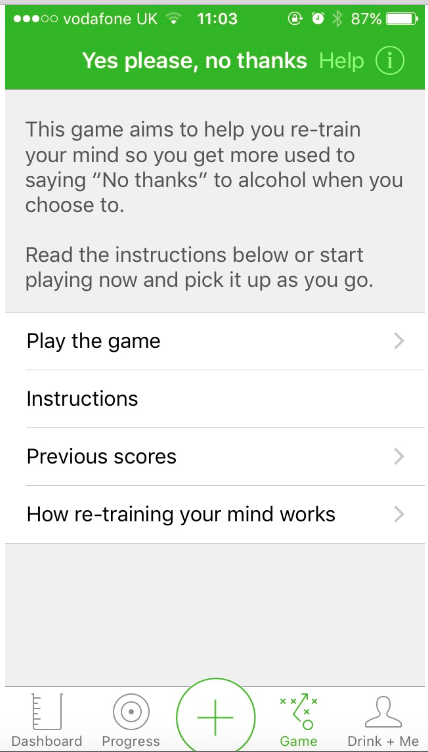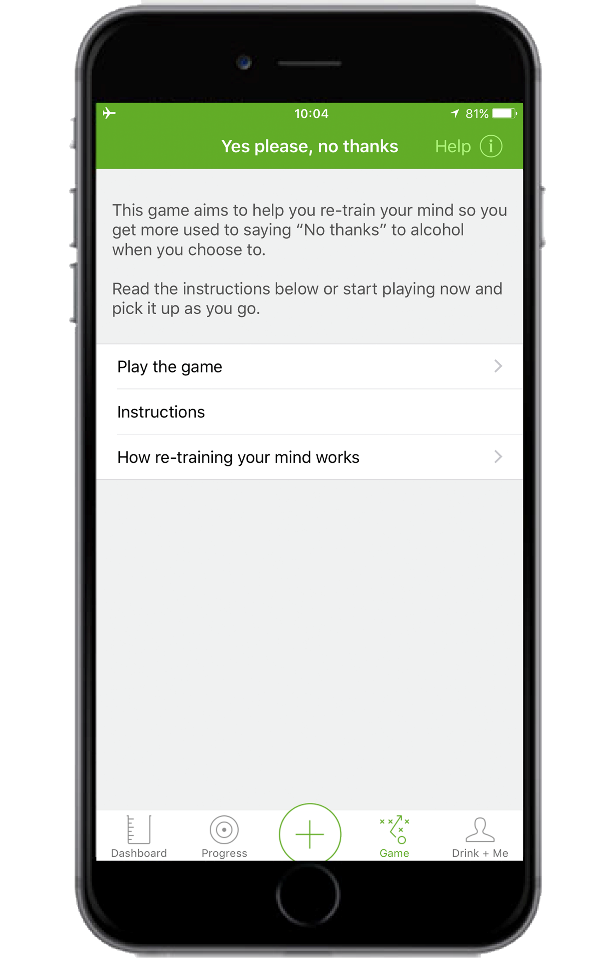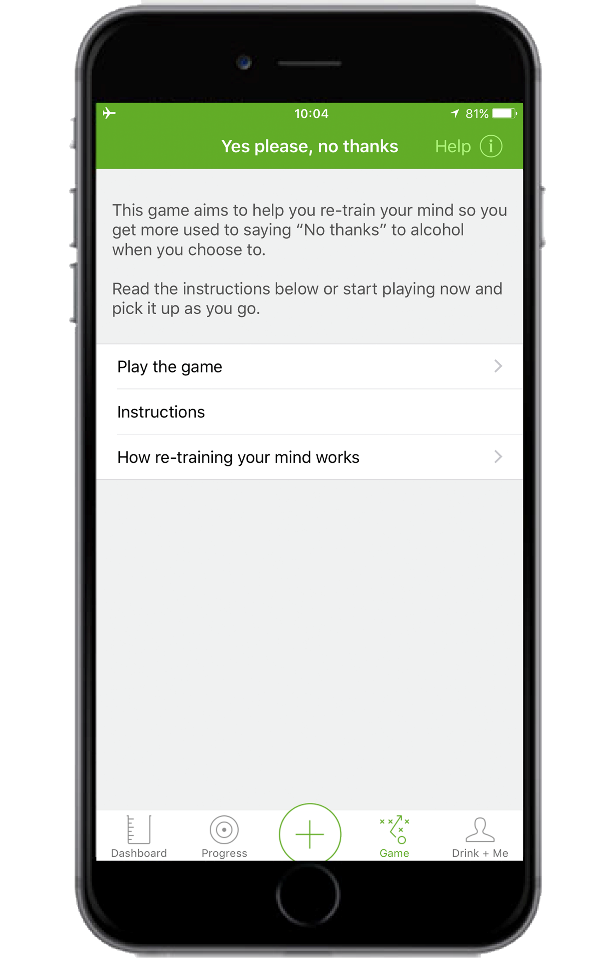a |  |  |
| --- | --- | --- |
| Menu screen for the “Yes please, No thanks” game for users in the control condition. |  |  |

### Self-monitoring and Feedback

#### Add Drinks panel


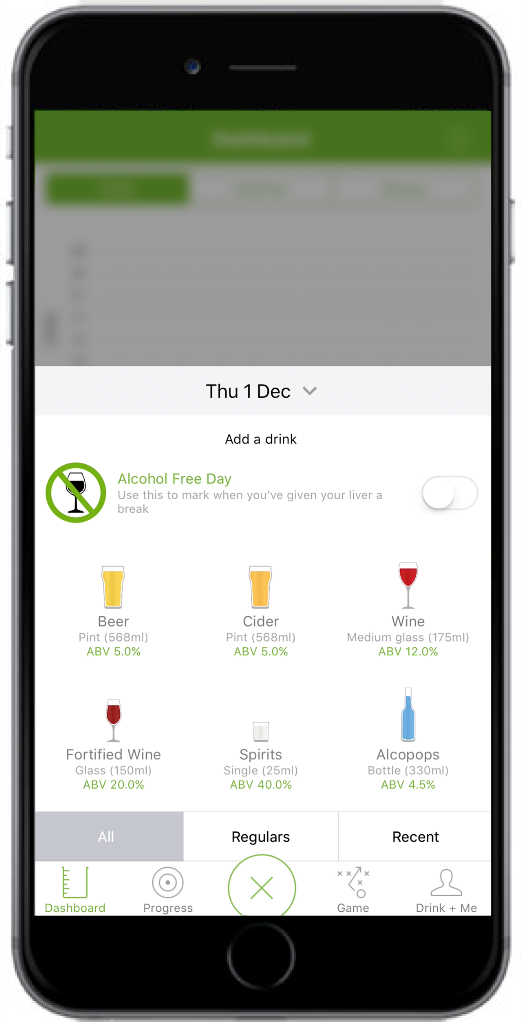


Links to Regulars and Recent. Regulars contains saved drinks, Recent contains a list of the most recently added drinks.

The ‘tab’ bar contained links to the four main sections of the app: Dashboard, Progress (Figure 1.21.1), Games (Cognitive Bias Re-training module) and Drink + Me (Identity module).

Users could choose between six types of drink.

Tapping the date displayed a calendar that allowed users to choose a particular date. The current date was displayed by default.

Users were able record an alcohol free day on the main Add Drinks panel.

This screen appeared when the circled X was tapped. This X was placed centrally and distinguished graphically to indicate its prominence to users and to make it easy to locate.

#### Add Drinks – Alcohol Free Day, Drink Details and Info button

| 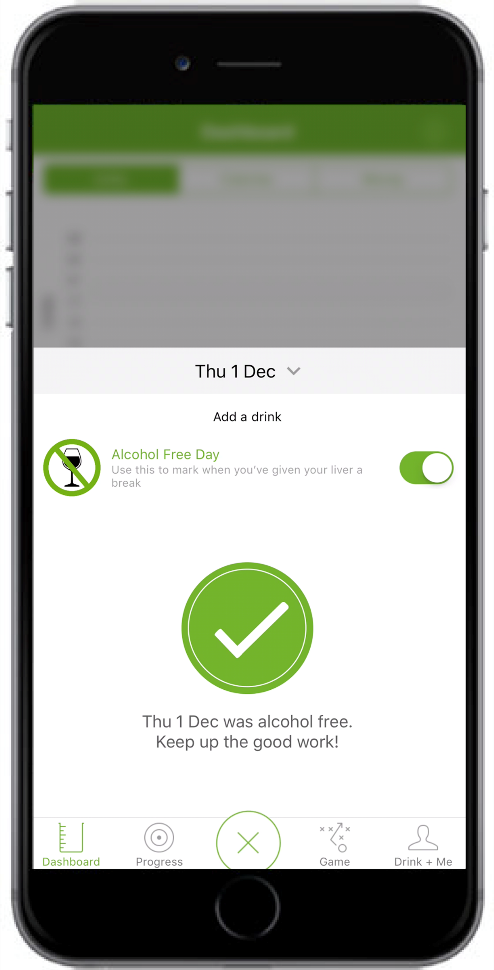a | 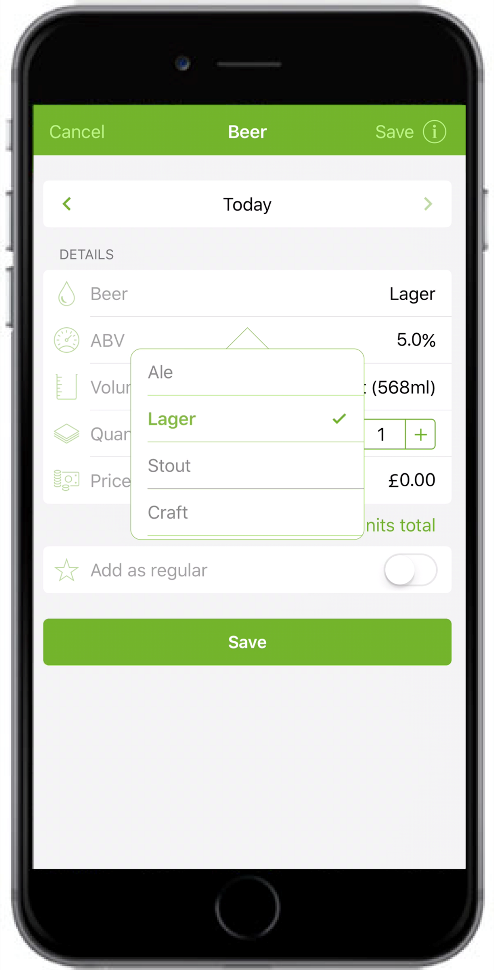b | 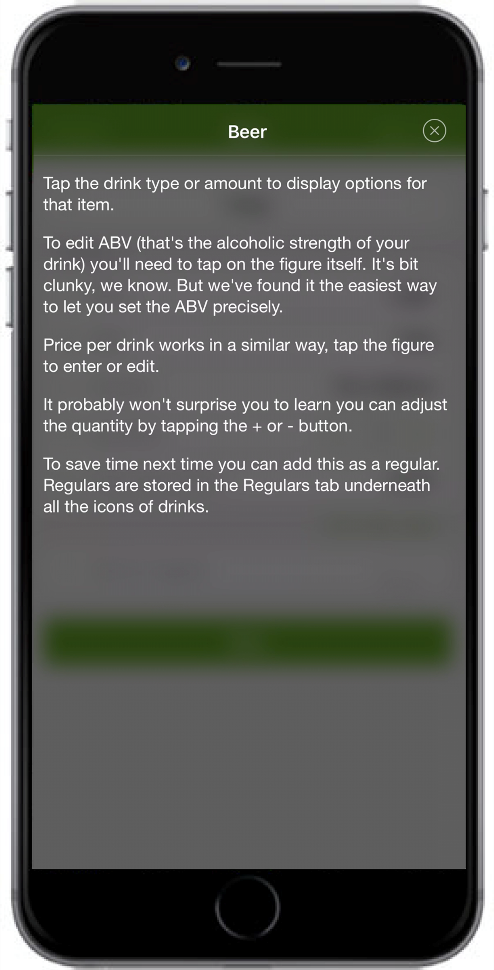c |
| --- | --- | --- |
| If a user marked a day as alcohol free a large green tick was displayed, a pleasing sound was played and text provided positive reinforcement | When a drink was chosen (beer in this case) the options for that drink were displayed. Users could also add the drink as a Regular | The information button (top right on the previous screen) helped participants understand how to enter data. Informal language was used |

####
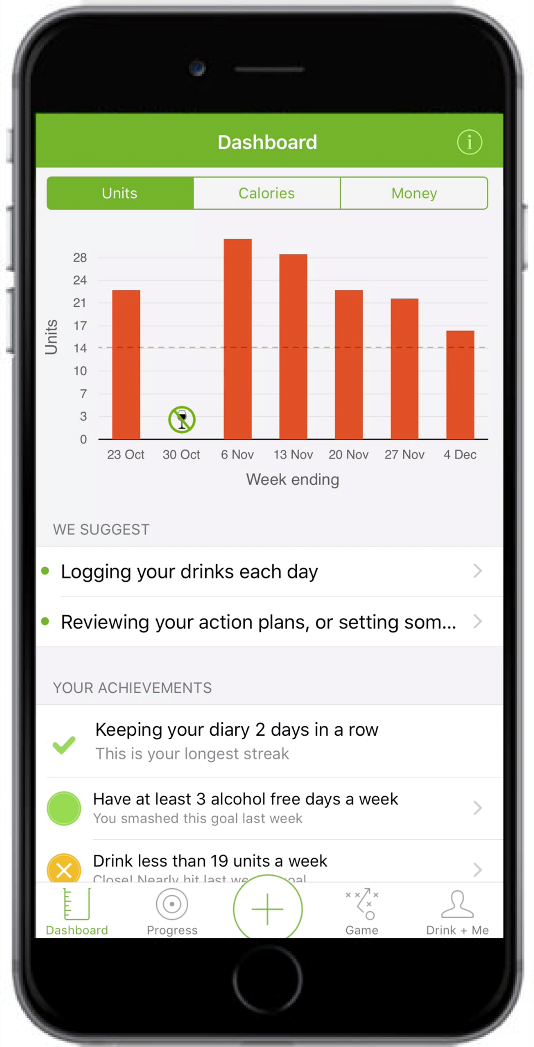
Dashboard top

Horizontal line indicates users unit goal

Icon indicates an alcohol free week.

‘Your achievements’ displayed information about the number of consecutive diary entries and summary of goal feedback for the last completed week, each one of which can be tapped for more info (Figure 1.21.4.9).

Links at the top provide access to feedback about Calories consumed, and Money spent, on alcohol

(Figure 1.21.4.5, a).

‘We suggest’ area prompted users to complete their daily diary. Every three days an additional message appeared prompting users to use another module of the app. Modules were promoted at random.

If all tasks were completed the text here changed to: Good work, you’re all done today.

Twenty-eight days after the user had downloaded the app a link titled ‘Please complete our questionnaire’ was added to this section.

Each of the bars which totals the units consumed for a week could be tapped to see a summary of consumption for the week (Figure 1.21.4.5, b).

####
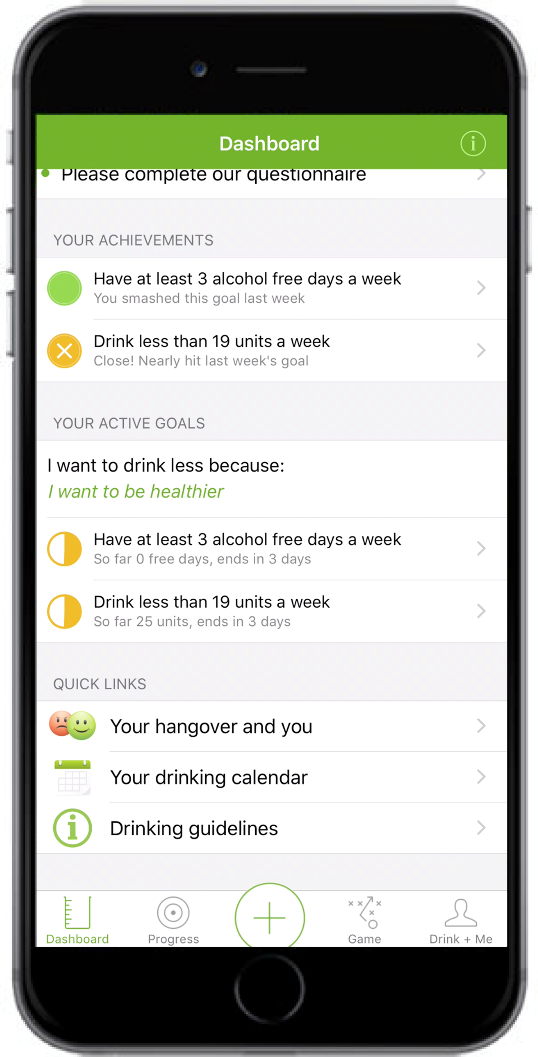
 Dashboard bottom

The green text under ‘I want to drink less because:’ displayed user entries to the first question asked in Goal setting (Figure 1.21.1, a)

‘Quick Links’ provided one-tap links to Mood Diary feedback (Your hangover and you, Figure 1.21.4.8, b), a user’s drinking calendar (Figure 1.21.4.10, a) and UK Government guidelines for alcohol consumption.

‘Your active goals’ also displayed information about progress toward goals for the current week. In the example here, the half-filled circle indicated the user was half-way through the current week, and the text below the goal title summarised how many alcohol free days they had had or units they had consumed to date.

#### Dashboard – Other graphs, Weekly summary and Info button

| 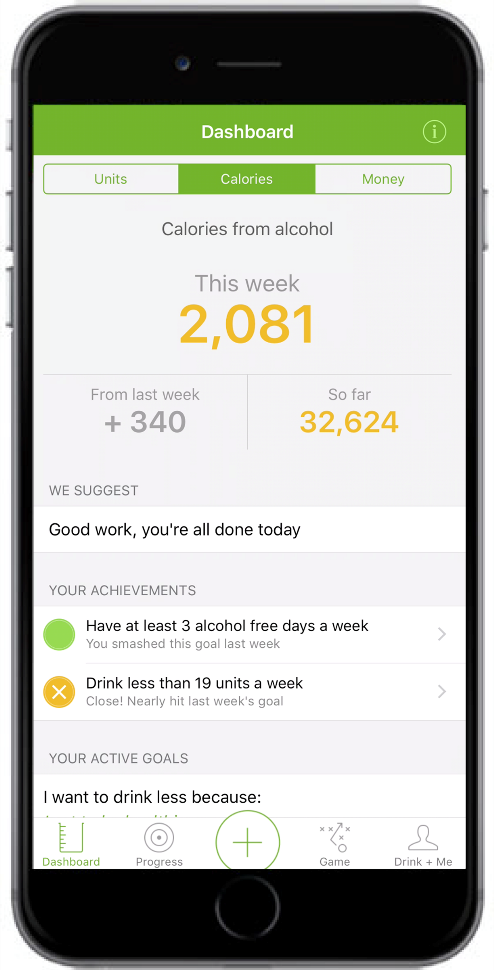a | 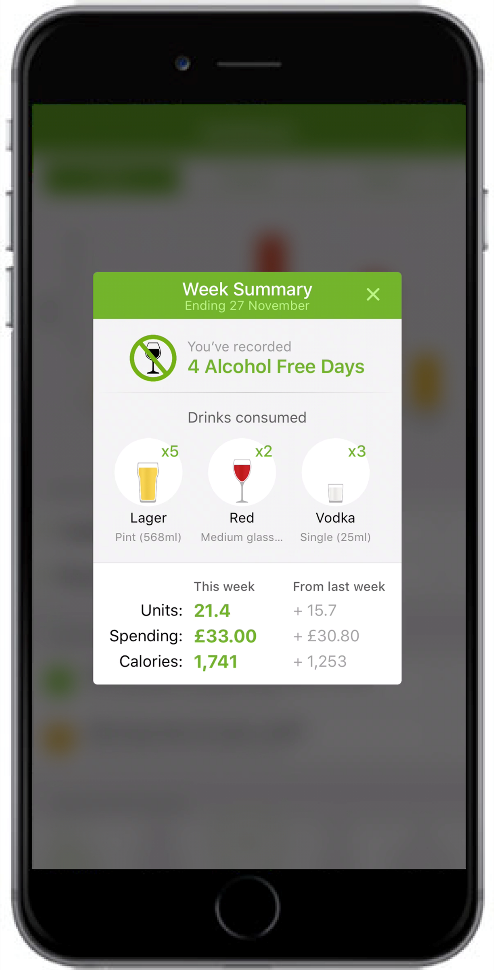b | 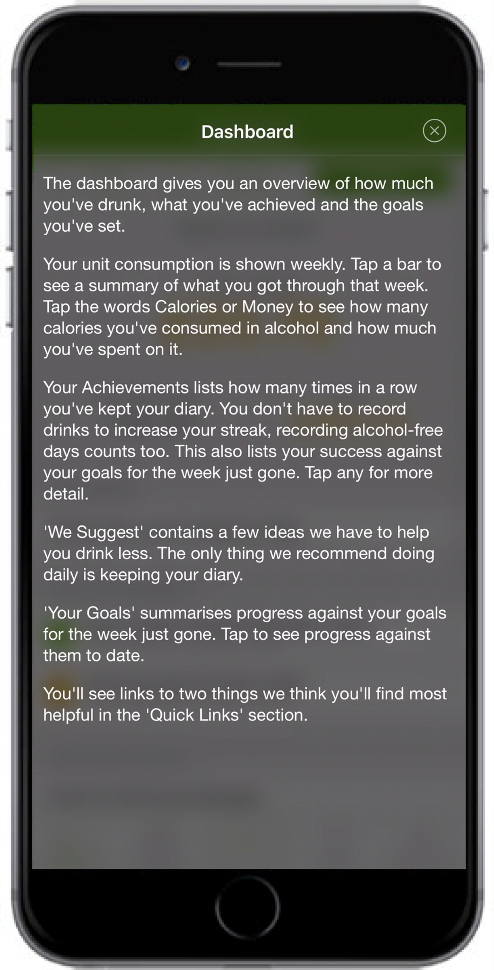c |
| --- | --- | --- |
| The Calories screen showed calories consumed from alcohol in the past week, to date, and how figures compared to the previous week | The weekly summary showed number of alcohol free days, totals of drinks consumed, and unit, spending and calorie totals and comparisons | The info button (top right of the dashboard) provided further info about this screen. Text for controls differed (Figure 1.22.4.12, b) |

#### Alerts to complete the drinking diary

| 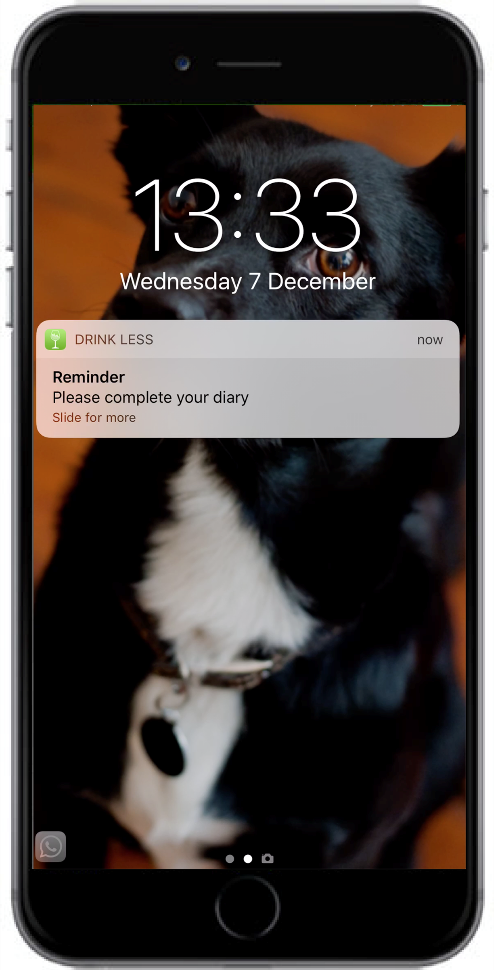a | 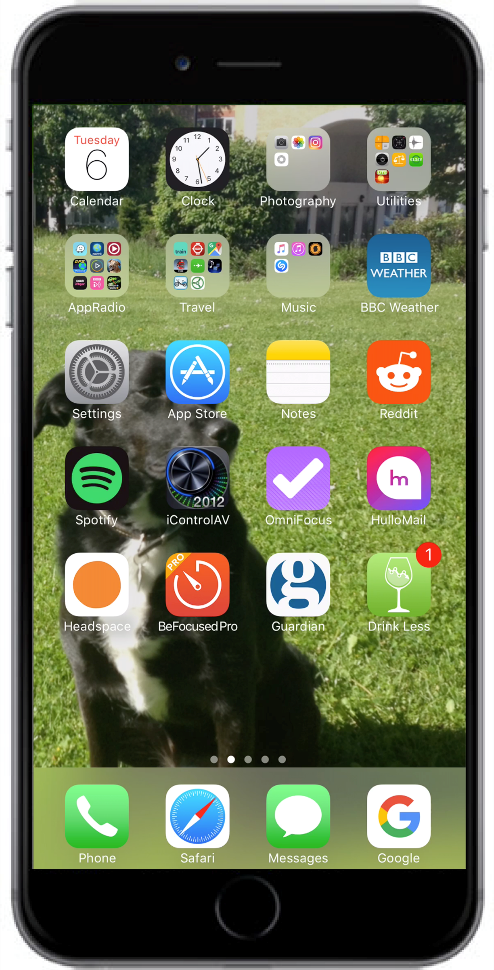b | 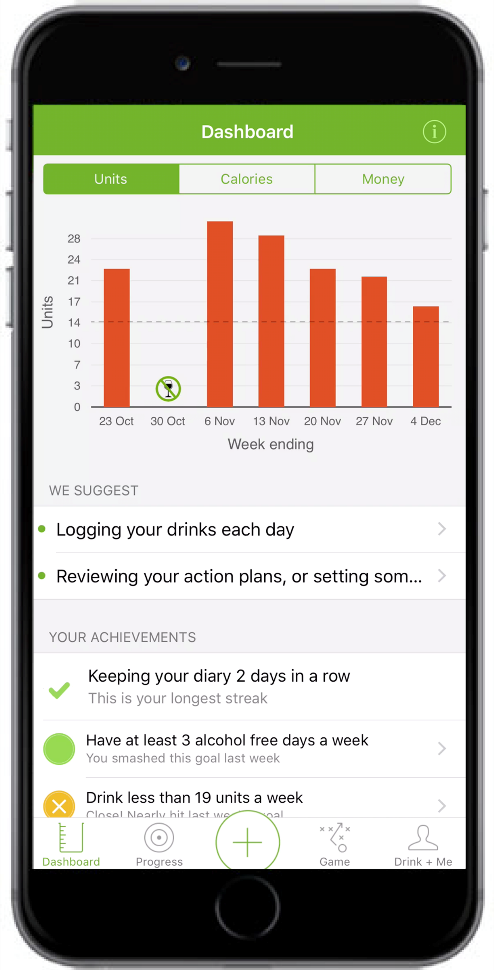c | |
| --- | --- | --- | --- |
| Prompt on the user’s home screen | Alert on the ‘badge app icon’ (5^th^ row, last app) | Alert on the Dashboard (In ‘We Suggest’) |  |

#### The Mood Diary questions

| 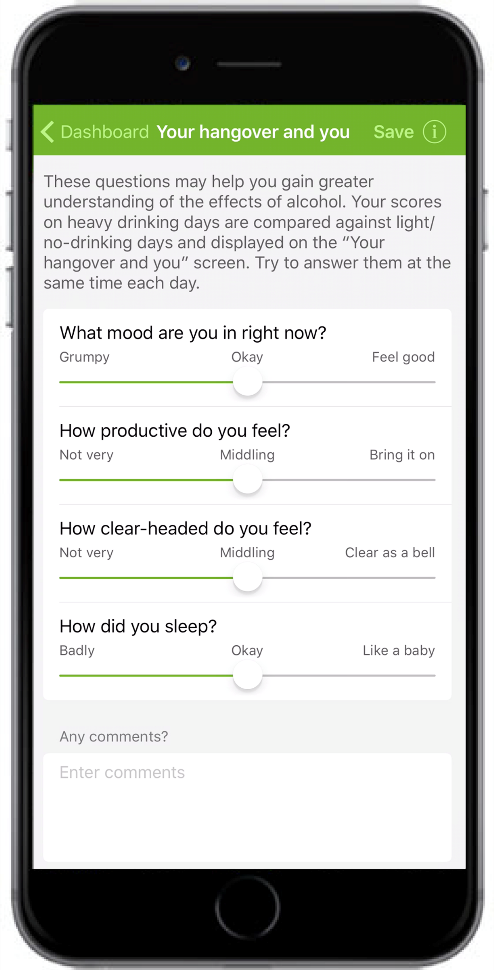a | 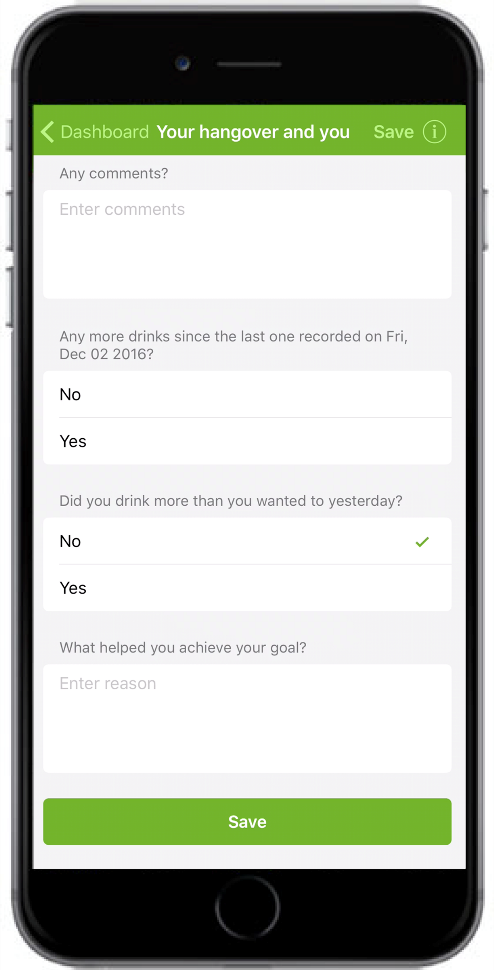b | 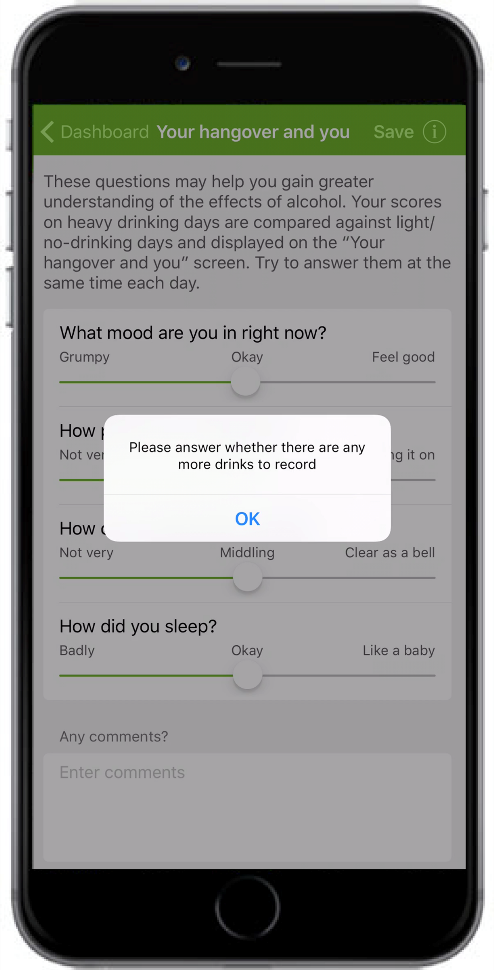c |
| --- | --- | --- |
| If users responded to the daily prompt to log their drinks they were presented with the Mood Diary questions, below which… | …were fields for comments, whether there were more drinks to record and whether the user drank more than they wanted to. | If users did not complete all the required fields an alert informed them which field needed their attention |

#### The Mood Diary info button and Mood Diary feedback

| 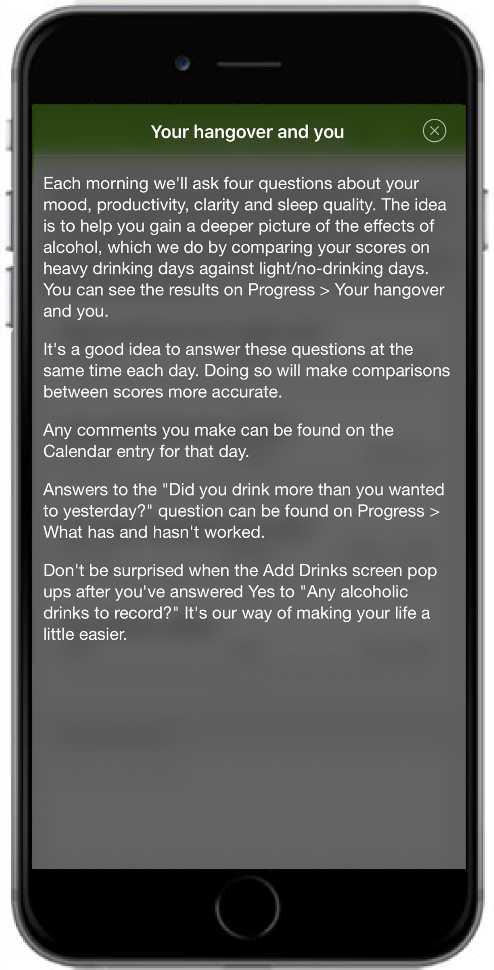a | 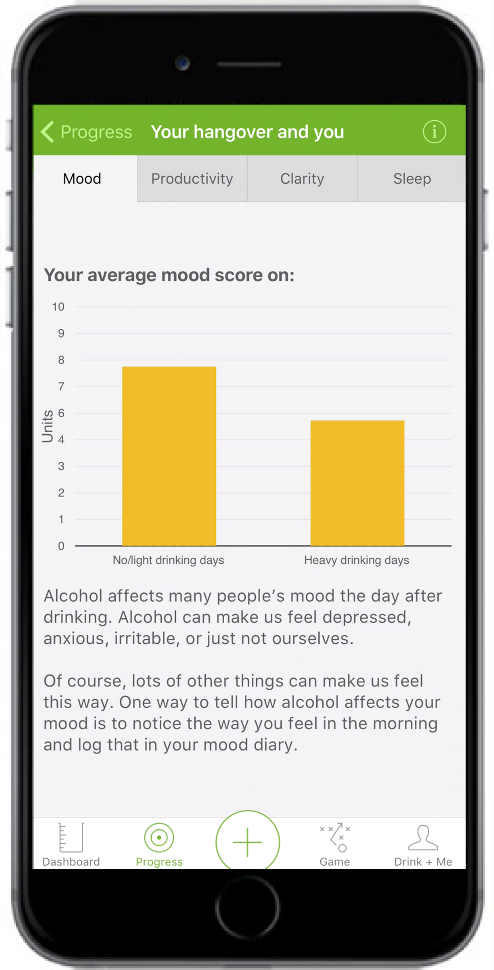b | 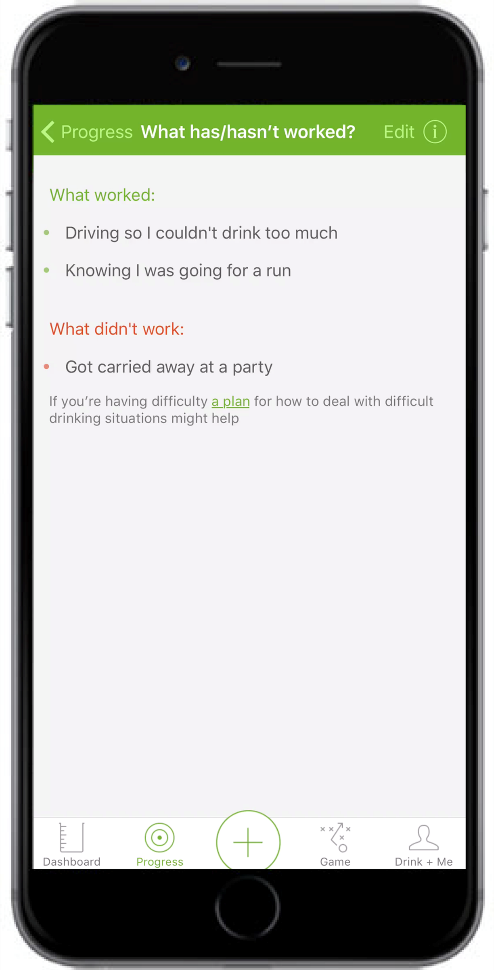c |
| --- | --- | --- |
| The info button explained the purpose of the Mood Diary and how to complete the form | Responses to the Mood Diary were displayed in ‘Your Hangover and You’, with text explaining why each measure might be affected by alcohol | Responses to the ‘Did you drink more than you wanted to yesterday?’ were displayed on Progress > ‘What has and hasn’t worked’ |

#### Goal feedback

| 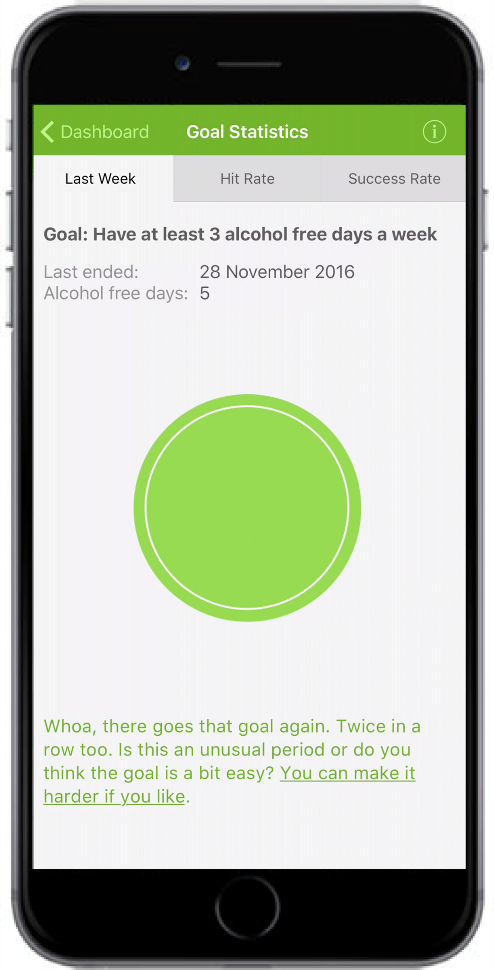a | 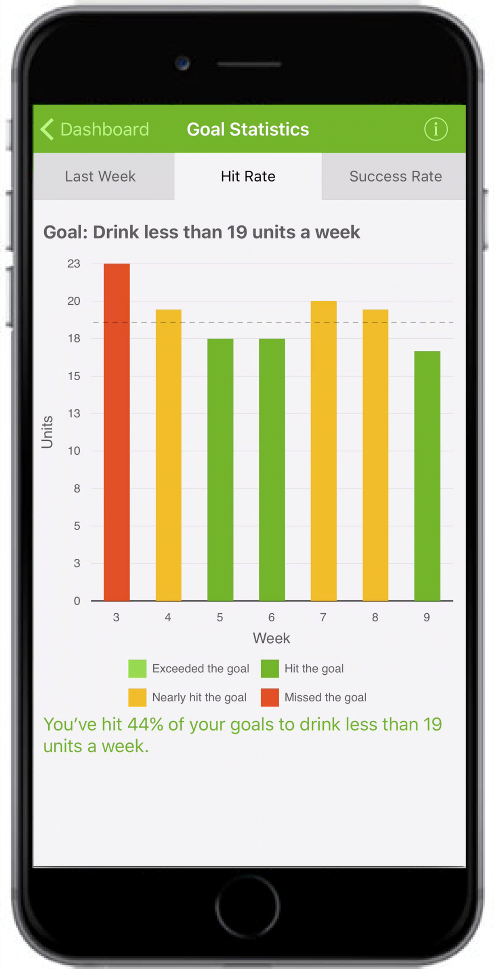b | 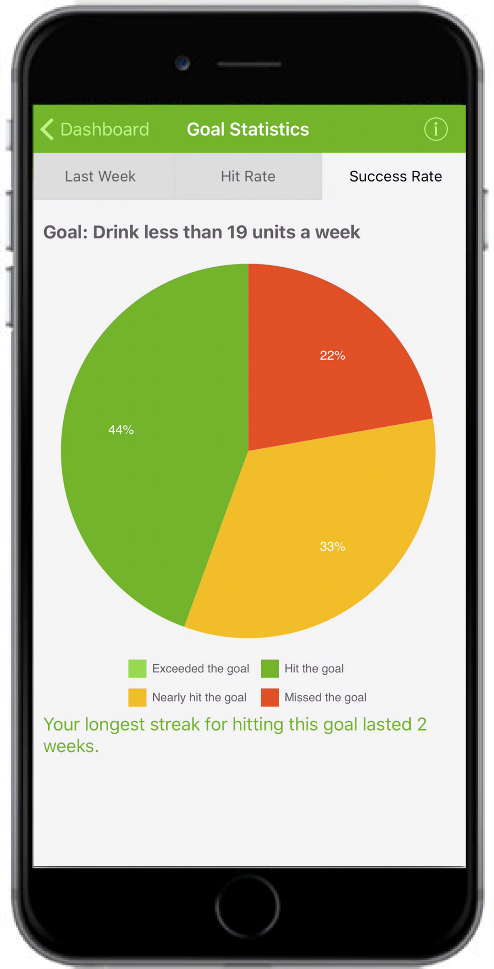c |
| --- | --- | --- |
| Info presented when summary goal feedback on the dashboard (Figure 1.21.4.4) or ‘Set and view goals’ screen (Figure 1.21.1, b) was tapped | The goal ‘Hit Rate’ screen provided an overview of how many times the goal had been exceeded, hit or missed since the app was downloaded | The goal ‘Success Rate’ screen provided a total of how many times the goal had been exceeded, hit or missed since the app was downloaded |

#### Calendar

| 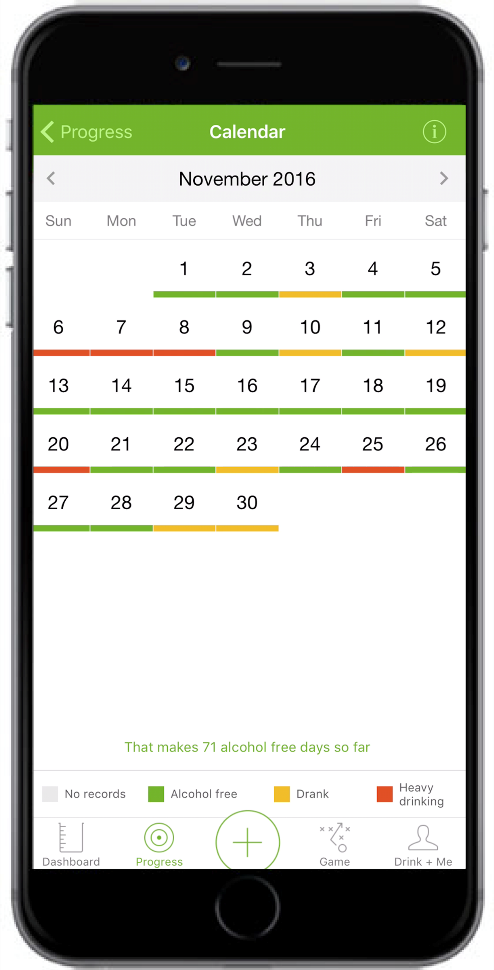a | 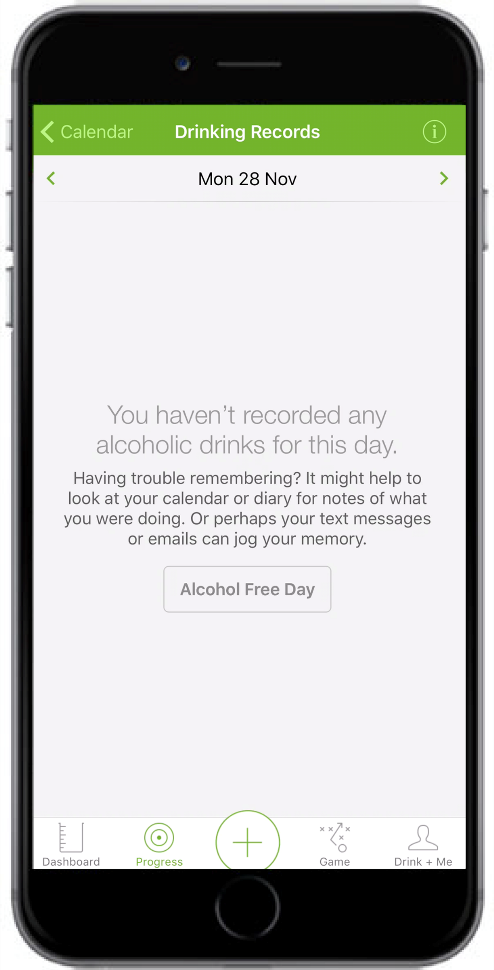b | 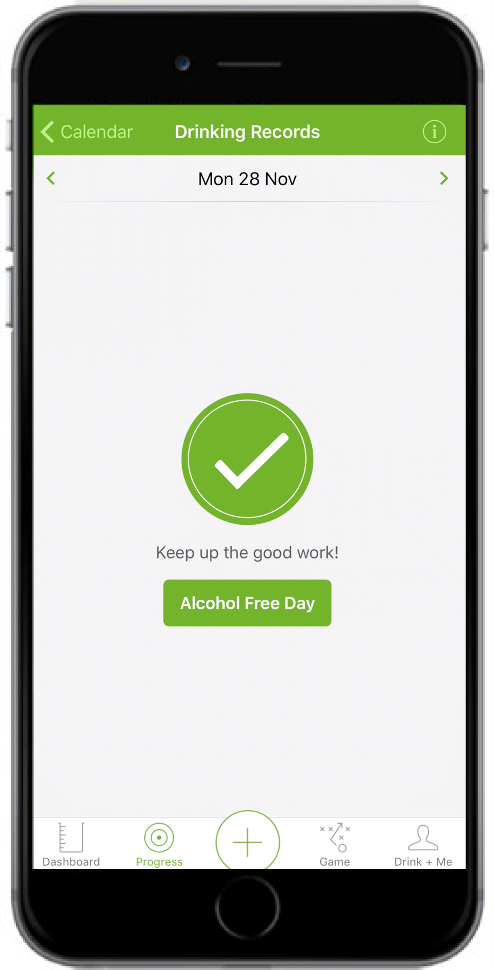c |
| --- | --- | --- |
| Coloured lines provided consumption feedback. Total number of alcohol free days was displayed toward the bottom of this screen | Timeline Followback procedure used to prompt people to recall past drinking behaviour. One touch button recorded an alcohol free day | If an alcohol free day was recorded a big tick was displayed alongside the text ‘Keep up the good work’ and a pleasing sound was played |

#### Calendar – Individual days

| 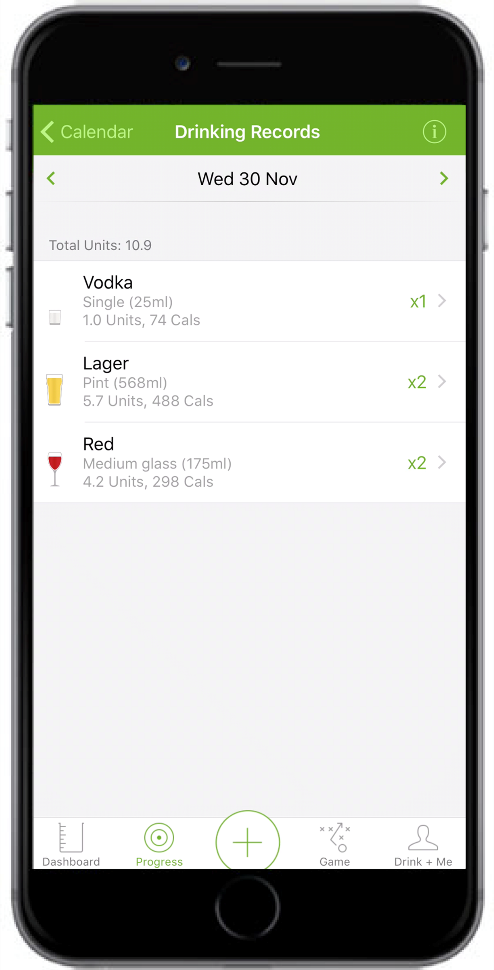a | 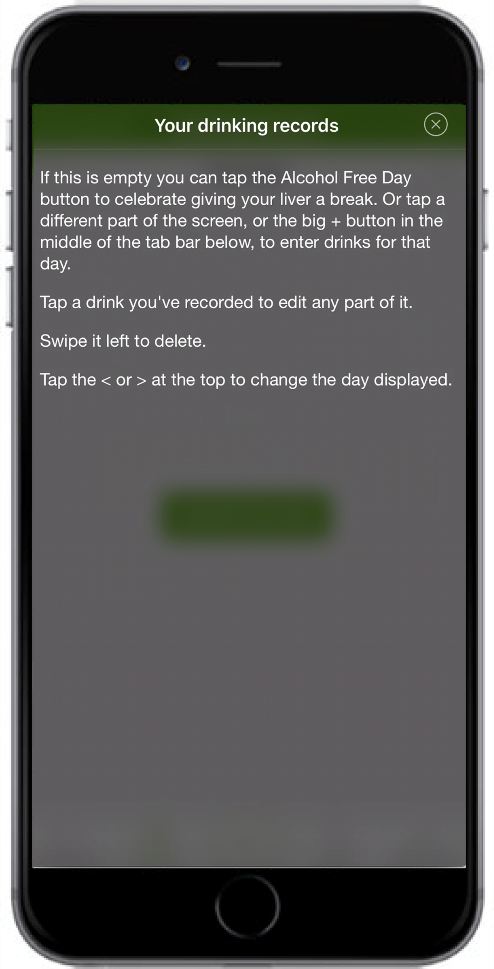b |  |
| --- | --- | --- |
| Tapping a day on the calendar displayed the total units consumed and details of each drink. These drinks could be edited or deleted | Text displayed when the info button was pressed helped users understand how to use this screen, for example how to edit and delete drinks |  |

#### Minimal version

| 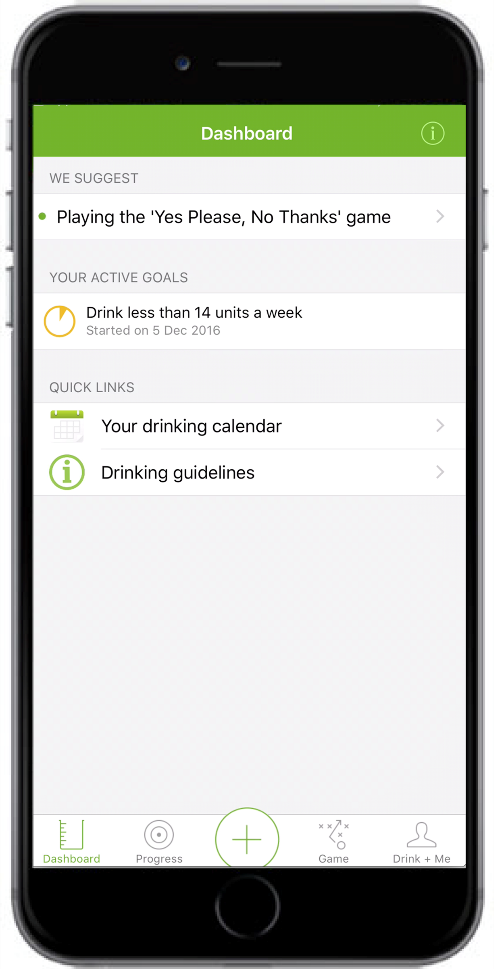a | 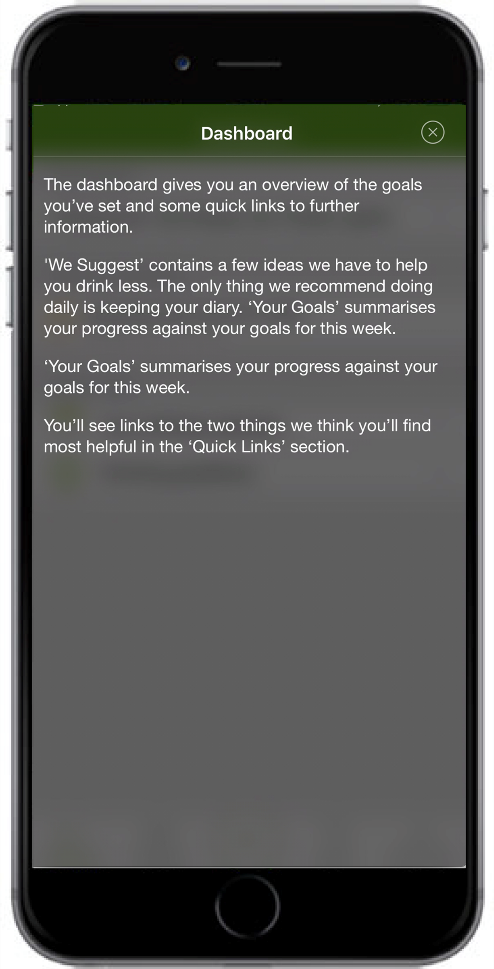b | 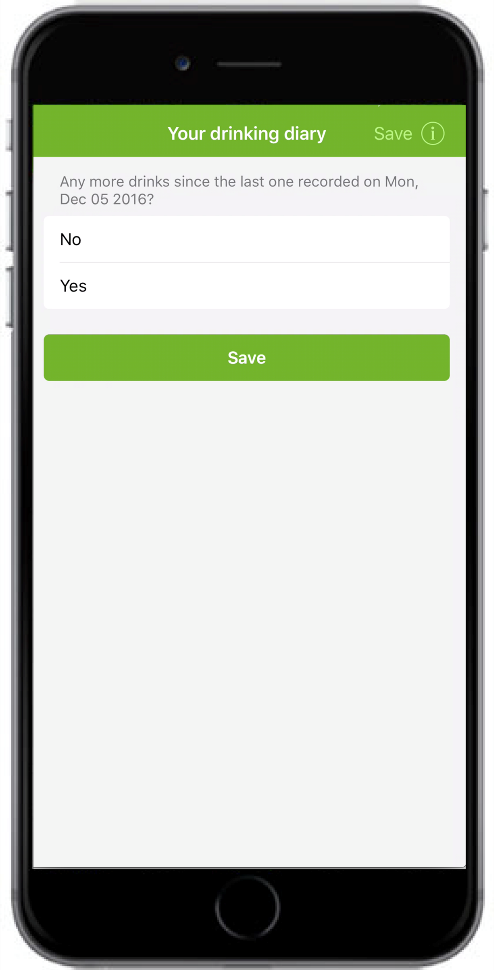c |
| --- | --- | --- |
| Control participants were given a plain dashboard with no graphs and no feedback. ‘Your active goals’ simply reminded users of their goal | The text displayed when the information button was pressed related to the screen that control participants saw (diff to Figure 1.21.4.5, c) | If users responded to the prompt to complete their daily diary they were only asked if they had drinks to record, no Mood Diary questions asked |

### Action Plans

#### Main screen and Why set an action plan

| 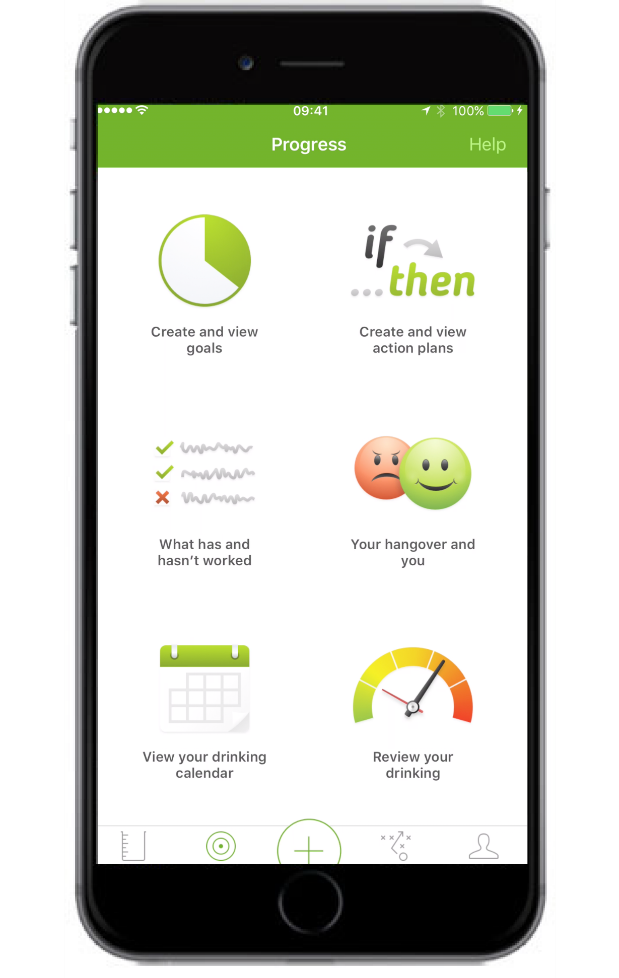a | 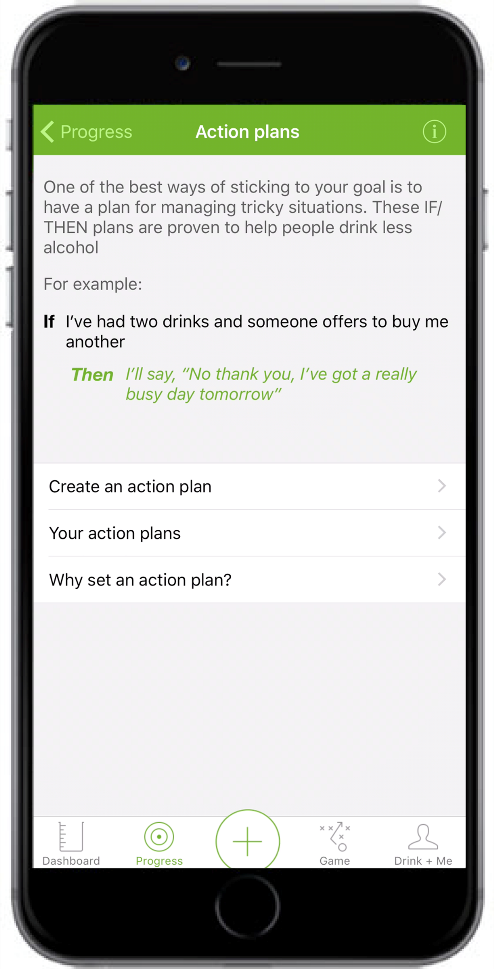­­­b | 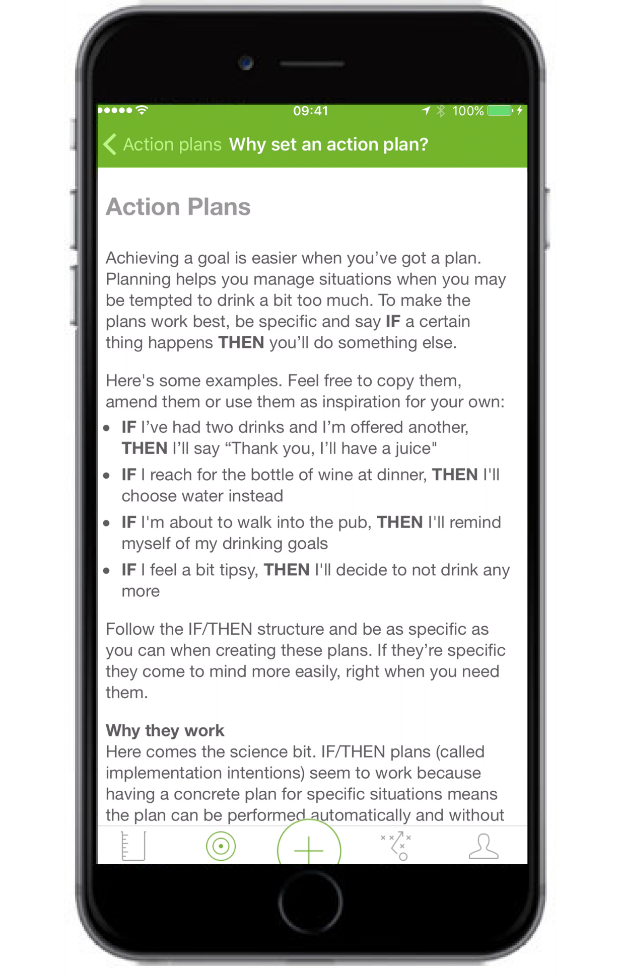c |
| --- | --- | --- |
| Action plans were accessed by tapping the top right icon on the Progress screen | The first Action Planning screen contained brief info about action plans, an example of an action plan and links to other screens | Information encouraging users to set an action plan and instructions about how they should be set was provided |

#### Create an action plan

| 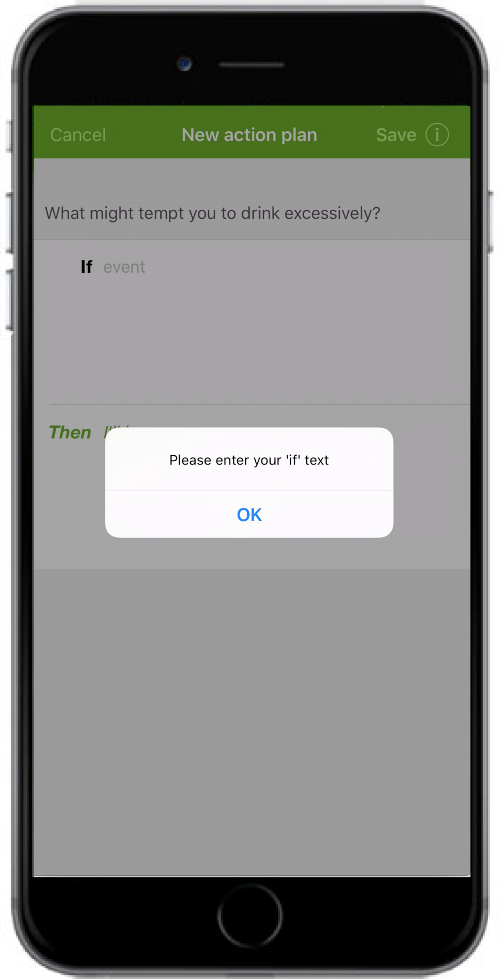a | 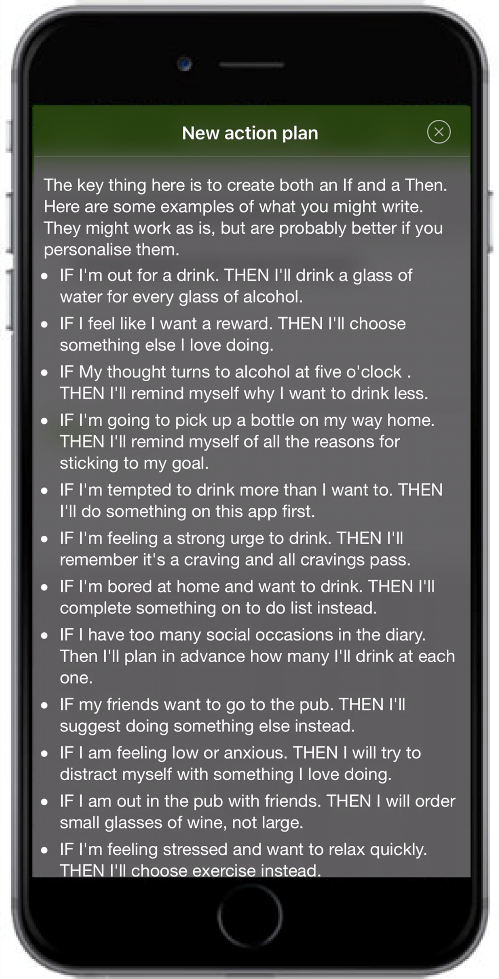b | 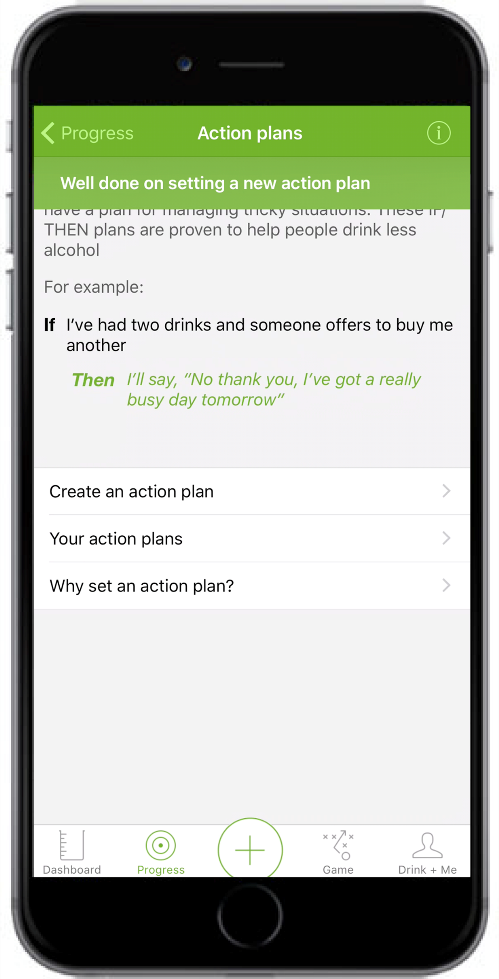c |
| --- | --- | --- |
| The “Create an action plan” screen contained separate fields for the If and Then components. An error message notified users of an empty field | Examples of Action Plans were provided. These were accessed via the info button on the ‘Create an action plan’ screen | Once an action plan had been set a confirmation message appeared at the top of the screen. A pleasing sound was also played |

#### Your action plans

| 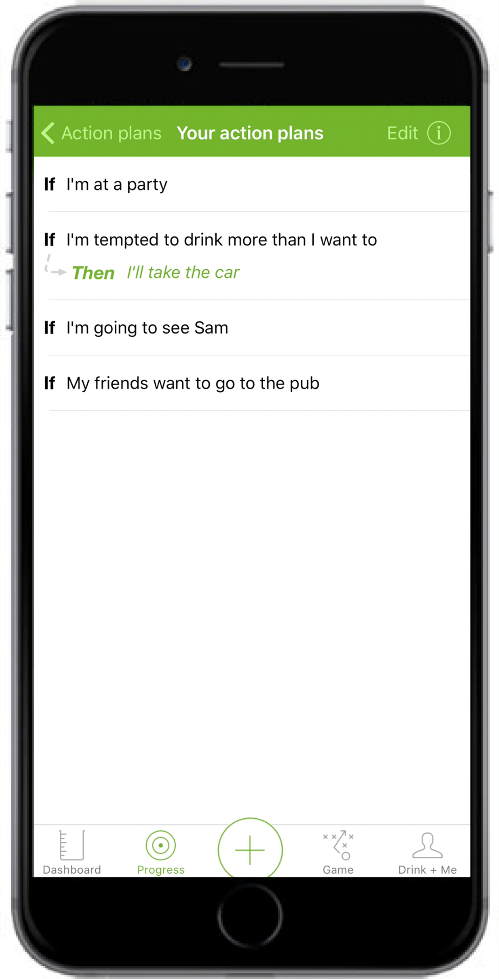a | 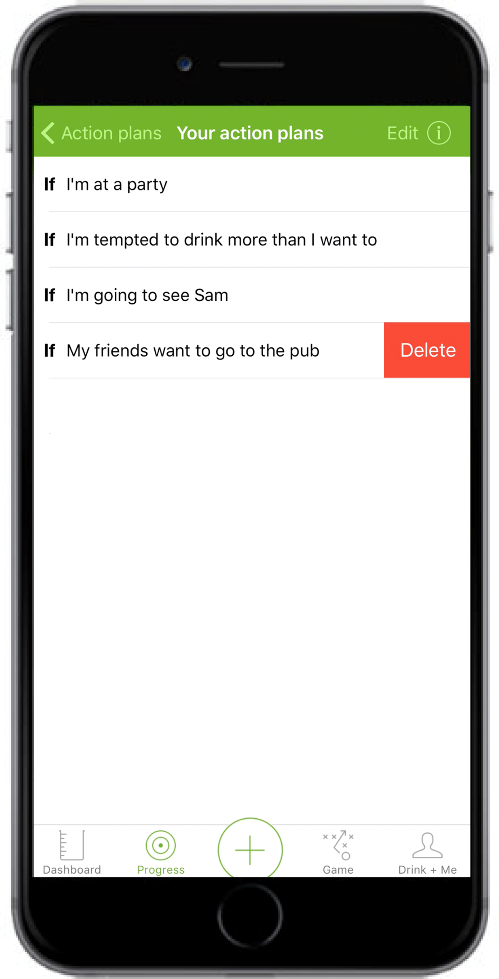b | 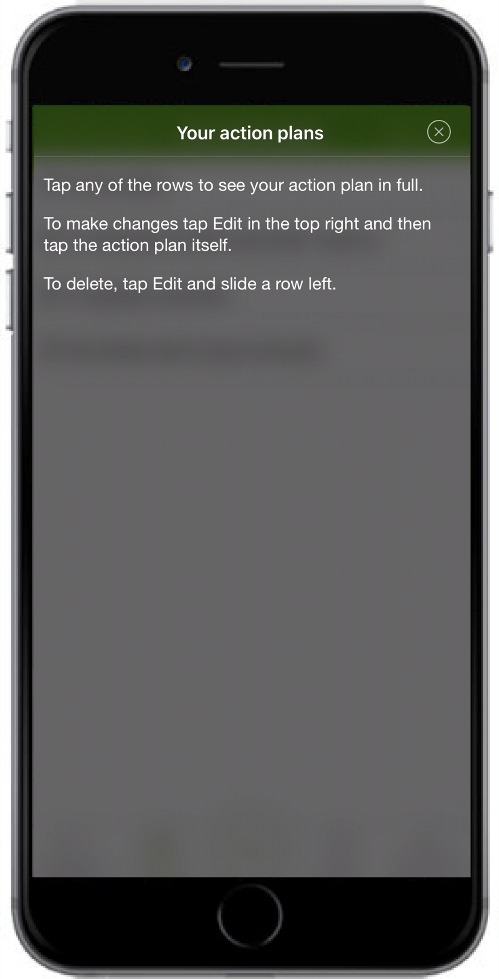c |
| --- | --- | --- |
| ‘Your action plans’ contains a list of all the action plans a user has set | Any action plan can be edited by tapping it, or deleted by swiping left. | If the info button in the top right of ‘Your action plans’ was tapped, tips appeared to explain how action plans could be viewed, edited or deleted |

#### Minimal version

| 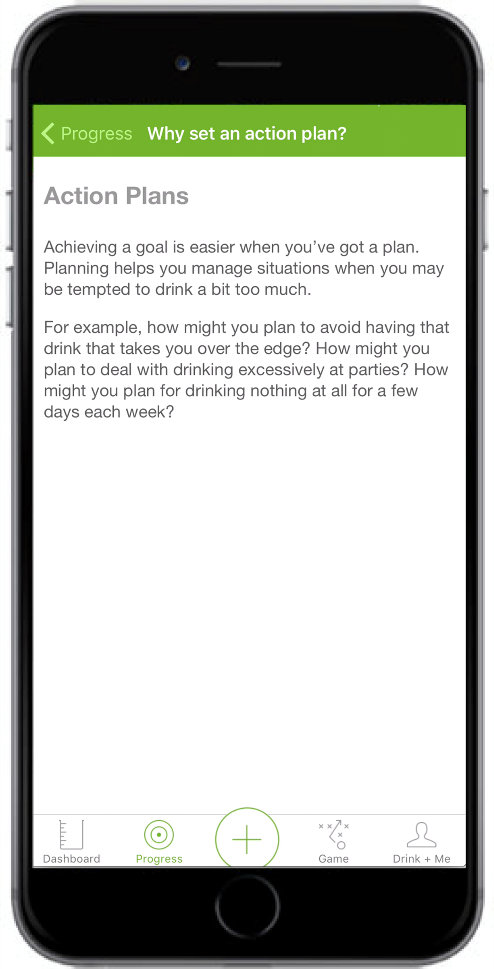a |  |  |
| --- | --- | --- |
| Control participants who tapped Create and View Action Plans (fig 1.21.6.6) were given only brief details about action plans |  |  |

### Identity Change (Drink + Me)

#### Main screen

| 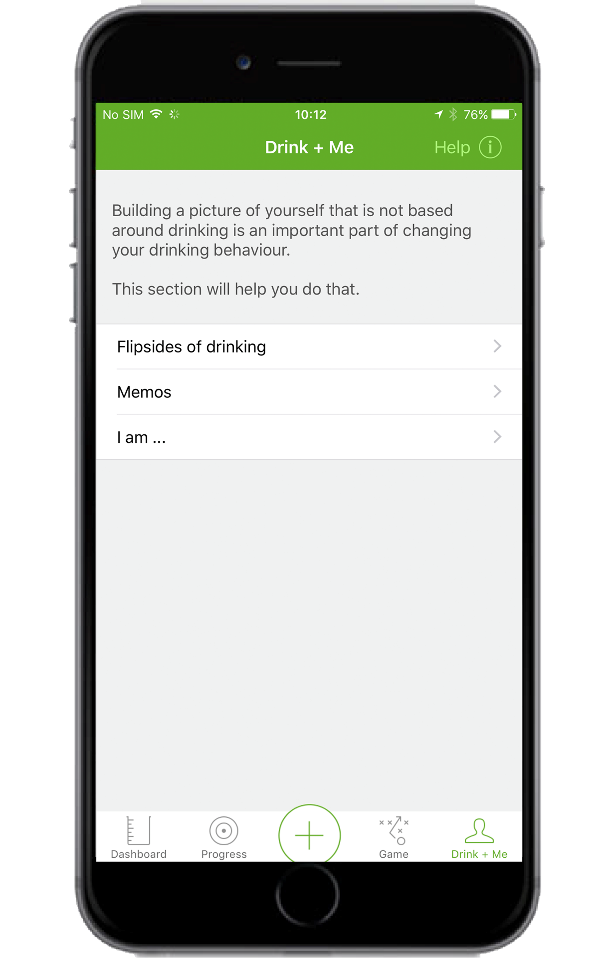a |  |  |
| --- | --- | --- |
| The main screen for ‘Drink + Me’ with a brief explanation for why the module should benefit the user and links to different sections. |  |  |

#### Flipsides of Drinking

| 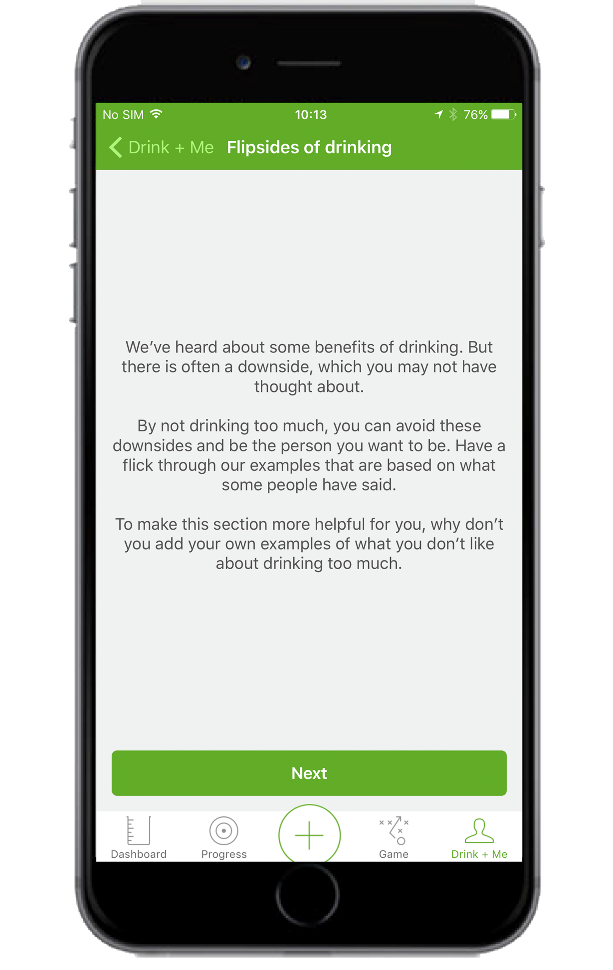a | 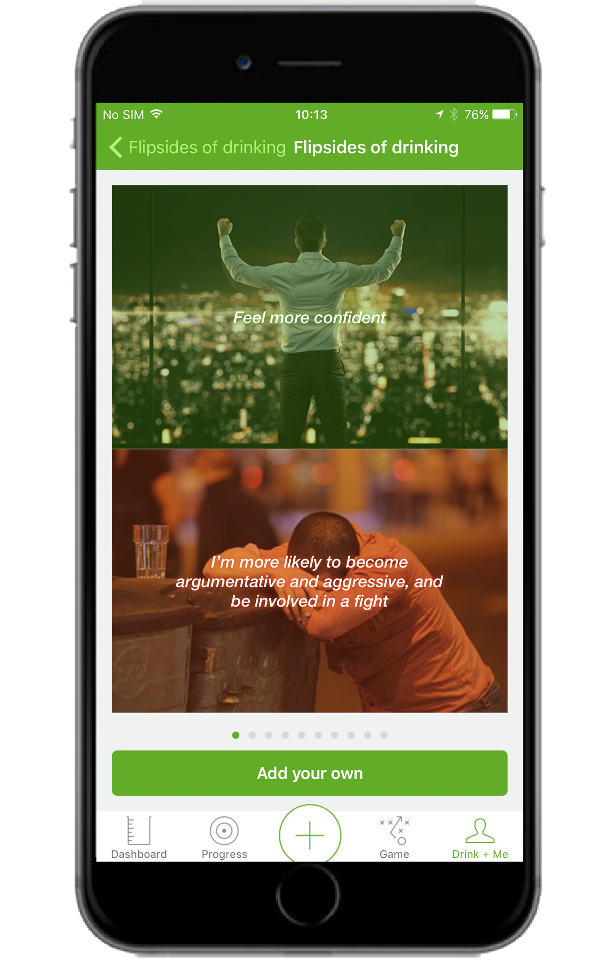b | 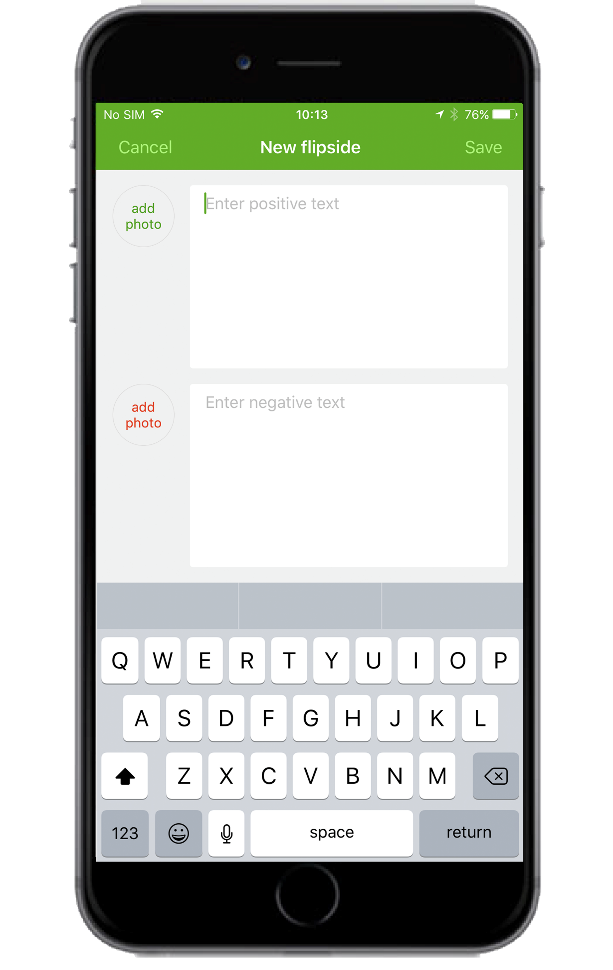c |
| --- | --- | --- |
| The first screen of the ‘Flipsides of drinking’ had some brief introductory text so the user knew what to expect. | The second screen had ten pairs of alcohol-related outcome expectancies that users could flick through. | Users could click ‘Add your own’ to then write their own flipside. |

#### Memos

| 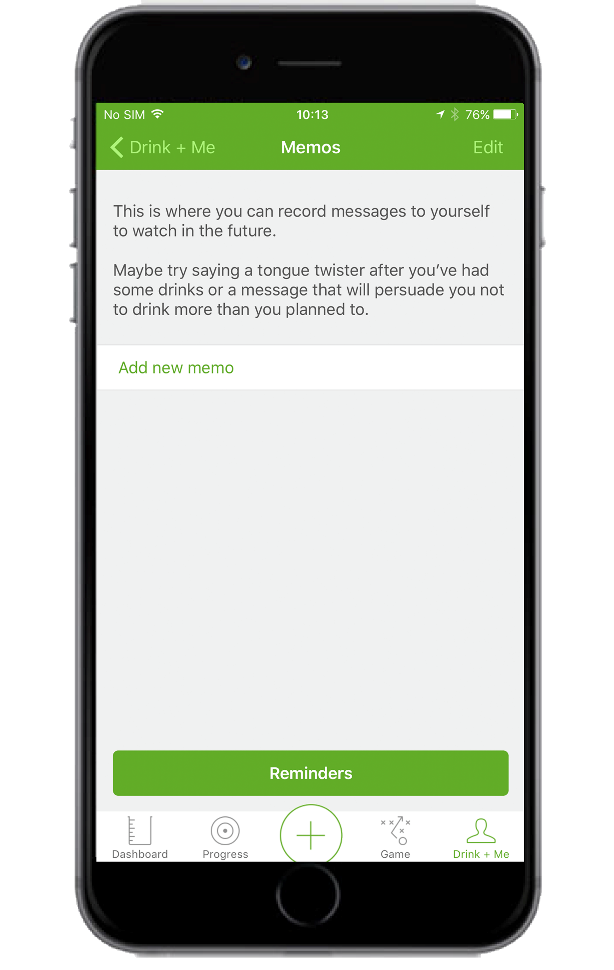a | 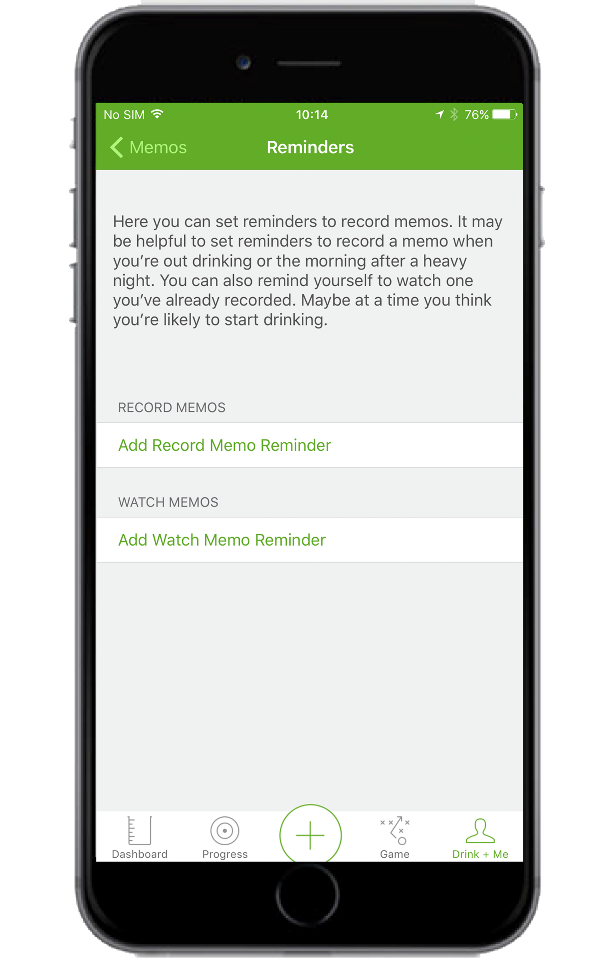b |  |
| --- | --- | --- |
| The Memos screen explained what the user could do within the section and gave an example of a potential memo to record. | The ‘Reminders’ button allowed users to add a reminder to either record a new memo or watch an existing one. |  |

#### I Am…

| a 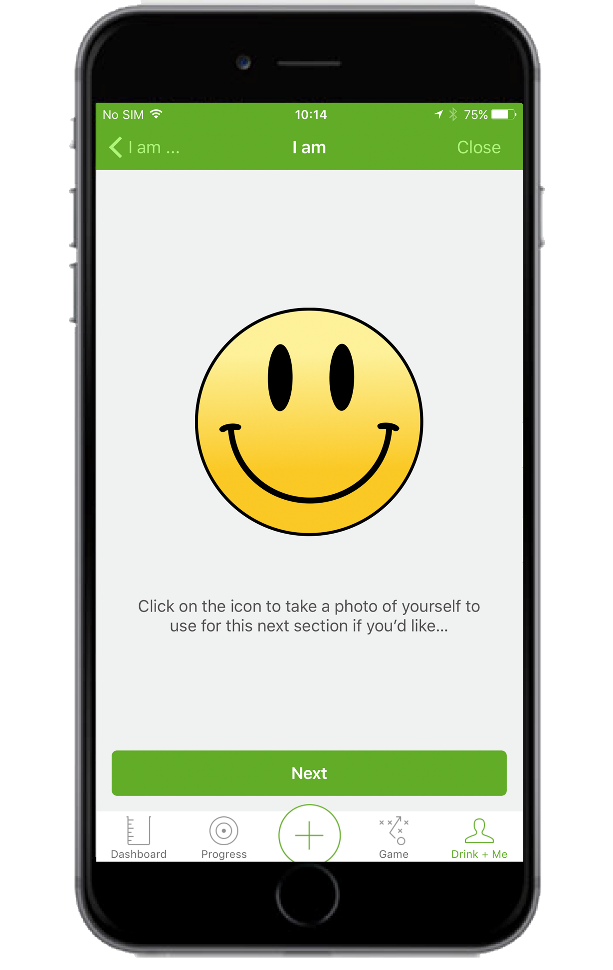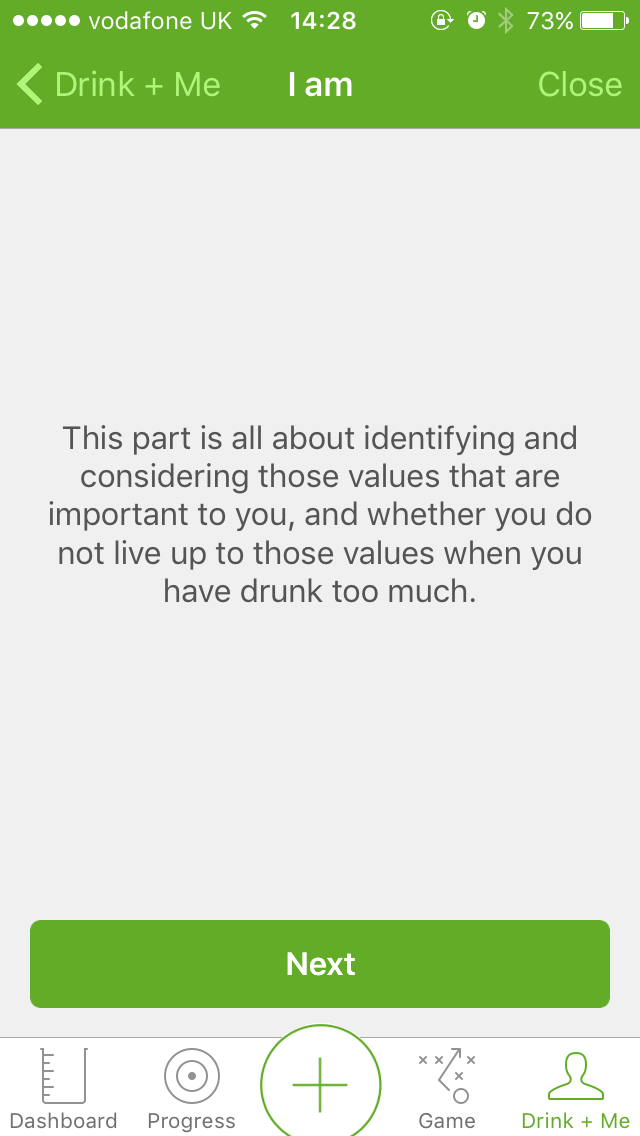 | 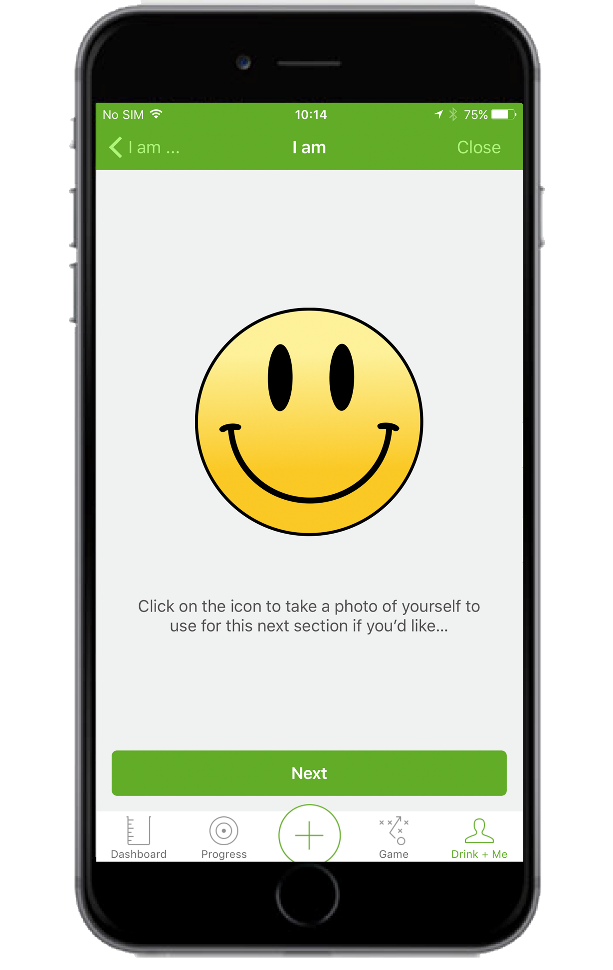b | 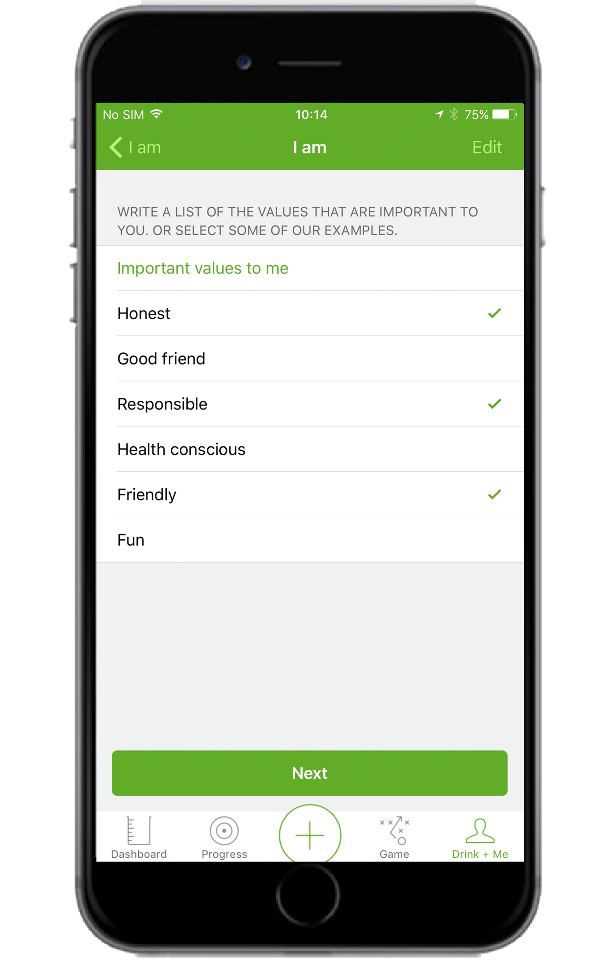c |
| --- | --- | --- |
| The first screen of this section explained to users the purpose of ‘I am…’. | Users could use a default image of a smiley face or tap on the smiley face to use their own photo. | Users were asked to list their values of importance or select some from a list of examples. |

#### I Am…

| 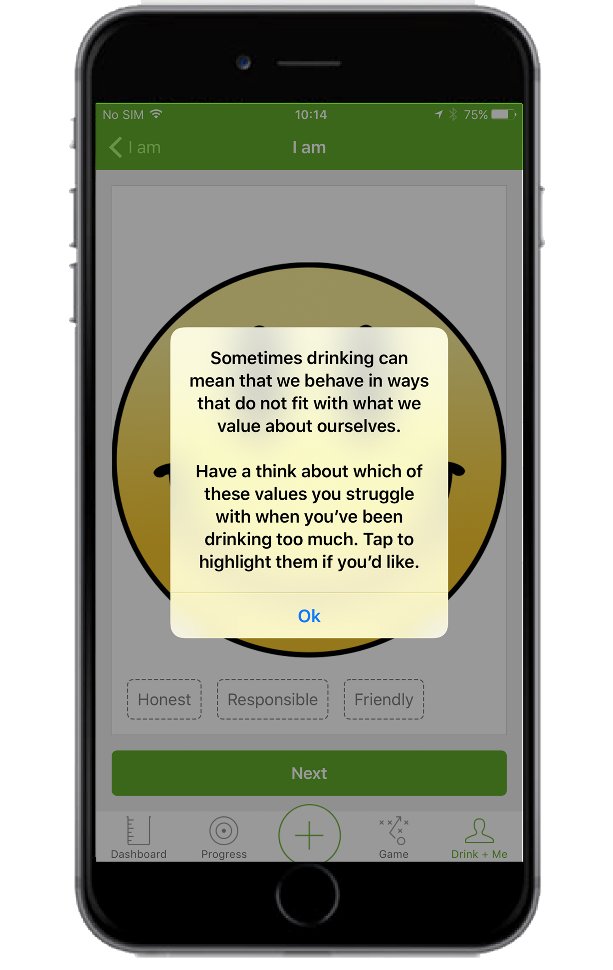a | 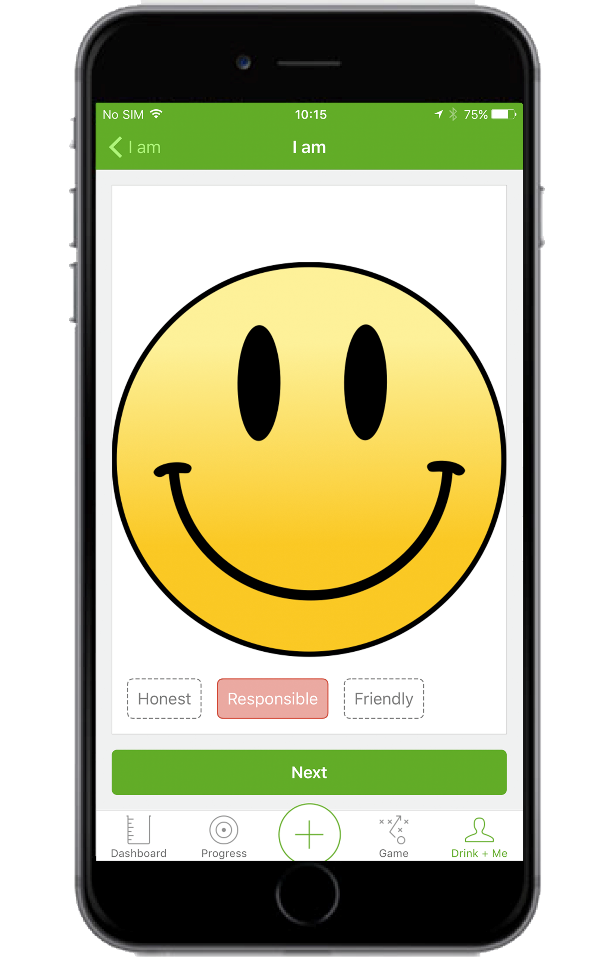b | 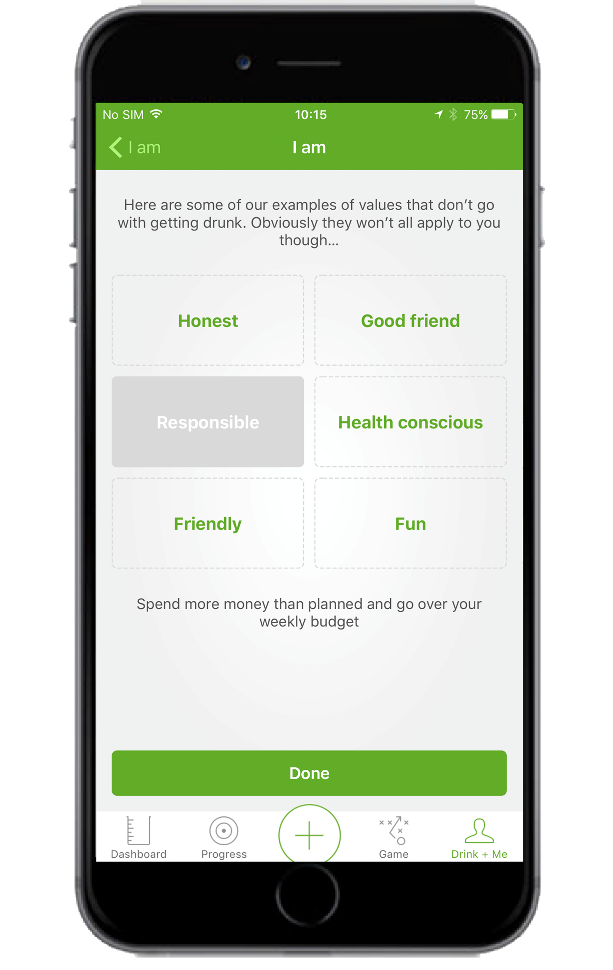c |
| --- | --- | --- |
| A pop-up appeared asking the user to consider which of their values they struggle with when they had been drinking too much. | The user could tap any of these values to select them and this value would then be highlighted in red. | Examples of common values of importance ot people and how drinking behaviour could be inconsistent with those values. |

#### Minimal version

| a 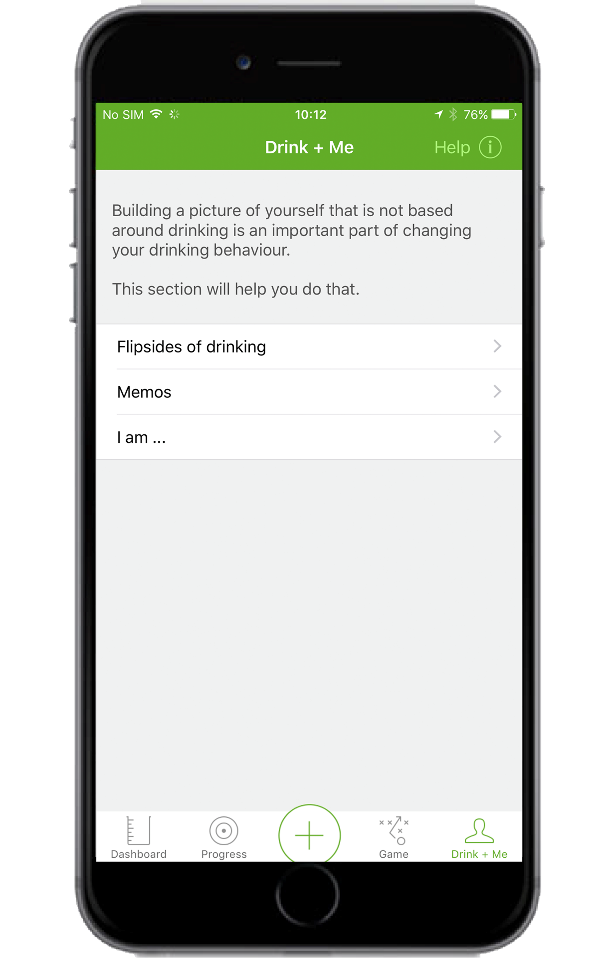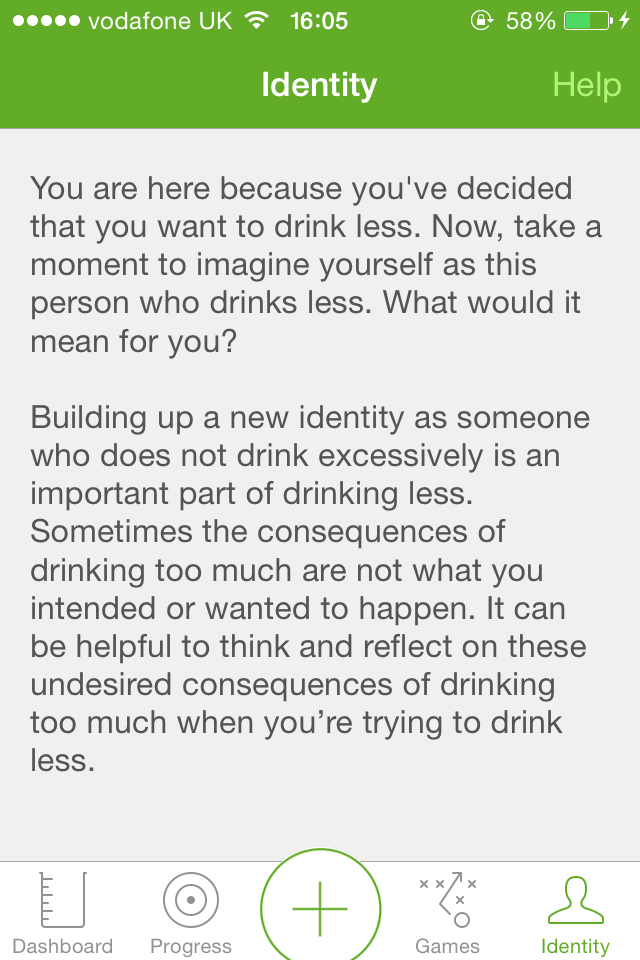 |  |  |
| --- | --- | --- |
|  |  |  |

## Other features

###
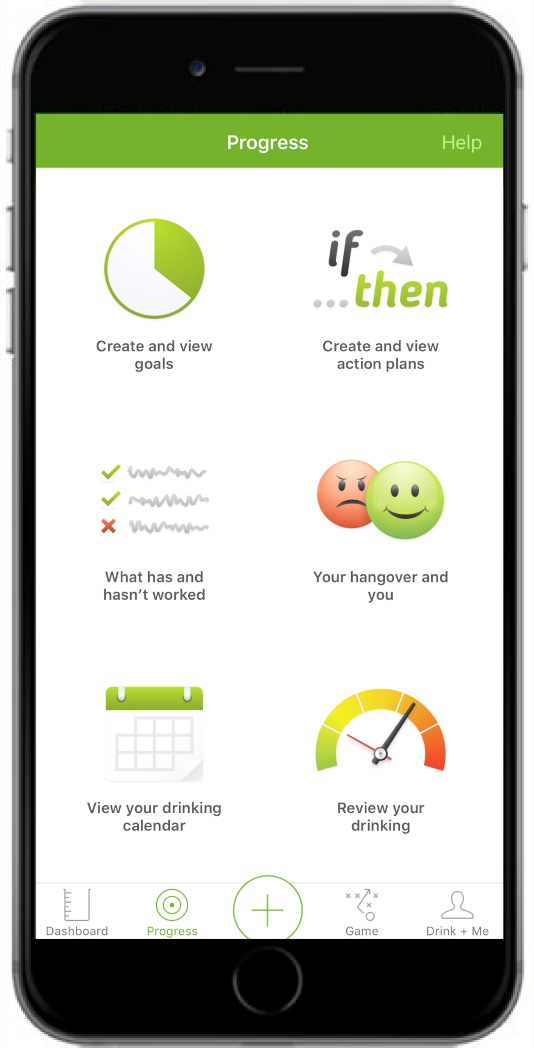
Progress screen

The Progress screen displayed links to several elements of the app

Review Normative Feedback information (Figures 1.21.2.2 & 1.21.2.3)

Feedback about entries to the Mood Diary (Figure 1.21.4.8, b)

Create and view action plans (Figure 1.21.5.1, b)

Set new goals or get feedback on the goals already set (Figure 1.21.1 & 1.21.4.9)

Summary feedback about drinking and links to edit entries (Figure 1.21.4.8, c)

User entries about the things that had helped or hindered their drinking reduction goals (Figure 1.11.15, c)

### Registration

| 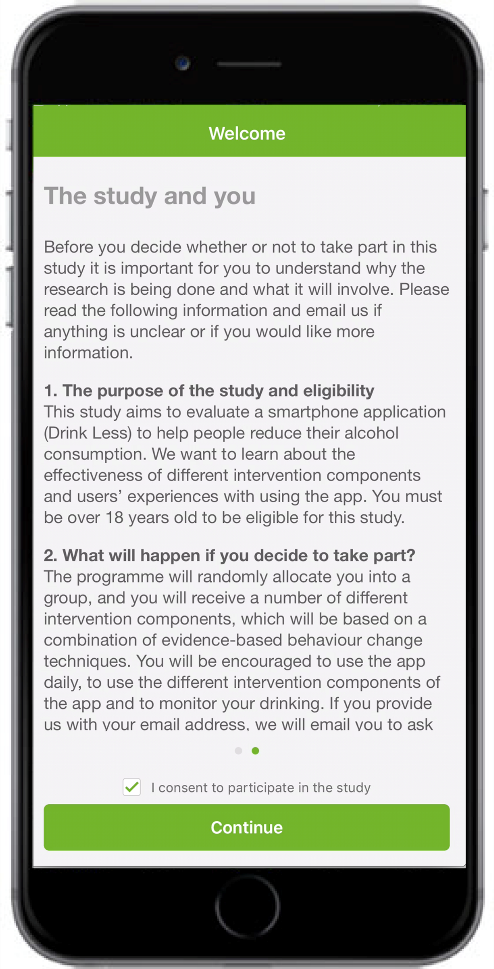a | 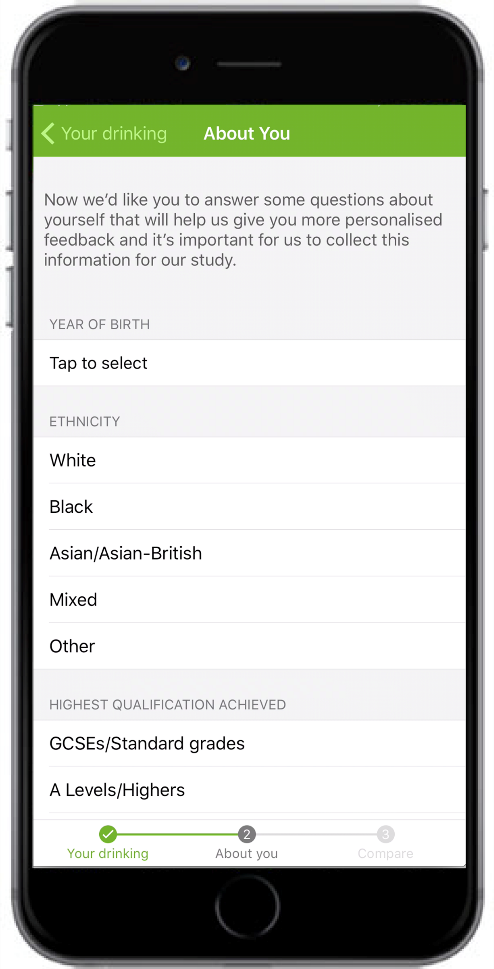b | 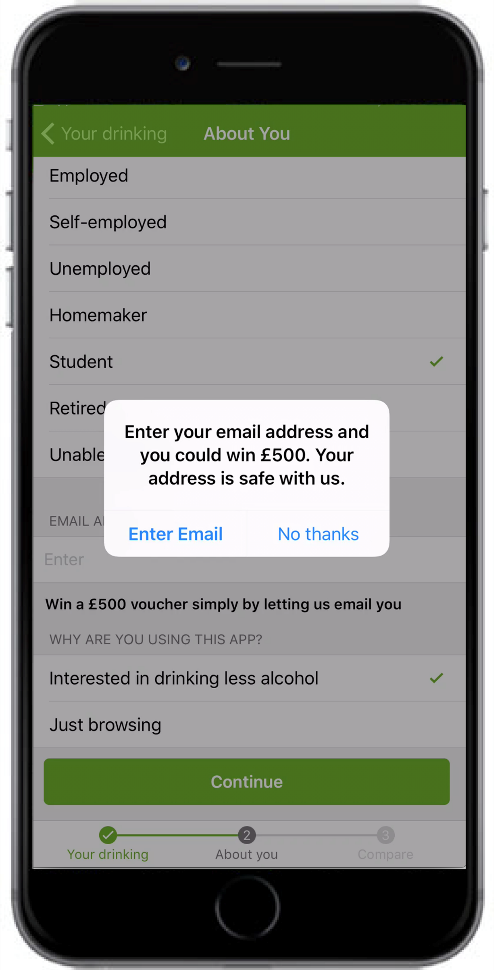c |
| --- | --- | --- |
| The information to consent screen provided details about the study. Users needed to consent to participate to be able to use the app | Text at the top of baseline questionnaires explained why the info was needed. An indicator at the bottom showed how many steps to go | Users were prompted to complete the email field if they originally left it blank. Choosing No Thanks allowed users to continue |

| 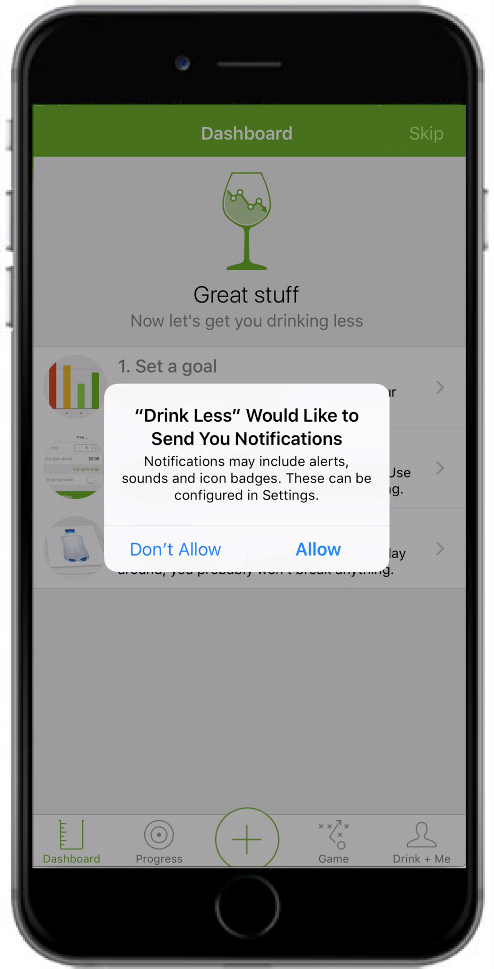d | 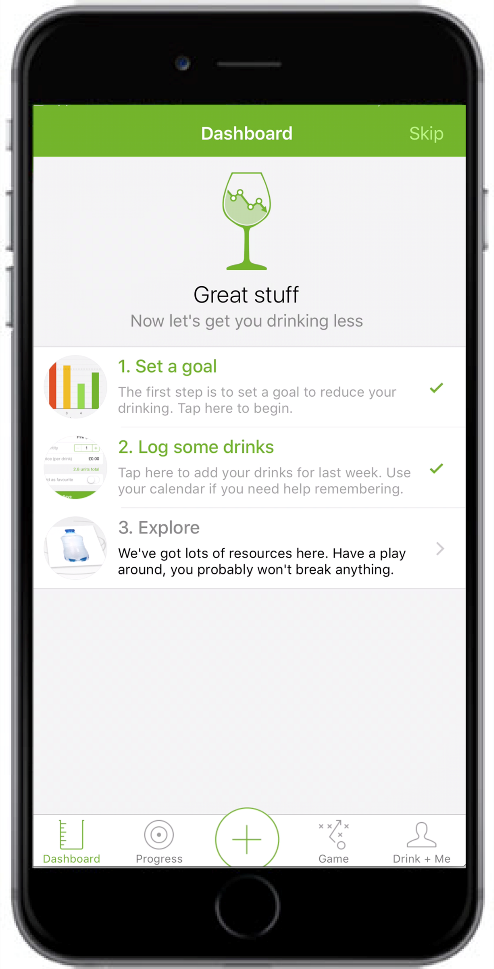e | 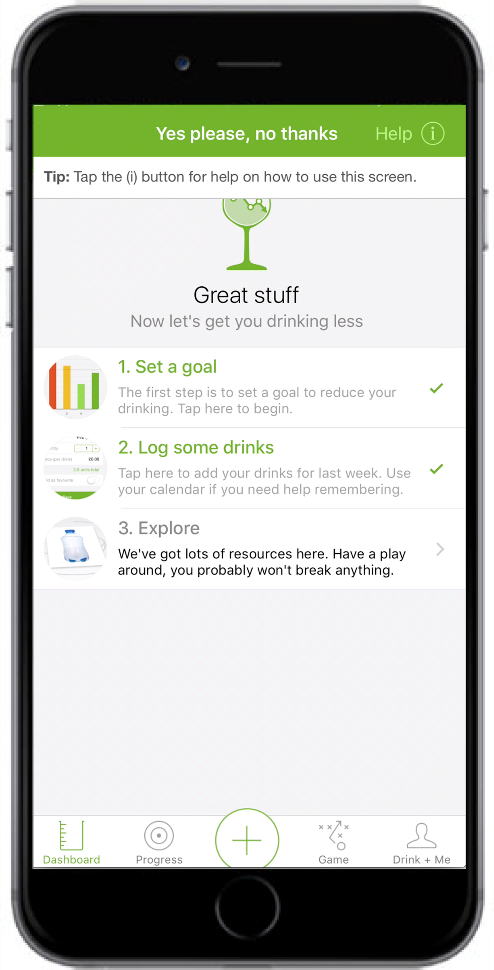f |
| --- | --- | --- |
| Users were asked to consent to notifications only when they were some way into using the app. Seeking permissions too early can dissuade users | The stepped guide gave users three tasks to familiarise themselves with the app. Completed steps were greyed-out and ticked. | The info button (top right) provided useful information about using the app. A message at the top brought its existence to user’s attention |

## Apple App Store listing

Text informs potential users that the app is part of an experiment

## Feedback given on AUDIT score

| AUDIT Score | Feedback given |
| --- | --- |
| Between 0-7 | {Green text)  Your results indicate you’re not at risk of physical and/or psychological alcohol-related harm.  Your score was X which lies in the range of 0-7 for this risk zone. |
| Between 8-15 | (Yellow text)  Your results indicate you’re putting yourself at increasing risk of physical and/or psychological alcohol-related harm.  Your score was X which lies in the range of 8-15 for this risk zone. |
| Between 16-19 | (Orange text)  Your results indicate you’re likely to be experiencing physical and/or psychological alcohol-related harm.  Your score was X which lies in the range of 16-19 for this risk zone. |
| Between 20-40 | (Red text)  You results indicate the possibility of alcohol dependence. You are welcome to continue to use this app though we strongly advise you to contact your GP for further support.  Your score was X which lies in the range of 20-40 for this risk zone. |

## User-selectable options for alcoholic drinks

| Type | Options | | Volume |
| --- | --- | --- | --- |
| Beer | Ale  Lager  Stout  Craft | | Very small bottle (275ml)  ½ pint (284ml)  Small bottle (330ml)  Can (440ml)  Large bottle (500ml)  Large can (500ml)  Pint (568ml) |
| Cider | (None) | | Very small bottle (275ml)  ½ pint (284ml)  Small bottle (330ml)  Can (440ml)  Large bottle (500ml)  Large can (500ml)  Pint (568ml) |
| Wine | Red  White  Rose  Sparkling | | Small glass (125ml)  Medium glass (175ml)  ¼ bottle (187.5ml)  Large glass (250ml)  1/3 bottle (250ml)  ½ bottle (375ml) |
| Fortified wine | Sherry  Port  Madeira  Marsala | | Glass (150ml) |
| Spirits | Options  Whisky  Vodka  Rum  Gin | Add  Nothing (neat)  Coke  Lemonade  Lime  Juice  Diet coke  Diet lemonade  Tonic  Ginger beer/ale  Soda  Other | Single (25ml)  Double (50ml)  Triple (75ml) |
| Alcopops | None | | Small bottle (275ml)  Bottle (330ml) |

Items appear in the order presented in the app. Items in bold were selected by default.

## Goal feedback

| Criteria | Feedback |
| --- | --- |
| Goal exceeded by ≥ 20% once: | Overachiever! Goal smashed. Well done. |
| Goal exceeded by ≥ 20% twice in a row: | Whoa, there goes that goal again. Twice in a row too. Is this an unusual period or do you think the goal is a bit easy? You can make it harder if you like. |
| Goal hit; one of (delivered at random): | Get you! Good work on hitting your goal.  Congratulations on a great [week/month] of achievement. Feel proud? You should.  Goal hit. Good work. You’re great.  That’s your goal got! I’d pat you on the back if I had arms.  Well done, you hit your goal. Keep going. |
| Goal missed; one of (delivered at random): | Didn’t quite make this one. Close though. You can do this.  Just missed this goal. It’s definitely within reach though.  Nearly made it. Just need to do a bit more and you’ll make it next time.  That was close! Won’t take much more to get that glorious green tick.  Almost! Bit more of a push and you’ll get this goal. |
| Goal missed by ≥ 20%: | You didn’t hit your goal this week. No problem, keep going. |
| Goal missed by ≥ 20% twice in a row: | Looks like you’re having a bit of difficulty with this one. Is it an unusual period, or do you think the goal is a bit much of a stretch? You can make it a slightly easier if you like. |

If goals were exceeded or missed by ≥ 20% twice in a row the underlined text in the feedback provided linked to the Goal Settting screen where a goal could be be amended.

| Icon | Represents |
| --- | --- |
|  | Goal exceeded by ≥ 20% |
|  | Goal hit |
|  | Goal missed |
|  | Goal missed by ≥ 20% |

## References

1. Department of Health. UK Chief Medical Officers’ Alcohol Guidelines Review. Summary of the proposed new guidelines. 2016.

2. Royal College of Physicians. RCP comments on the frequency of alcohol consumption.

3. Crane D, Garnett C, Brown J, West R, Michie S. Factors influencing usability of a smartphone app to reduce excessive alcohol consumption: think-aloud and interview studies. Front Public Heal. 2017;5(39).

4. Taylor MJ, Vlaev I, Maltby J, Brown GDA, Wood AM. Improving social norms interventions: Rank-framing increases excessive alcohol drinkers’ information-seeking. Heal Psychol. 2015;34(12):1200.

5. Beard E, Brown J, West R, Acton C, Brennan A, Drummond C, et al. Protocol for a national monthly survey of alcohol use in England with 6-month follow-up: “The Alcohol Toolkit Study”. BMC Public Health. 2015;15(1):230.

6. Schultz PW, Nolan JM, Cialdini RB, Goldstein NJ, Griskevicius V. The constructive, destructive, and reconstructive power of social norms. Psychol Sci. 2007;18(5):429–34.

7. Neighbors C, Jensen M, Tidwell J, Walter T, Fossos N, Lewis M a. Social-norms interventions for light and nondrinking students. Gr Process Intergr Relations. 2011;14(5):651–69.

8. Wiers RW, Rinck M, Kordts R, Houben K, Strack F. Retraining automatic action-tendencies to approach alcohol in hazardous drinkers. Addiction. 2010;105(2):279–87.

9. Pronk T, van Deursen DS, Beraha EM, Larsen H, Wiers RW. Validation of the Amsterdam Beverage Picture Set: A Controlled Picture Set for Cognitive Bias Measurement and Modification Paradigms. Alcohol Clin Exp Res. 2015;39(10):2047–55.

10. van Deursen DS, Salemink E, Smit F, Kramer J, Wiers RW. Web-based cognitive bias modification for problem drinkers: protocol of a randomised controlled trial with a 2x2x2 factorial design. BMC Public Health. 2013;13(1):674.

11. Kersbergen I, Woud ML, Field M. The validity of different measures of automatic alcohol action tendencies. Psychol Addict Behav. 2015;29(1):225–30.

12. Lister C, West JH, Cannon B, Sax T, Brodegard D. Just a Fad? Gamification in Health and Fitness Apps. JMIR Serious Games. JMIR Serious Games; 2014 Aug 4;2(2):e9.

13. Crane D, Garnett C, Brown J, West R, Michie S. Behavior change techniques in popular alcohol reduction apps: content analysis. J Med Internet Res. 2015;17(5):e118.

14. Linke S, McCambridge J, Khadjesari Z, Wallace P, Murray E. Development of a psychologically enhanced interactive online intervention for hazardous drinking. Alcohol Alcohol. 2008;43(6):669–74.

15. Dulin PL, Gonzalez VM, Campbell K. Results of a Pilot Test of a Self-Administered Smartphone-Based Treatment System for Alcohol Use Disorders: Usability and Early Outcomes. Subst Abus. 2014;35(2):168–75.

16. Milward J, Khadjesari Z, Fincham-Campbell S, Deluca P, Watson R, Drummond C. User Preferences for Content, Features, and Style for an App to Reduce Harmful Drinking in Young Adults: Analysis of User Feedback in App Stores and Focus Group Interviews. JMIR mhealth uhealth. 2016;4(2):e47.

17. Krebs P, Duncan DT. Health App Use Among US Mobile Phone Owners: A National Survey. JMIR mHealth uHealth. 2015;3(4):e101.

18. Harkin B, Webb TL, Chang BPI, Prestwich A, Conner M, Kellar I, et al. Does monitoring goal progress promote goal attainment? A meta-analysis of the experimental evidence. Psychol Bull. 2016;142(2):198.

19. The Wine and Spirit Trade Association. UK Wine and Spirits: Market Overview. Int Wine Spirit Res. 2013;

20. West R. The PRIME theory of motivation as possible foundation for the treatment of addiction. In: Bickel W, editor. Addiction Treatment: Science and policy for the Twenty-first Century. Baltimore, MD, US: Johns Hopkins University Press; 2007. p. 24–34.

21. West R, Hardy A. Theory of Addiction. Alcohol and Alcoholism. Oxford, UK: Blackwell Publishing; 2006. 161-161 p.

22. Hull JG, Slone LB. Alcohol and self-regulation. Handbook of self-regulation: Research, theory, and applications. 2004. p. 466–91.

23. Baumeister RF, Heatherton TF. Self-Regulation Failure: An Overview. Psychol Inq. 1996 Jan;7(1):1–15.

24. Field M, Schoenmakers T, Wiers RW. Cognitive processes in alcohol binges: a review and research agenda. Curr Drug Abuse Rev. 2008;1(3):263–79.

25. Field M, Cox WM. Attentional bias in addictive behaviors: A review of its development, causes, and consequences. Drug Alcohol Depend. 2008;97(1–2):1–20.

26. Jones S, Casswell S, Zhang JF. The economic costs of alcohol-related absenteeism and reduced productivity among the working population of New Zealand. Addiction. 1995;90:1455–61.

27. Crofton J. Extent and costs of alcohol problems in employment: a review of British data. Alcohol Alcohol. 1987;22:321–5.

28. Prat G, Adan A, Sánchez-Turent M. Alcohol hangover: A critical review of explanatory factors. Human Psychopharmacology. 2009. p. 259–67.

29. Verster JC, Stephens R, Penning R, Rohsenow D, McGeary J, Levy D, et al. The alcohol hangover research group consensus statement on best practice in alcohol hangover research. Current drug abuse reviews. 2010. p. 116–26.

30. Singleton R a, Wolfson AR. Alcohol consumption, sleep, and academic performance among college students. J Stud Alcohol Drugs. 2009;70(1997):355–63.

31. Yesavage JA, Leirer VO. Hangover effects on aircraft pilots 14 hours after alcohol ingestion: A preliminary report.

32. Pittler MH. Interventions for preventing or treating alcohol hangover: systematic review of randomised controlled trials. BMJ. 2005 Dec;331(7531):1515–8.

33. Howland J, Rohsenow DJ, Greece JA, Littlefield CA, Almeida A, Heeren T, et al. The effects of binge drinking on college students’ next-day academic test-taking performance and mood state. Addiction. 2010 Apr;105(4):655–65.

34. Swift R, Davidson D. Alcohol hangover: mechanisms and mediators. Alcohol Health Res World. 1998;22:54–60.

35. Slutske WS, Piasecki TM, Hunt-Carter EE. Development and initial validation of the Hangover Symptoms Scale: prevalence and correlates of Hangover Symptoms in college students. Alcohol Clin Exp Res. 2003;27(9):1442–50.

36. Wiese JG, Shlipak MG, Browner WS. The alcohol hangover. Annals of Internal Medicine. 2000. p. 897–902.

37. Vitiello M V. Sleep, alcohol and alcohol abuse. Addict Biol. 1997 Apr;2(2):151–8.

38. Roehrs T, Roth T. Sleep, sleepiness, sleep disorders and alcohol use and abuse. Sleep Med Rev. Elsevier; 2001 Aug;5(4):287–97.

39. Park DH, Yu J, Ryu SH. Alcohol and Sleep. Sleep Med Psychophysiol. 2006 Jun;13(1):5–10.

40. Locke EA, Latham GP. Building a practically useful theory of goal setting and task motivation. A 35-year odyssey. Am Psychol. 2002;57:705–17.

41. Bandura A, Cervone D. Self-evaluative and self-efficacy mechanisms governing the motivational effects of goal systems. J Pers Soc Psychol. 1983;45(5):1017–28.

42. Klinger E. Consequences of commitment to and disengagement from incentives. Psychological Review. 1975. p. 1–25.

43. Wortman CB, Brehm JW. Responses to Uncontrollable Outcomes: An Integration of Reactance Theory and the Learned Helplessness Model. Adv Exp Soc Psychol. 1975;8:277–336.

44. Carver CS, Scheier MF. Self-Regulation of Action and Affect. In: Vohs KD, Baumeister RF, editors. Handbook of self-regulation: Research, theory and application. Guilford Press; 2011. p. 3–21.

45. Sobell LC, Sobell MB. Timeline Follow-Back. Measuring alcohol consumption. Humana Press; 1992. p. 41–72.

46. Adams SL, McNeil DW. Negative alcohol expectancies reconsidered. Psychol Addict Behav. 1991;5(1):9–14.

47. Leigh BC, Stacy AW. Alcohol expectancies and drinking in different age groups. Addiction. 2004;99(2):215–27.

48. Leigh BC, Stacy AW. Alcohol outcome expectancies: Scale construction and predictive utility in higher order confirmatory models. Psychol Assess. 1993;5(2):216–29.

49. Brown SA, Christiansen BA, Goldman MS. The Alcohol Expectancy Questionnaire: An Instrument for the Assessment of Adolescent and Adult Alcohol Expectancies. J Stud Alcohol Drugs. 1987;48(5):483.

50. Steele CM. The psychology of self-affirmation: Sustaining the integrity of the self. In: Berkowitz L, editor. Advances in Experimental Social Psychology. New York: Academic Press; 1988. p. 261–302.

51. Epton T, Harris PR. Self-affirmation promotes health behavior change. Heal Psychol. 2008;27(6):746–52.

52. Armitage CJ, Harris PR, Hepton G, Napper L. Self-affirmation increases acceptance of health-risk information among UK adult smokers with low socioeconomic status. Psychol Addict Behav. 2008;22(1):88.

53. Harris PR, Napper L. Self-affirmation and the biased processing of threatening health-risk information. Personal Soc Psychol Bull. 2005;31(9):1250–63.

54. MCQueen A, Klein WMP. Experimental manipulations of self-affirmation: A systematic review. Self Identity. 2006;5(4):289–354.

55. Zarrella D. Which Types of Form Fields Lower Landing Page Conversions? Hubspot.com. 2010.

56. Babor T, Higgins J, Saunders J, Monteiro M. AUDIT: the alcohol use disorders identification test guidelines for use in primary care. 2nd ed. World Health Organisation. Geneva: Switzerland; 2001. 1-40 p.

57. Crane D, Garnett C, Brown J, Kaner E, Beyer F, Muirhead C, et al. Behaviour change techniques used in digital interventions to reduce excessive alcohol consumption: a meta-regression. Manuscr Prep.

58. Forrester: HTML5 apps still not as good as native apps | InfoWorld.

59. The Advantages of Native Apps | Two Toasters.

60. Mobile applications: native v Web apps - what are the pros and cons? | mobiForge.

61. HTML5 vs native apps: key considerations for your mobile strategy | Media Network | Guardian Professional.

62. HTML5 vs. Native vs. Hybrid Mobile Apps.

63. HTML5 vs. PhoneGap vs. Xamarin vs. Native - Sahil Malik | blah.winsmarts.com.

64. comScore Reports July 2014 U.S. Smartphone Subscriber Market Share - comScore, Inc.

65. The Android Browser by HTML5test.

66. Fowler M, Highsmith J, Beck K, Beedle M, van Bennekum A, Cockburn A, et al. The agile manifesto. Software Development. 2001.

67. O’Connell C. 23% of Users Abandon an App After One Use. localytics.com. 2016.

68. The importance of App Store reviews - Cowly Owl.

69. Microsoft UK Developers. App ratings and reviews - how important are they? [Internet]. [cited 2016 Jan 14]. Available from: http://www.microsoft.com/en-gb/developers/articles/week02jun14/app-ratings-and-reviews-how-important-are-they

70. Garrett JJ. The Elements of User Experience : User-Centered Design for the Web and Beyond. New York, NY: Pearson Education; 2010. 192 p.

71. Yardley L, Morrison L, Bradbury K, Muller I. The person-based approach to intervention development: application to digital health-related behavior change interventions. J Med Internet Res. 2015;17(1):e30.

72. Cugelman B, Thelwall M, Dawes P. The Dimensions of Web Site Credibility and Their Relation to Active Trust and Behavioural Impact. Commun Assoc Inf Syst. 2009;24:455–72.

73. Pellegrini C a, Steglitz J, Hoffman S a. e-Health intervention development: a synopsis and comment on “What Design Features are Used in Effective e-Health Interventions? A Review Using Techniques From Critical Interpretive Synthesis”. Transl Behav Med. 2014;4(4):342–5.

74. iOS Human Interface Guidelines. Apple.

75. Doward J, Sandhu R. Real ale sales on the rise as Britain’s beer drinkers tire of lager. The Guardian. 2010.
